# N6-methyladenosine related gene expression signatures for predicting the overall survival and immune responses of patients with colorectal cancer

**DOI:** 10.3389/fgene.2023.885930

**Published:** 2023-03-03

**Authors:** Lili Yu, Lijuan Wang, Jing Sun, Xuan Zhou, Yeting Hu, Lidan Hu, Yazhou He, Chunqing Lin, Jie Chen, Xiaolin Xu, Malcolm G. Dunlop, Evropi Theodoratou, Kefeng Ding, Xue Li

**Affiliations:** ^1^ Department of Colorectal Surgery and Oncology, Key Laboratory of Cancer Prevention and Intervention, Ministry of Education, The Second Affiliated Hospital, Zhejiang University School of Medicine, Hangzhou, China; ^2^ Analytics of The Second Affiliated Hospital and Department of Big Data in Health Science School of Public Health, Center of Clinical Big Data, Zhejiang University School of Medicine, Hangzhou, China; ^3^ The Children’s Hospital, Zhejiang University School of Medicine, National Clinical Research Center for Child Health, Hangzhou, China; ^4^ Department of Oncology, West China School of Public Health and West China Fourth Hospital, Sichuan University, Chengdu, Sichuan, China; ^5^ National Cancer Center, National Clinical Research Center for Cancer, and Cancer Hospital, Chinese Academy of Medical Sciences and Peking Union Medical College, Beijing, China; ^6^ Center for Global Health, Zhejiang University, Hangzhou, China; ^7^ Cancer Research UK Edinburgh Centre, Medical Research Council Institute of Genetics and Cancer, University of Edinburgh, Edinburgh, United Kingdom; ^8^ Centre for Global Health, Usher Institute, University of Edinburgh, Edinburgh, United Kingdom; ^9^ Cancer Center, Zhejiang University, Hangzhou, China

**Keywords:** colorectal cancer, prognostic risk score, gene expression, overall survival, immune responses

## Abstract

N6-methyladenosine (m6A) modification has been demonstrated to exhibit a crucial prognostic effect on colorectal cancer (CRC). Nonetheless, potential mechanism of m6A in survival rate and immunotherapeutic response remains unknown. Here we investigated the genes associated with m6A regulators and developed a risk score for predicting the overall survival (OS) of CRC patients. RNA-seq transcriptomic profiling data of COAD/READ samples were obtained from The Cancer Genome Atlas (TCGA) database. Absolute Shrinkage and Selection Operator (LASSO)- Cox regression analysis was conducted to identify the m6A-related gene expression signatures and the selected genes were inputted into stepwise regression to develop a prognostic risk score in TCGA, and its predictive performance of CRC survival was further validated in Gene Expression Omnibus (GEO) datasets. According to our results, the risk score comprising 18 m6A-related mRNAs was significantly associated with CRC survival in both TCGA and GEO datasets. And the stratified analysis also confirmed that high-risk score acted as a poor factor in different age, sex, T stage, and tumour, node, metastasis (TNM) stages. The m6A-related prognostic score in combination with clinical characteristics yielded time-dependent area under the receiver operating characteristic curve (AUCs) of 0.85 (95%CI: 0.79–0.91), 0.84 (95%CI: 0.79–0.90) and 0.80 (95%CI: 0.71–0.88) for the prediction of the 1-, 3-, 5-year OS of CRC in TCGA cohort. Furthermore, mutation of oncogenes occurred more frequently in the high-risk group and the composition of immune cells in tumour microenvironment (TME) was significantly distinct between the low- and high-risk groups. The low-risk group had a lower microsatellite instability (MSI) score, T-cell exclusion score and dysfunction score, implying that low-risk patients may have a better immunotherapy response than high-risk patients. In summary, a prognostic risk score derived from m6A-related gene expression signatures could serve as a potential prognostic predictor for CRC survival and indicator for predicting immunotherapy response in CRC patients.

## Introduction

Colorectal cancer (CRC) is the third most common cancer and a leading cause of cancer mortality worldwide (Bray et al., 2018). Although the survival time of CRC patients has been significantly extended by clinical treatment, the 5-year OS of CRC patients is still not ideal, with a rate of approximate 68% (Yuan et al., 2021). Presently, emerging evidence has shown that the discovery and application of molecular biomarkers may provide important clinical implications on the prognosis and treatment of CRC patients (Bramsen et al., 2017).

N6-methyladenosine (m6A) is one of the most prominent and abundant forms of internal RNA modification involved in stabilizing transcripts, affecting degradation process of mRNA and non-coding RNA, and promoting initiate translational efficiency (Wang et al., 2014; Liu et al., 2015; Ma et al., 2019; Sun et al., 2019). This modification regulated by methyltransferases, demethylases, and binding proteins, is a dynamic reversible process in mammalian cells, which are also known as “writers”, “erasers”, and “readers” (Yang et al., 2018). Of note, some of these effects are mediated by m6A “readers” proteins, which can selectively recognize m6A and exert a regulatory function on the m6A-marked mRNAs (He and He, 2021). Several recent systematic studies of the cross-link between m6A modification, substrate genes, and post-modification regulation to reveal the biological role of m6A in cancer development comprehensively (Luo et al., 2018; Chang et al., 2019). METTL3 enhances translation of oncogene BRD4 through forming an mRNA loop in lung adenocarcinoma, and promotes expression of SRY (sex-determining region Y)-box 2 (SOX2) through IGF2BP2-directed suppression of RNA degradation in CRC (Choe et al., 2018; Li et al., 2019). The m6A functions induced by m6A related modification enzymes can be influenced by environmental exposure (e.g., reactive oxygen species, inflammation, and cyclobutene pyrimidine dimers) and genomic signals (e.g., somatic mutation), thereby epigenetics provides a molecular basis for cancer development (Li D. et al., 2020; Rong et al., 2021). To date, accumulating evidence demonstrated that dysregulated m6A methylation modification is associated with multiple biological processes, including dysregulate cell proliferation and death, immunomodulatory abnormality and tumour malignant progression (Fu et al., 2014), thus could be closely related to a variety of human diseases, in particular cancer (Hong, 2018). For instance, it is shown that YTHDF2 may act as a tumour suppressor to restrain cell proliferation and growth *via* destabilizing the EGFR mRNA in hepatocellular carcinoma (Zhong et al., 2019). Previous study on the pathological role of m6A modification in CRC reported that METTL3, one of m6A regulators, directly induced m6A-glucose transporter 1 (GLUT1)-mammalian target of rapamycin complex 1 (mTORC1) axis to promote CRC initiation and progression (Chen et al., 2021). Likewise, another experimental study showed that METTL3 stabilizes HK2 and SLC2A1 (GLUT1) expression in CRC through an m6A-IGF2BP2/3- dependent mechanism, thereby pointing to the notion that m6A modification is a promising indicator of controlling human CRC aggressiveness (Shen et al., 2020). However, the specific role of m6A regulators in the dysregulation of mRNAs in CRC prognosis remains unclear.

The tumour microenvironment (TME), which is composed of various cancer cells, stromal cells, and distinct recruited cells (infiltrating immune cells, bone marrow-derived cells), plays a vital role in tumour progression and affects the clinical benefit from novel strategies of immunological checkpoint blockade (ICB) (Hanahan and Coussens, 2012; Topalian et al., 2012). ICB treatment, such as those programmed cell death protein 1 (PD1), programmed death-ligand 1 (PDL1) and cytotoxic T-lymphocyte antigen 4 (CTLA-4) is now the first class of immunotherapy to have a broad impact on survival for cancer patients, across a wide variety of tumour histologies and treatment settings (Littman, 2015; Lonberg and Korman, 2017; Ribas and Wolchok, 2018; Wieder et al., 2018). Emerging studies have made efforts to understand the heterogeneity and complexity of the TME by elaborate analysis of m6A modification, therefore improving immunotherapy strategies (Li N. et al., 2020). Predicting the immunotherapy response of CRC patients based on multiple m6A-related biomarkers has the potential to develop a personalised treatment strategy and therefore to increase the success of ICB (Fang and Declerck, 2013; Quail and Joyce, 2013; Binnewies et al., 2018).

In this study, we sought to elucidate the m6A related mRNAs signatures for predicting the overall survival (OS) and immune responses of CRC patients using transcriptome data from The Cancer Genome Atlas (TCGA) (2012) (Cancer Genome Atlas Network, 2012) and Gene Expression Omnibus (GEO) (Smith et al., 2010; Marisa et al., 2013) datasets. We focused on the m6A-related genes and developed a multivariate Cox prediction model for the OS of CRC patients and examined its prognostic ability in immunotherapy response. We additionally explored the candidate drugs targeting these m6A-related gene signatures using the publicly available Genomics of Drug Sensitivity in Cancer (GDSC) database for predicting drug sensitivity (Yang et al., 2013). Findings from this study are helpful to predict the prognosis of CRC and develop personalized CRC treatment strategies.

## Materials and methods

### Study population and datasets

A study sample of 644 CRC patients from the TCGA was used as a training dataset. RNA-seq [Fragments Per Kilobase of transcript per Million mapped reads (FPKM normalized)] were acquired from Genomic Data Commons Data Portal (https://portal.gdc.cancer.gov/) using the R package “TCGAbiolinks”, which was specifically developed for integrative analysis with Genetic Data Commons (GDC) data (Colaprico et al., 2016). Then FPKM values were transformed into transcripts per kilobase million (TPM) values. The corresponding clinicopathological information and somatic mutation data of CRC patients were obtained from the cBioPortal database (https://portal.gdc.cancer.gov/). Two study samples (GSE39582, N = 566; GSE17536, N = 177) from the GEO database were used as validation datasets, and their normalized microarray gene expression data and clinicopathological data were obtained online (https://www.ncbi.nlm.nih.gov/geo/). Those RNA probe sets were re-annotated using the Ensemble database (http://www.ensembl.org). CRC patients with missing survival data and OS values or OS < 30 days were excluded in order to reduce statistical bias in this analysis.

### Identification of m6A-related prognostic genes

The expression matrices of 21 m6A regulators were retrieved from the TCGA, including the expression data of eight writers (*METTL3, METTL14, METTL16, RBMX2, RBM15B, WTAP, KIAA1429*, and *ZC3H13*), two erasers (*FTO* and *ALKBH5*), and eleven readers (*YTHDF1, YTHDF2, YTHDF3, YTHDC1, YTHDC2, IGF2BP1, EMR1, LRPPRC, HNRNPA2B1, HNRNPC,* and *ELAVL1*). Based on the RNA-seq data, Pearson’s correlation analysis was firstly implemented to identify m6A-related genes, using the criteria of |Pearson R| >0.3 and *p* < 0.001. Univariable and multivariable Cox regression models (false discovery rate, FDR<0.05) and the least absolute shrinkage and selection operator (LASSO) Cox regression were conducted subsequently to select the m6A-related prognostic genes that were distinctly related to the OS of CRC patients. The proteins of m6A regulators and m6A-related prognostic genes in CRC and normal tissues were further examined by using immunohistochemistry data in the human Protein Atlas (HPA) (https://www.proteinatlas.org/) database, which provided expression levels of 24,000 protein in different tissues and cells (Uhlen et al., 2017).

### Development and validation of the m6A-related prognostic risk score

A weighted prognostic risk score of m6A-related gene expression was constructed based on the following formula: Risk score = $\overset{n}{\underset{i=1}{\sum }}Coef\;\left(Genei\right)\times Expr\left(Genei\right)$ , where Coef (Gene_i_) was the coefficient of genes correlated with CRC survival, and Expr (Gene_i_) was the expression of genes. The prognostic value of the risk score was evaluated by Kaplan-Meier survival curves with log-rank tests in both TCGA and GEO study samples. Multivariate Cox regression analysis was performed to evaluate the prediction performance of the m6A-related prognostic risk score. Patients with CRC were further stratified into low- and high-risk groups based on the median value of the prognostic risk score of m6A-related genes.

### Analysis of the molecular characteristics in the low- and high-risk groups

To explore the biological function and alternative pathways of these m6A-related gene signatures, we performed a co-expression and pathway enrichment analysis based on the TCGA database, using the Kyoto Encyclopaedia of Genes and Genomes Pathway (KEGG pathway) as reference (Kanehisa and Goto, 2000). Linear regression was performed to detect co-expressed genes (FDR<0.05). In the gene mutation analysis, we obtained somatic mutation information from the cBioPortal database, and the quantity and quality of gene mutations were analysed in low- and high-risk groups by using the Maftools package in R.

### Exploration of immunotherapeutic response between low- and high-risk groups

To depict immune characteristics of CRC patients, the entire expression data were imported into CIBERSORT (https://cibersort.stanford.edu/) and a deconvolution algorithm using support vector regression was used and iterated 1,000 times to determine the relative proportions of 22 immune cell types in tumours. The relative proportions of immune cell types and clinicopathologic factors were compared between the low- and high-risk groups. The tumour Immune Dysfunction and Exclusion (TIDE) score was calculated online (http://tide.dfci.harvard.edu/) to predict the likelihood of immunotherapeutic response between the low- and high-risk groups.

### Prediction of potential compounds targeting therapeutic sensitivity in CRC patients

To obtain potential compounds with differential therapeutic sensitivity, we investigated the predictive capacity of the low- and high-risk groups in responding immunotherapy. The 50% inhibiting concentration half-maximal inhibitory concentration (IC_50_) value of 138 anti-cancer drugs was inferred from the GDSC website based on the COAD/READ dataset of the TCGA project. The “pRRophetic” algorithm (Geeleher et al., 2014) was used to predict the IC_50_ of compounds in the low- and high-risk groups separately.

### Statistical analysis

An independent *t*-test was performed to compare continuous variables between two groups. Categorical data were tested using the *χ* ^2^ test. Pearson correlation analysis was implemented to identify m6A-related genes (with the | Pearson r | >0.05 and *p* < 0.001). Univariate survival analysis was performed by K-M survival analysis with the log-rank test to calculate the significance of differences in the OS. Multivariate survival analysis was performed using the Cox regression model to estimate the hazard ratio (HR). The time-dependent area under the receiver operating characteristic curve (AUC) was estimated to evaluate the predictive power of the risk score and TNM stage to the OS. Stratification analysis was performed to investigate the survival difference in subgroups, including age, sex, T stage, N stage, M stage, American Joint Committee on Cancer (AJCC) TNM stage and radiation therapy history. A nomogram of the risk score and other predictors was set up accordingly for the prediction of the 1-, 3-, 5- year OS. The *p* values were two-sided and *p* < 0.05 was considered as statistically significant.

## Results

### Landscape of genetic variation of m6A regulators in CRC patients

**FIGURE 1:**
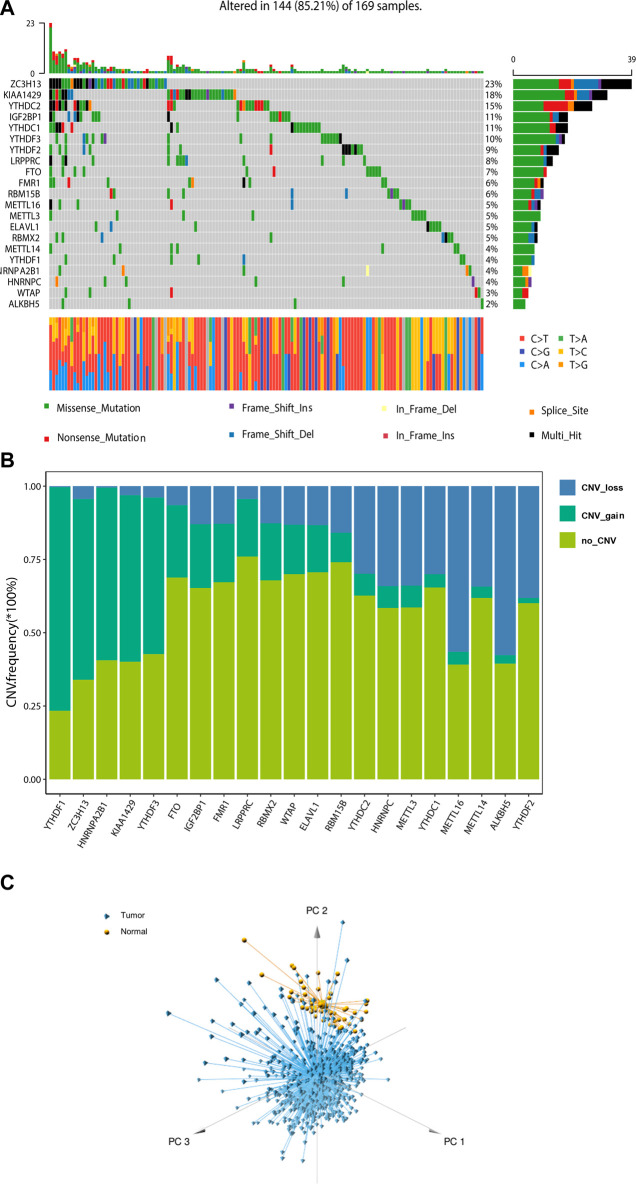
Landscape of genetic of m6A regulators in colorectal cancer. **(A)** The mutation frequency of 21 m6A regulators in 169 patients with CRC from TCGA cohort. **(B)** Bar graphs showing the frequency of CNV gain (green), loss (blue) and non CNV (yellow) of m6A regulators in TCGA-COAD/READ cohort. **(C)** Principal component analysis for the expression profiles of 21 m6A regulators to distinguish tumours from normal samples in TCGA cohort.

A total of 21 m6A regulators, namely, 8 “writers”, 2 “erasers”, and 11 “readers”, were included in this study. We firstly assessed the prevalence of somatic mutations and copy number variations (CNV) of these 21 m6A regulators. Among the 551 samples, 169 (30.67%) had mutations in any of the m6A modification regulators (Figure 1A; Table 1). *ZC3H13* exhibited the highest mutation frequency (23%) followed by *KIAA1429* (18%) and *YTHDC2* (15%), while demethylases *ALKBH5* (2%) and *WTAP* (3%) showed low number of mutations in CRC samples. Somatic copy number alterations of these m6A regulators were then examined, and we found that *METTL14* (34%), *METTL16* (56%), *ALKBH5* (58%) and *YTHDF2* (38%) had a widespread frequency of CNV deletions (Figures 1B, C). To ascertain whether the above genetic variations influenced the expression of m6A regulators in CRC patients, we investigated the mRNA alterations of the m6A regulators between paired normal and tumour samples of CRC patients. This showed that alterations of CNV were prominent factors, resulting in perturbations on the m6A regulators expression. Compared to the normal colon tissues, regulators with CNV gain demonstrated markedly higher expression in CRC tissues (e.g., *YTHDF1* and *KIAA1429*) (Figure 1B; Supplementary Figure S1). And *vice versa*, some regulators showed downregulated mRNA expression but with high frequency of CNV loss (e.g., *ALKBH5*). This analysis showed the high heterogeneity of genetic and expressional alteration landscape of m6A regulators between normal and tumour samples, demonstrating that the expression imbalance of m6A regulators may be important in the initiation and progression of CRC.

**TABLE 1:** Correlation between subgroups and clinicopathological factors in the TCGA cohort.

Variable	Overall (*n* = 551)	High-risk (*n* = 275)	Low-risk (*n* = 276)	*p*-value		
Age, n (%)				0.730		
<60 years	164 (29.8%)	80 (29.1%)	84 (30.4%)			
≥60 years	387 (70.2%)	195 (70.9%)	192 (69.6%)			
Gender, n (%)				0.581		
Female	254 (46.1%)	130 (47.2%)	124 (44.9%)			
Male	297 (53.9%)	145 (52.7%)	152 (55.1%)			
Cancer type, n (%)				0.368		
COAD	408 (74.0%)	199 (72.4%)	209 (75.7%)			
READ	143 (26.0%)	76 (27.6)	67 (24.3%)			
AJCC stage, n (%)				<0.001		
I	96 (17.4%)	35 (12.7%)	61 (22.1%)			
II	201 (36.5%)	85 (30.9%)	116 (42.0%)			
III	163 (29.6%)	98 (35.6%)	65 (23.6%)			
IV	79 (14.3%)	50 (18.2%)	29 (10.5%)			
Not available	12 (2.2%)	7 (2.6%)	5 (1.8%)			
T stage, n (%)				0.004		
T1-2	116 (21.1%)	44 (16.0%)	72 (26.1%)			
T3-4	435 (78.9%)	231 (84.0%)	204 (73.9%)			
M stage, n (%)				0.020		
M0	411 (74.6%)	193 (70.2%)	218 (79.0%)			
M1-x	135 (24.5%)	79 (28.7%)	56 (20.3%)			
Not available	5 (0.9%)	3 (1.1%)	2 (0.7%)			
N stage, n (%)				<0.001		
N0	314 (57.0%)	128 (46.5%)	186 (67.4%)			
N1-x	237 (43.0%)	147 (53.5%)	90 (32.6%)			
Radiotherapy, n (%)				0.301		
No	457 (83.0%)	224 (81.5%)	233 (84.4%)			
Yes	27 (4.9%)	16 (5.8%)	11 (4.0%)			
Not available	67 (12.1%)	35 (12.7%)	32 (11.6%)			
Survival status, n (%)				<0.001		
Alive	438 (79.5%)	189 (68.7%)	249 (90.2%)			
Dead	113 (20.5%)	86 (31.3%)	27 (9.8%)			

### Identification of m6A-related genes in patients with CRC

**FIGURE 2:**
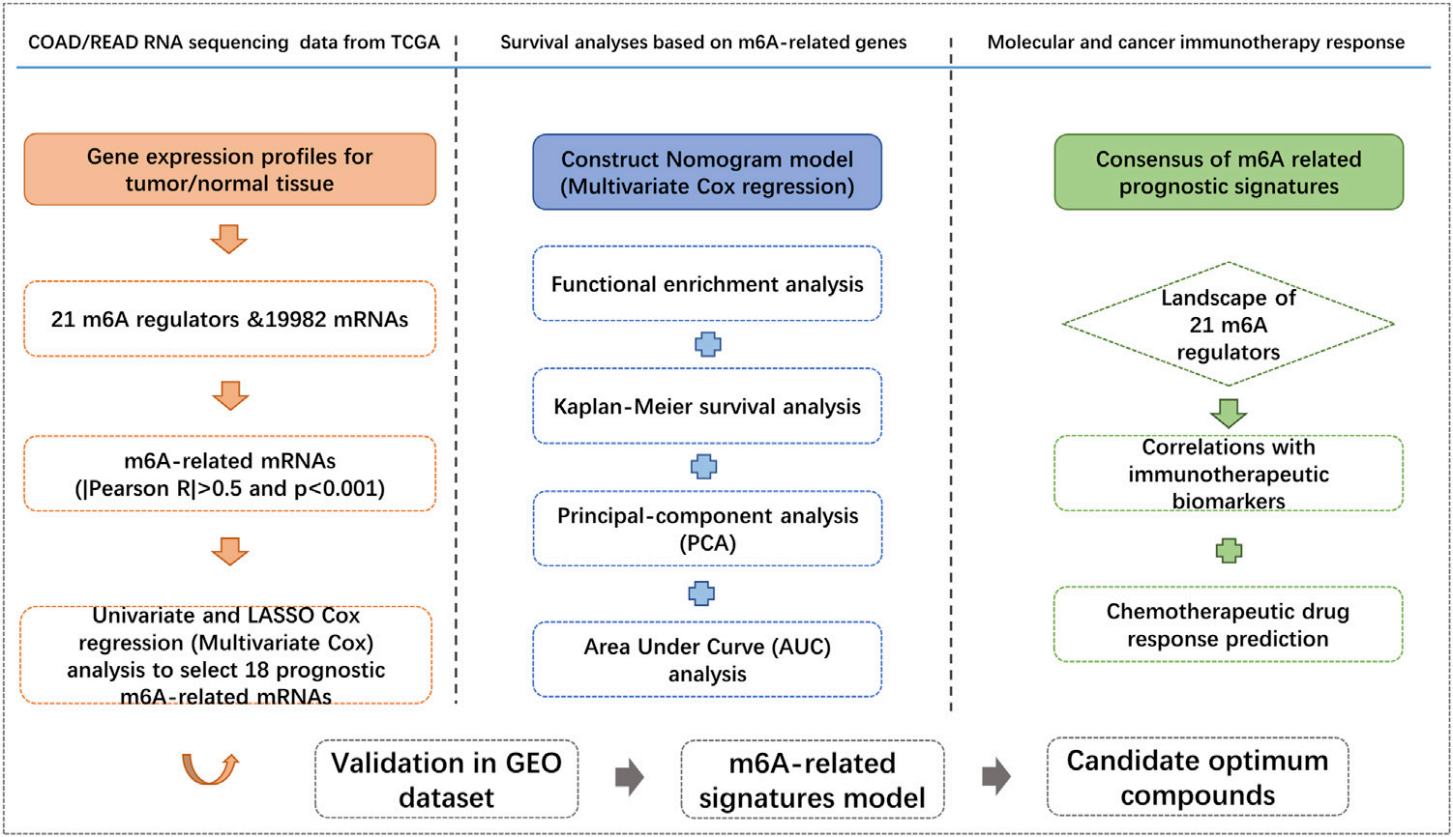
Flow chart of this study.

A total of 551 COAD/READ patients from the TCGA database were included in our study to calculate the prognostic risk score of m6A-related genes. The detailed workflow for risk model construction and subsequent analyses is shown in Figure 2. We abstracted the matrix expression of 21 m6A regulators and 19,982 mRNAs from the TCGA database. Correlations between these 21 m6A regulators and 19,982 mRNAs were examined and we identified 4,274 mRNAs that were significantly correlated with m6A regulators base on the criteria of |Pearson R|>0.5 and *p* < 0.001. To identify m6A-related genes that correlated with the OS of CRC patients, we screened from 4,274 m6A-associated mRNAs in the TCGA training set using univariate Cox regression analysis. At FDR<0.05, fifty-seven m6A-related mRNAs correlated significantly with OS (Supplementary Table S1).

### Construction of the prognostic risk score based on m6A-related gene expression signatures

**FIGURE 3:**
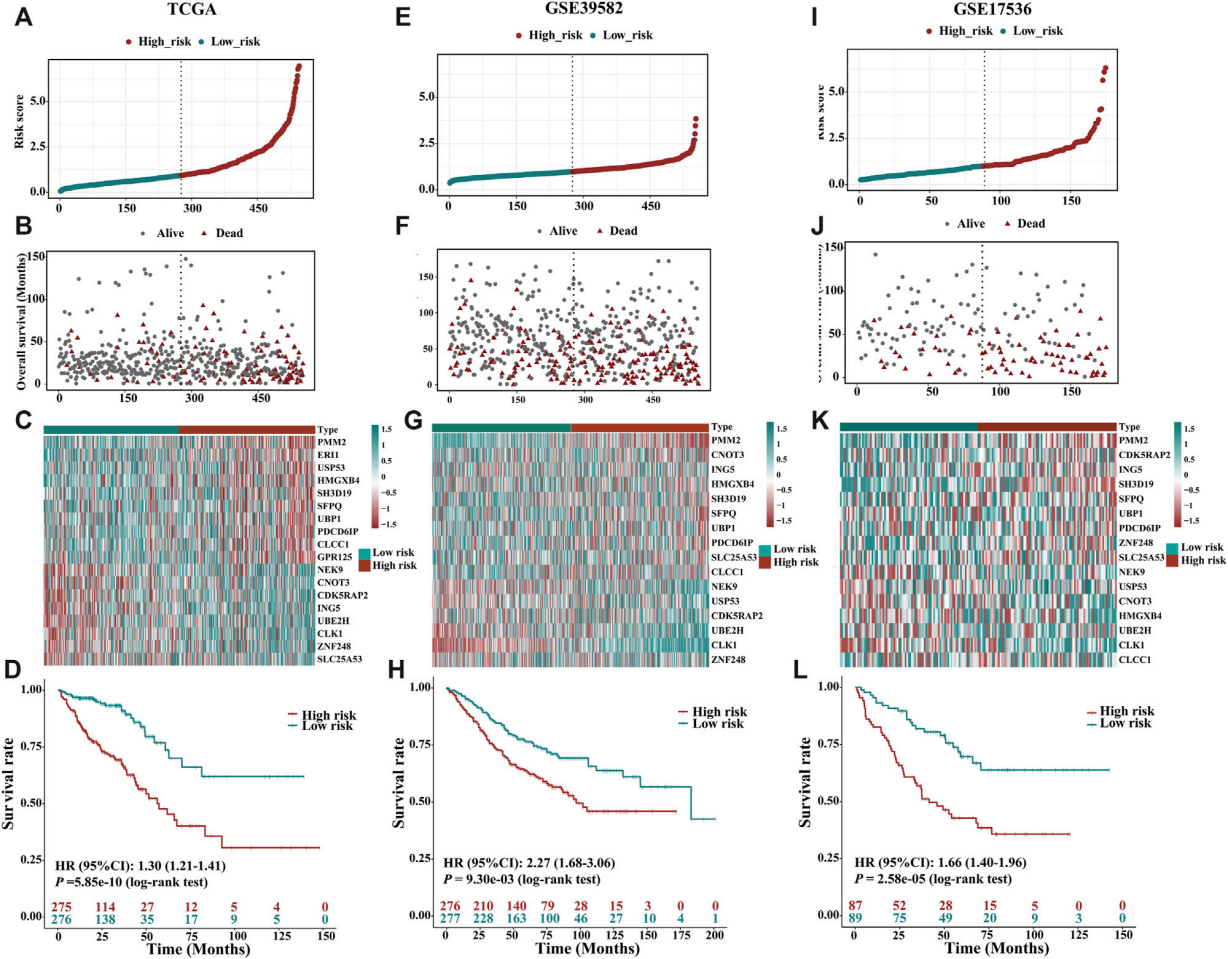
Prognostic value of the risk patterns of the 18 m6A-related gene signatures in the TCGA training dataset, GSE39582 and GSE17536 validation dataset. **(A)** Distribution of m6A-related gene expression model-based risk score for TCGA. **(B)** Different patterns of survival status and survival time between the high- and low-risk subgroups for TCGA. **(C)** Clustering analysis heatmap shows the expression standards of the 18 prognostic genes for each patient for TCGA. **(D)** Kaplan-Meier survival curves of the OS of patients in the high- and low-risk subgroups for TCGA. **(E)** Distribution of m6A-related gene expression model-based risk score for the GSE39582. **(F)** Different patterns of survival status and survival time between the high- and low-risk subgroups for the GSE39582. **(G)** Clustering analysis heatmap shows the expression standards of the 18 prognostic genes for each patient for the GSE39582. **(H)** Kaplan-Meier survival curves of the OS of patients in the high- and low-risk subgroups for the GSE39582. **(I)** Distribution of m6A-related gene expression model-based risk score for the GSE17536. **(J)** Different patterns of survival status and survival time between the high- and low-risk subgroups for the GSE17536. **(K)** Clustering analysis heatmap shows the expression standards of the 18 prognostic genes for each patient for the GSE17536. (**L**) Kaplan-Meier survival curves of the OS of patients in the high- and low-risk subgroups for the GSE17536.

To avoid overfitting, the LASSO-Cox regression was applied to optimise the selection of gene signatures in relation to the OS. Consequently, 18 m6A-related mRNAs (*PMM2*, *ERI1*, *NEK9*, *USP53*, *CNOT3*, *CDK5RAP2*, *ING5*, *HMGXB4*, *SH3D19*, *UBE2H*, *CLK1*, *SFPQ*, *UBP1*, *PDCD6IP*, Z*NF248*, *SCL25A53*, *CLCC1* and *GPR125*) were finally selected to construct a m6A-related prognostic risk score for CRC survival (Supplementary Figures S2A, S2B). The correlation between m6A regulators and m6A-related gene expression in the TCGA dataset is showed in Supplementary Figure S3. Twelve out of the 18 gene products and 17 out of the 21 m6A regulators were obtained from the HPA database, and the other six genes and four m6A regulators were not available or in low reliability. The immunohistochemistry-stained proteins of the 17 m6A regulators and 12 genes in CRC and normal tissues were shown in Supplementary Figures S4A, S4B. A weighted prognostic risk score of m6A-related gene expression was constructed based on the gene expression levels of the 18 selected markers. CRC patients were separated into high- and low-risk groups based on the median value of the prognostic risk score constructed by the m6A-related gene expression signatures. The distribution of risk scores between the low- and high-risk groups is depicted in Figure 3A, and the survival status and survival time of CRC patients in the low- and high-risk groups are shown in Figure 3B. The expression levels of the 18 m6A-related genes in the low- and high-risk groups are shown in Figure 3C. Kaplan–Meier survival curves showed that CRC patients with higher risk scores had worse clinical outcomes (lower OS rates and a shorter OS time, HR = 1.30, 95%CI: 1.21–1.41; *p* = 5.85e-10, log-rank test) (Figure 3D). Based on the entire gene expression profiles, 21 m6A regulators and the expression profile of the 18 m6A-related genes, PCA analysis was further conducted to test the difference between the low- and high-risk groups (Supplementary Figures S5A–C). As showed Supplementary Figures S5A, B, the gene expression profiles of the low- and high-risk groups were differently distributed (Supplementary Figures S5C).

### Validation of the prognostic risk score based on m6A-related gene expression signatures

Detailed clinicopathologic characteristics of CRC patients in TCGA and GEO datasets are shown in Table 1 and Table 2. The expression of 18 m6A-related genes was closely correlated with the OS of CRC patients as determined by K-M analysis (Supplementary Figures S6). According to the subgroups classified by sex, age, AJCC TNM stage or tumour stage, the OS of the low-risk group continued to be superior to that of the high-risk group (Supplementary Figures S7A–H). To validate the prognostic capability, we calculated the risk scores for CRC patients in two GEO (GSE39582, N = 553; GSE17536, N = 176) datasets using the same formula. As showed in Figures 3E–G; Figures 3I–K, patients stratified into the high-risk group had a significantly worse prognosis than those in the low-risk group (Figure 3H, HR = 2.27, 95%CI: 1.68–3.06, *p* = 9.30e-03, log-rank test; Figure 3L, HR = 1.66, 95%CI: 1.40–1.96, *p* = 2.58e-05, log-rank test), which was consistent with the results of TCGA dataset.

**TABLE 2:** Clinical information of CRC cohorts from GEO dataset.

Accession number	Platform	Tumor samples	Survival data	Stage	Gender	PMID
GSE39582	GPL570	566	553	T stage I-IV: 553	M:322; F:263	23700391
GSE17536	GPL570	177	176	Stage I-IV: 176	M:96; F:81	19914252

### Molecular characteristics of the low- and high-risk groups stratified by the prognostic risk score

**FIGURE 4:**
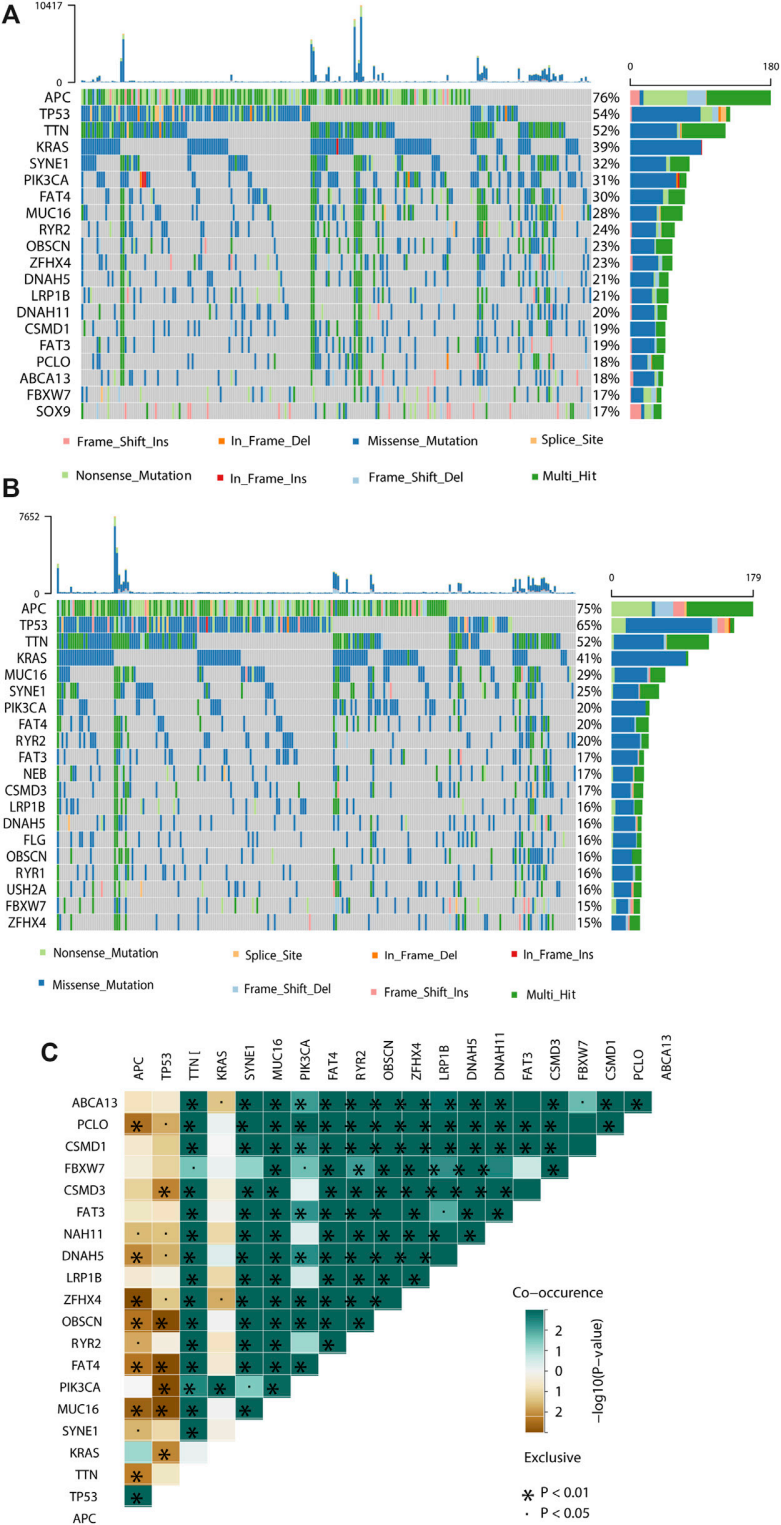
Molecular characteristics of different risk subgroups. **(A, B)** Waterfall plot displays tumour somatic mutation information of the genes with high mutation frequencies in the high-risk subgroup **(A)** and low-risk subgroup **(B)**. Mutated genes (rows, top 20) are ordered by mutation rate; samples (columns) are arranged to emphasize mutual exclusivity among mutations. The right shows the mutation percentage, and the top shows the overall number of mutations. The color coding indicates the mutation type. **(C)** The co-expression patterns of top 20 mutated genes in CRC patients.

To demonstrate the potential mechanisms and pathways involved in the molecular heterogeneity leading to the different outcomes between the low- and high-risk groups, we performed functional enrichment analysis with annotation of KEGG gene set. We found that m6A-related gene expression signatures were differentially enriched (FDR<0.05) in the pathways related to cancer, immune response, and neural signaling between the two groups (Supplementary Table S2), and pathways that more than half of the gene signatures enriched in were summarized in Supplementary Figure S8. When examining the somatic mutations, we found that the top 20 cancer driver genes mutated more frequently in the high-risk group than in the low-risk group, including four tumour suppressor genes (e.g., APC, TP53, LRP1B and ZFHX4) and the other sixteen genes (e.g., TTN, KARS, MUC16, SYNE1, PIK3CA, FAT4, RYR2 DNAH5, RYR1 and FBXW7, etc.) are oncogenes in disease development and progression (Figures 4A,B), and significant co-occurrences were also observed among mutations of these genes (as shown in Figure 4C).

### Estimation of the tumour immune microenvironment and cancer immunotherapy response

**FIGURE 5:**
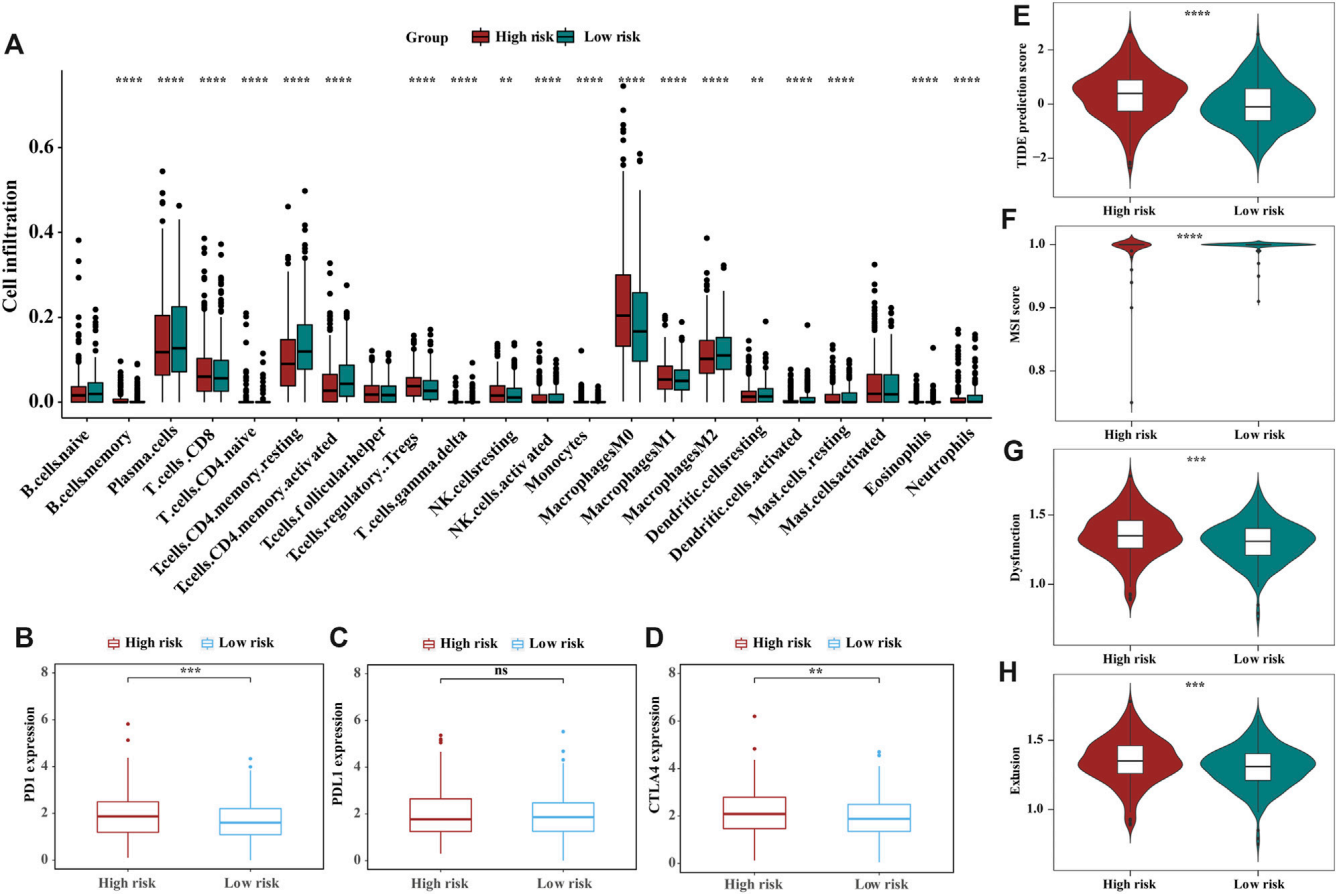
The landscape and estimation of the tumor immune microenvironment using the m6A-related gene signatures model. **(A)** The proportions of TME cells in different risk subgroups. Significant statistical differences between the two subgroups were assessed using the Wilcoxon test, the asterisks represented the statistical *p*-value (blank, not significant; *p < 0.05; **p < 0.01; ***p < 0.001; ****p < 0.0001). **(B–D)** Expression of the immune checkpoints PD1**(B)**, PDL1**(C)** and CTLA4 **(D)** between high- and low-risk groups. **(E–H)** TIDE **(E)**, MSI **(F)**, and T-cell exclusion **(G)** and dysfunction **(H)** score in the high- and low-risk patients. The scores between the two risk subgroups were compared through the Wilcoxon test (*p < 0.05; **p < 0.01; ***p < 0.001; ns, not significant).

To analyse the composition of immune cells in different risk groups, we used the Wilcoxon test to compare the distribution of immune cells. As shown in Figure 5A, we found that CD8 T cells, Tregs regulatory T cells, M0 macrophages, and resting natural killer (NK) cells were more abundant in the high-risk group, while plasma cells, resting memory CD4 T cells, activated memory CD4 T cells and M2 macrophages were more abundant in the low-risk group. Likewise, activated memory CD4 T cells and M2 macrophages are significantly distributed in different stages of CRC patients (Supplementary Figure S9). The correlations between the m6A-related signature model and immunotherapeutic biomarkers were then investigated. Compared with that in the low-risk group, PD1 and CTLA4 expression in the high-risk was significantly higher, suggesting that high-risk CRC patients have a potential response to anti-PD-1 immunotherapy (Figures 5B–D). Additionally, higher TIDE prediction score represented a higher potential for immune evasion, which suggested that the patients were less likely to benefit from (ICB) therapy. In our results, the low-risk group had a lower TIDE score than the high-risk group, implying that low-risk patients may have a better immunotherapy response than high-risk patients. Also, we found that the high-risk group had a higher microsatellite instability (MSI) score, T-cell exclusion score and dysfunction score (Figures 5E–H). To find the potency of m6A-related prognostic score as a biomarker for predicting the response of CRC patients to drugs, “pRRophetic” algorithm was used to infer the therapeutic response based on the IC50 value of the 138 anti-cancer drugs in TCGA-COAD/READ patients. We found 50 chemotherapeutic drugs displaying differential IC50 between these two groups (Supplementary Figure S10).

### Construction of nomogram based on prognostic risk score and clinical characteristics

**FIGURE 6:**
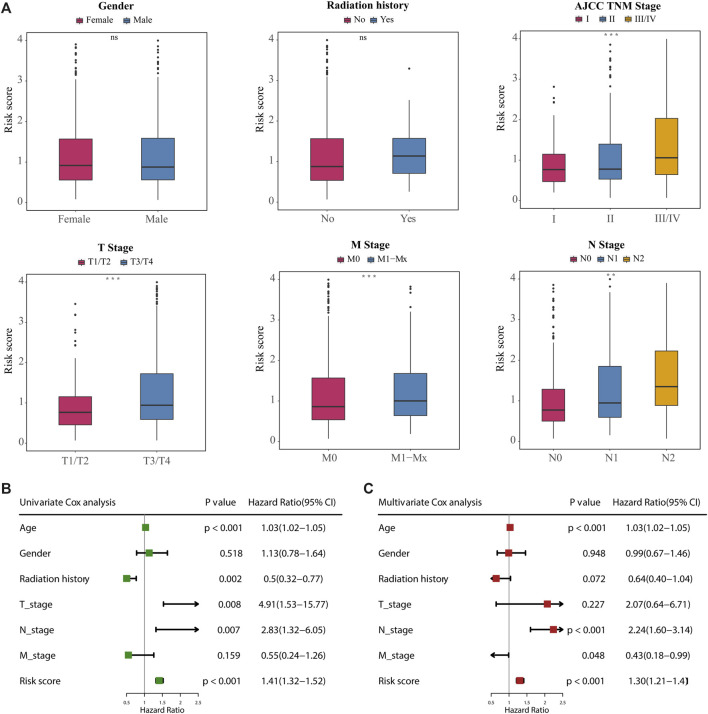
Correlation between the 18-gene expression signatures and clinical characteristics. **(A)** Difference analysis of the distribution of risk scores in different T, N, M, AJCC TNM stages, gender, and radiation history. Statistical difference of two groups was compared by the Wilcoxon test and three or more groups were compared by the Kruskal–Wallis test (*p < 0.05; **p < 0.01; ***p < 0.001; ns not significant). **(B, C)** Univariate **(B)** and multivariate **(C)** Cox regression analyses of correlations between the 18-gene expression signatures and clinical characteristics with OS, and revealed that the risk score based on the m6A-related gene expression signatures was an independent prognostic predictor in the TCGA dataset.

**FIGURE 7:**
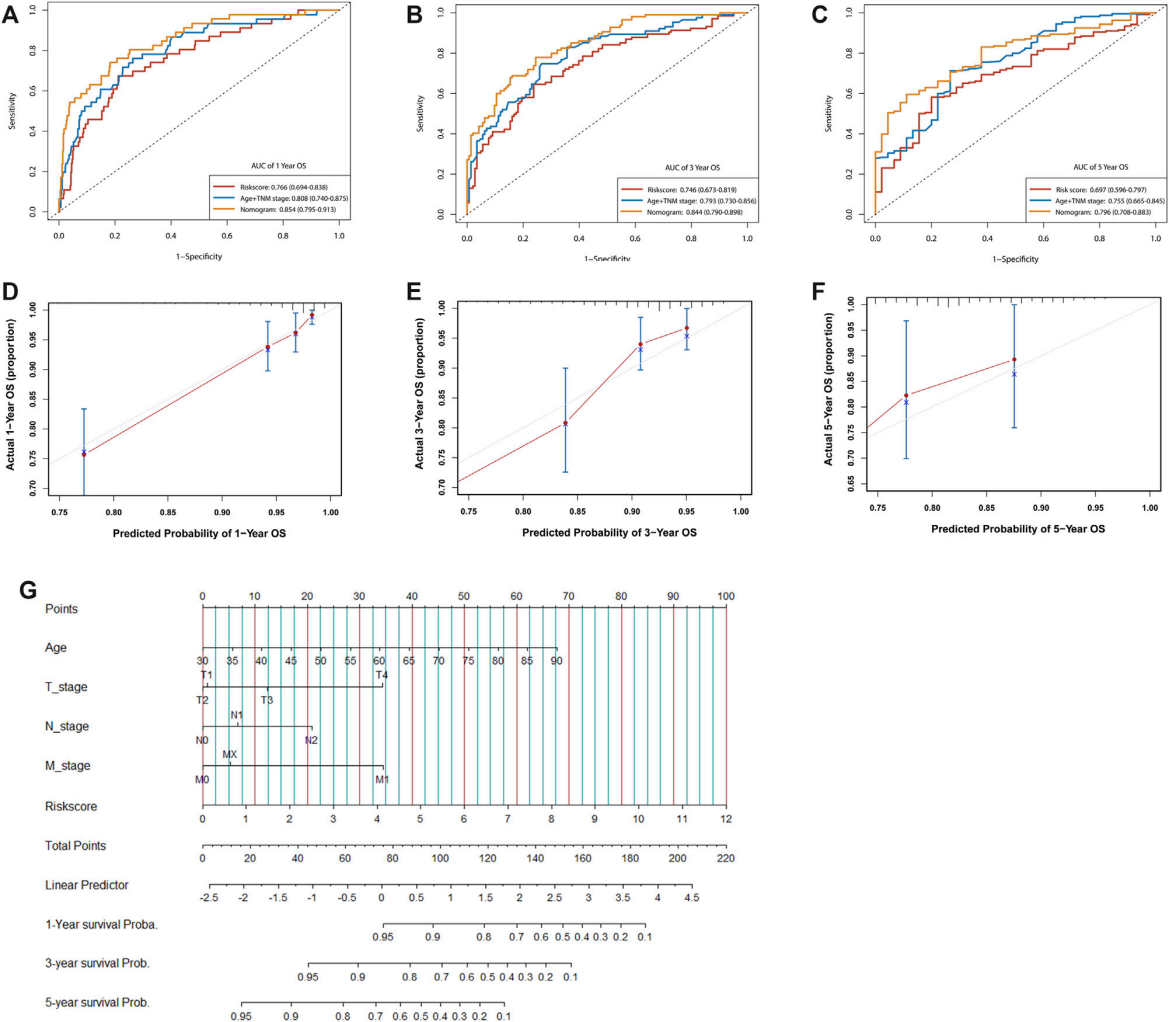
Assessment of the prognostic risk model of the m6A-related gene expression signatures and clinical features in CRC. **(A–C)** Time-dependent receiver operating characteristic (ROC) curves for the nomogram, risk score, and clinical characteristics in the TCGA dataset on predicting 1- **(A)**, 3- **(B)**, and 5-year **(C)** OS. **(D–F)** The calibration plot of the nomogram predicts the probability of the 1- **(D)**, 3- **(E)**, and 5- **(F)** year OS. **(G)** Nomogram for predicting the 1-, 3-, and 5-year OS of patients with CRC.

We next investigated the distribution of the risk score of patients with CRC using different conventional clinical information (including sex, T stage, N stage, M stage and AJCC TNM stage), and confirmed that CRC patients with higher T, N or TNM stage had a higher risk score (Figure 6A). Univariate Cox analysis showed that age, radiation history, T stage, N stage and the prognostic risk score were significantly associated with the prognosis of CRC (Figure 6B). Multivariate Cox analysis confirmed that the prognostic risk score based on m6A-related gene expression signatures was an independent predictor of CRC survival (Figure 6C). Multivariate Cox prediction models combing prognostic risk score and clinical characteristics yielded AUCs of 0.854 (95%CI: 0.795–0.913), 0.844 (95%CI: 0.790–0.898) and 0.796 (95%CI: 0.708–0.883) for the prediction of the 1-, 3-, 5-year OS (Figures 7A–C), which displayed superior predictive performance over the model that only included clinical characteristics with AUCs of 0.808 (95%CI: 0.740–0.875), 0.793 (95%CI: 0.730–0.856) and 0.755 (95%CI: 0.665–0.845). Calibration plots showed that the observed vs predicted rates of 1-, 3-, 5-year OS had good concordance (Figures 7D–F). Accordingly, based on the risk score and clinical characteristics, a prognostic nomogram was established for the prediction of OS in CRC patients as shown in Figure 7G. The validation results (Supplementary Figure S11) were consistent with the findings in TCGA training set, which indicated that m6A-based model had a stable OS-predictive ability.

## Discussion

Here, we developed a prognostic risk score based on m6A-related gene expression signatures and performed external validation to assess its prediction accuracy. Our study indicated that the m6A-based prognostic risk score was an independent predictor for CRC survival and had improved the prediction accuracy of CRC survival when combined with clinical characteristics. When stratified by this risk score, a worse survival rate, lower immunogenicity, and greater number of somatic mutations were shown in the high-risk group. The low-risk group had a lower TIDE score than the high-risk group for predicting immunotherapy response, implying that low-risk patients could benefit more from immunotherapy than high-risk patients.

Evidence from numerous studies have been discovered that RNA modifications regulate most steps of the gene expression, from DNA transcription to RNA translation (Helm and Motorin, 2017; Delaunay and Frye, 2019), through the effect of CNV and mutations to m6A regulators, including the alterations of RBM15, YTHDF2, YTHDC1, YTHDC2, and METTL14 (Zhang Q. et al., 2020). Besides, recent studies suggest that m6A modification plays important roles in RNA metabolism and cell proliferation, with significant implications on a variety of cell-physiological processes and cancer development (Zhang C. et al., 2020; Tian et al., 2020). In our study, functional enrichment analyses indicated that *CDK5R4P2*, *CLK1*, *CNOT3*, *GPR125*, *ING5*, *SFPQ* and *UBE2H* are mainly involved in the neural, destabilization and metabolic processes of mRNA signatures, and influence the growth, differentiation and communications of multiple colon cell types. Interestingly, *GPR125* and *SFPQ* were enriched in a neural signaling pathway in relation to Spinocerebellar ataxia. Additionally, *CLK1,* a novel CLK kinases inhibitor, has been reported to impair the growth of CRC cell lines and organoids, and inhibit anchorage-independent colony formation and cell migration, thus promoting cytotoxicity (Sohail et al., 2021). *UBE2H* belongs to the ubiquitin-conjugating enzyme (*UBE2*) family, and there are several studies investing the role of UBE2 family in carcinogenesis, especially malignant breast cancer (Ayesha et al., 2016) and lung cancer (Jiang et al., 2017; Liu and Xu, 2018). Evidence from the existing research suggested that *MET-UBE2H* might be a novel prognostic biomarker or target in lung adenocarcinoma (Zhu et al., 2018). And *UBE2H* was also identified as an m6A-related hub gene closely related to the clinicopathology and prognosis of CRC using a prognostic signature model (Zhang and Zhang, 2021). In concordance with our findings, Cejas et al also found that *CNOT3* overexpression in colon tissues was associated with worse prognosis outcomes of CRC (Cejas et al., 2017).

Our study firstly developed a prognostic risk score based on 18 m6A-related gene expression signatures that could be used as an index to predict the OS of CRC patients, and further validated its predictive performance in two independent external datasets. The risk stratification analysis showed that the m6A-based prognostic risk score had a good prognostic accuracy in predicting the OS in both the TCGA and validation datasets. Time-dependent AUC also confirmed that combination of the m6A-related prognostic risk score with clinical predictors (TNM stage and age) displayed superior predictive performance over the model that only included clinical characteristics of OS for CRC patients. Stratified analysis also confirmed that the risk score could predict CRC survival with good performance in different clinical subgroups (age, T stage, AJCC TNM stage). Taken together, this m6A-based prognostic risk score could be used as an independent predictor for CRC survival and the application of risk score in combination with clinical characteristics could improve the prediction accuracy of CRC survival.

Using this m6A-related prognostic risk score as a classifier, CRC patients were stratified into low- and high-risk groups to gain further biological insight into the gene mutations and immunologic nature of CRC patients in different risk groups. We found that m6A-related gene expression signatures were differentially enriched in the pathways related to cancer, immune response, and neural signaling between the two groups. When examining the somatic mutations, we found that the top 20 cancer driver genes mutated more frequently in the high-risk group than in the low-risk group, and significant co-occurrences were also observed among mutations of these genes. By examining the immunologic nature of CRC patients in different risk groups, we found high-risk group generally had higher monocytes and macrophages M1 infiltration and fractions of T cells CD8, and lower memory resting CD4 T cells than low-risk patients. Evidence from experimental studies observed that the infiltration levels of CD8^+^T increased in YTHDF1-deficient mouse tumour, thereby enhancing an elevated antigen-specific CD8^+^T cell antitumor response *in vivo* (Han et al., 2019). Additionally, it has been reported that CRC patients enriched with M1 phenotype and the high islet density of M1 macrophages would have poor prognosis (Zhang et al., 2012), as well as M0 macrophages (Zhang et al., 2021). And researches have revealed that the strategies converting M2 macrophages to M1 macrophages of tumour associated macrophages (TAMs) suppressed tumour growth (Dong et al., 2020). Of note, another study of human CRC specimens illustrated that those with high densities of CD4^+^T were associated with a lower likelihood of tumour relapse and improved OS (Galon et al., 2006), which are consistent with the findings from our study. These indicate that the m6A-related gene expression signatures may modulate the TME phenotypes to influence the survival of CRC patients.

Emerging pieces of evidence showed that different TME phenotypes might have different degrees of benefit from immunotherapeutic treatment (Wang et al., 2019). It is reported that less immunogenic cancer cells are selected for during tumour development in immune-competent hosts to evade antitumor immune responses (Dunn et al., 2002), which may result in increased immunosuppressive cells (e.g., regulatory T cells and TAMs) and expression of immunosuppressive molecules (e.g., CTLA4 and PD1). As expected, we found that CTLA4 and PD1 expression levels was significantly higher in high-risk CRC patients. A Tumour Immune Dysfunction and Exclusion (TIDE) score has been increasingly used as an index for predicting immunotherapeutic response (Jiang et al., 2018). Consistently, using the TIDE algorithm, we estimated the immune response and found that patients in the low-risk group have a superior response to immunotherapy. Chemotherapy results indicated that the high-risk patients with CRC were more sensitive to 24 chemotherapies than low-risk patients. These results suggested that the poorer prognosis for high-risk patients could be due to higher immunosuppression in the TME, and that TME may influence the response of chemotherapy and immunotherapy. Based on these findings, this m6A-based risk score might also be used as an indicator for predicting immunotherapy response among CRC patients.

Our study also provides insight into the process and mechanism of m6A modification of gene expression signatures for future studies. However, we are also aware of several limitations in this study. Although the m6A-related gene signatures prognostic risk score showed superior performance on the prediction of CRC survival and the response to immunotherapy, it should be prospectively validated in real clinical settings and the clinicopathological factors should also be considered. Moreover, distribution of immune cells in this m6A-based classifier (e.g., T cells and macrophages infiltration) could be influenced by the difference in research datasets, sequencing method and sample size, and both the TIDE and MSI scores focused on the function and status of T cells, which could not fully reflect the complexity of the TME involved in the immunotherapeutic response. Thus, further observational and experimental studies should be performed to elucidate the accuracy of this prognostic risk score in the prediction of CRC survival, and to understand how targeted immunotherapy against m6A regulators could be applied in the clinic to achieve much improved cancer therapy in the future.

In conclusion, we developed a prognostic risk score based on the expression signature of 18 genes associated with m6A modification to predict the OS of CRC patients and their response to immunotherapy. This work highlights the clinical implications of this risk score in distinguishing immune and molecular characteristics and identifying response of target treatments. The derived m6A-related risk score showed the potential to be used as a prognostic and therapeutic indicator for the prediction of CRC prognosis and the development of individualized CRC treatment strategy.

## Data Availability

The datasets presented in this study can be found in online repositories. The names of the repository/repositories and accession number(s) can be found in the article/Supplementary Material.
