# Supplementary material for: N6-methyladenosine related gene expression signatures for predicting the overall survival and immune responses of patients with colorectal cancer
Source: Front Genet. 2023 Mar 3;14:885930. doi: 10.3389/fgene.2023.885930 (PMC10020527; doi:10.3389/fgene.2023.885930)
Supplement: Supplementary file 1 [file Table1.docx]

**N6-methylandenosine-related gene expression signatures for predicting the overall survival and immune responses of patients with colorectal cancer**

Supplementary figures

**Figure S1. Landscape of expression variation of m6A regulators in colorectal cancer.**

**Figure S2. Lasso Cox regression analysis of m6A-related gene signatures.**

**Figure S3. Heatmap of the correlations between m6A regulators and the prognostic m6A-related gene expression signatures (*p < 0.05; **p < 0.01; and ***p < 0.001).**

**Figure S4. Protein expression levels of m6A regulators (A) and m6A-related gene signatures (B) (HPA database).**

**Figure S5. Principal component analysis between the high- and low-risk subgroups.**

**Figure S6. Kaplan-Meier plot of 18 m6A-related gene expression signatures in CRC patients from TCGA database in different expression levels.**

**Figure S7. Kaplan-Meier curves of OS differences stratified by sex** (**A and B**)**, age (C and D), tumor grade (E and F), or T stage (G and H) between the high- and low-risk groups.**

**Figure S8. KEGG enrichment analysis identified 30 significantly pathways enriched by the m6A-related gene expression signatures and their co-expression genes.**

**Figure S9 The proportions of TME cells in different stage subgroups in CRC patients. Significant statistical differences between the two subgroups were assessed using the Wilcoxon test, the asterisks represented the statistical p value (ns, not significant; *P < 0.05; **P < 0.01; ***P < 0.001; ****P < 0.0001).**

**Figure S10. Chemotherapeutic responses in high- and low-risk patients with CRC.**

**Figure S11. ROC curves of m6A-related based risk score and clinical characteristics for predicting 1- (A), 3- (B), and 5-year (C) OS in the validation GSE39582 dataset, and 1- (D), 3- (E), and 5-year (F) OS in the validation GSE17536 dataset.**

**Figure S1**

**
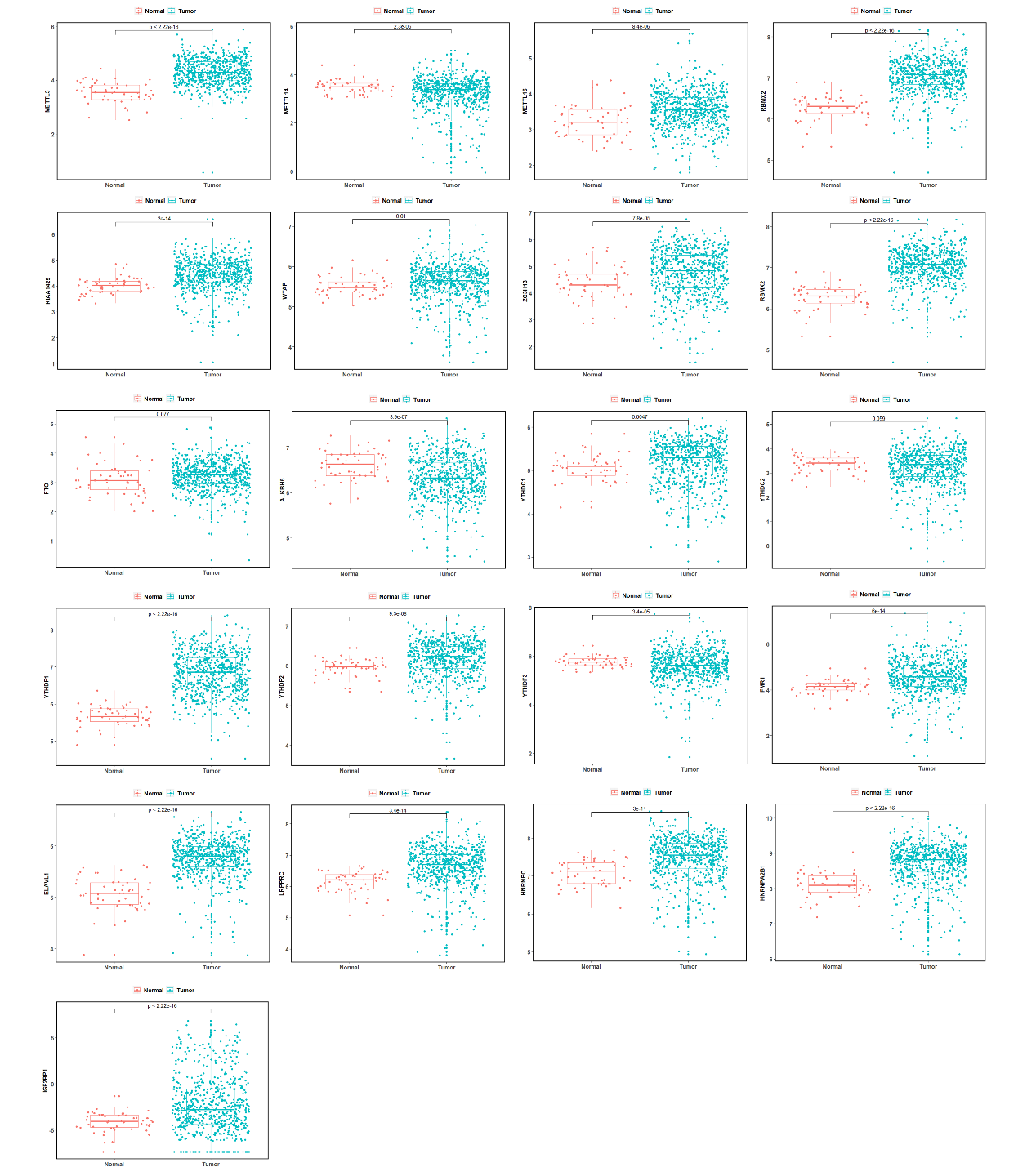
**

**Figure S1. Landscape of expression variation of m6A regulators in colorectal cancer.**

Expression differences of 21 m6A regulators between normal tissues and CRC tissues.

**Figure S2**


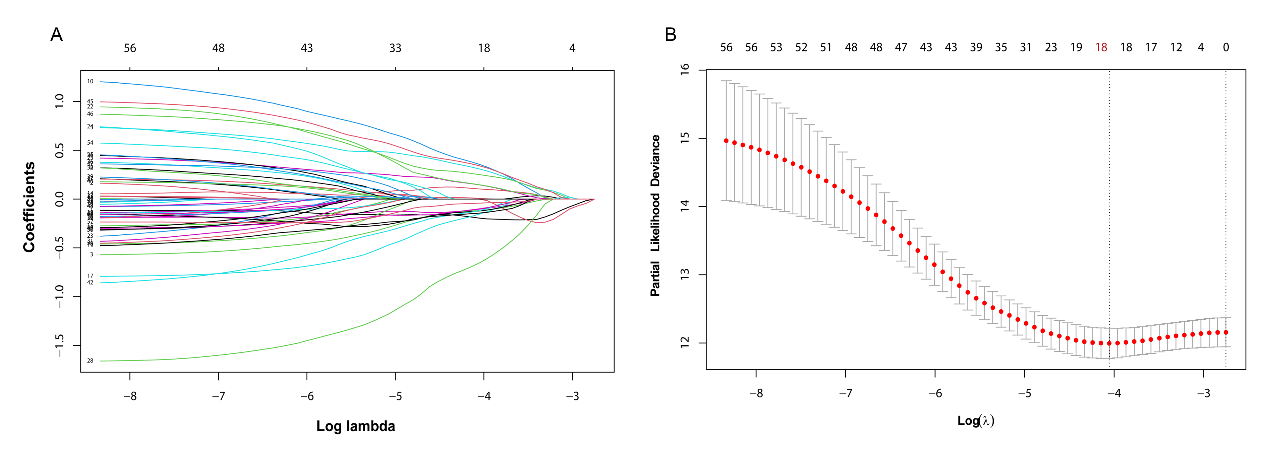


**Figure S2. Lasso Cox regression analysis of m6A-related gene signatures.** (**A**)The LASSO coefficient profile of 57 OS-related genes and perpendicular imaginary lines were drawn at the value chosen by 10-fold cross-validation. (**B**) The tuning parameters (log λ) of OS-related genes were selected to cross-verify the error curve. According to the minimal criterion and 1-se criterion, perpendicular imaginary lines were drawn at the optimal value.

**Figure S3**

**
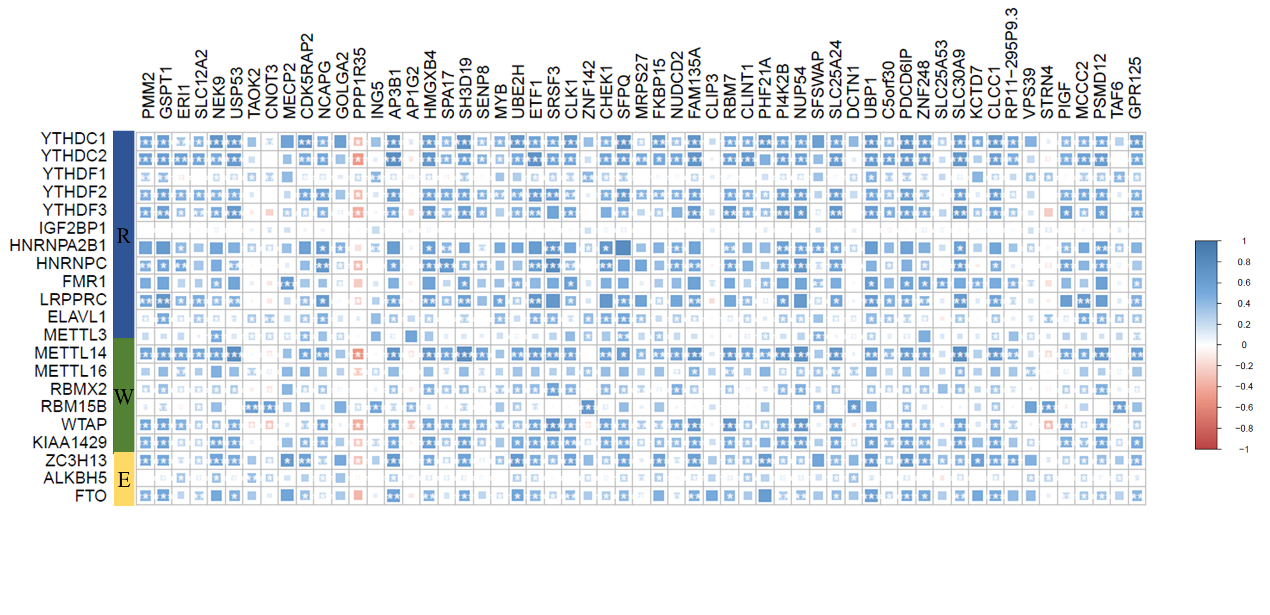
**

**Figure S3. Heatmap of the correlations between m6A regulators and the prognostic m6A-related gene expression signatures (*p < 0.05; **p < 0.01; and ***p < 0.001).**

**Figure S4**


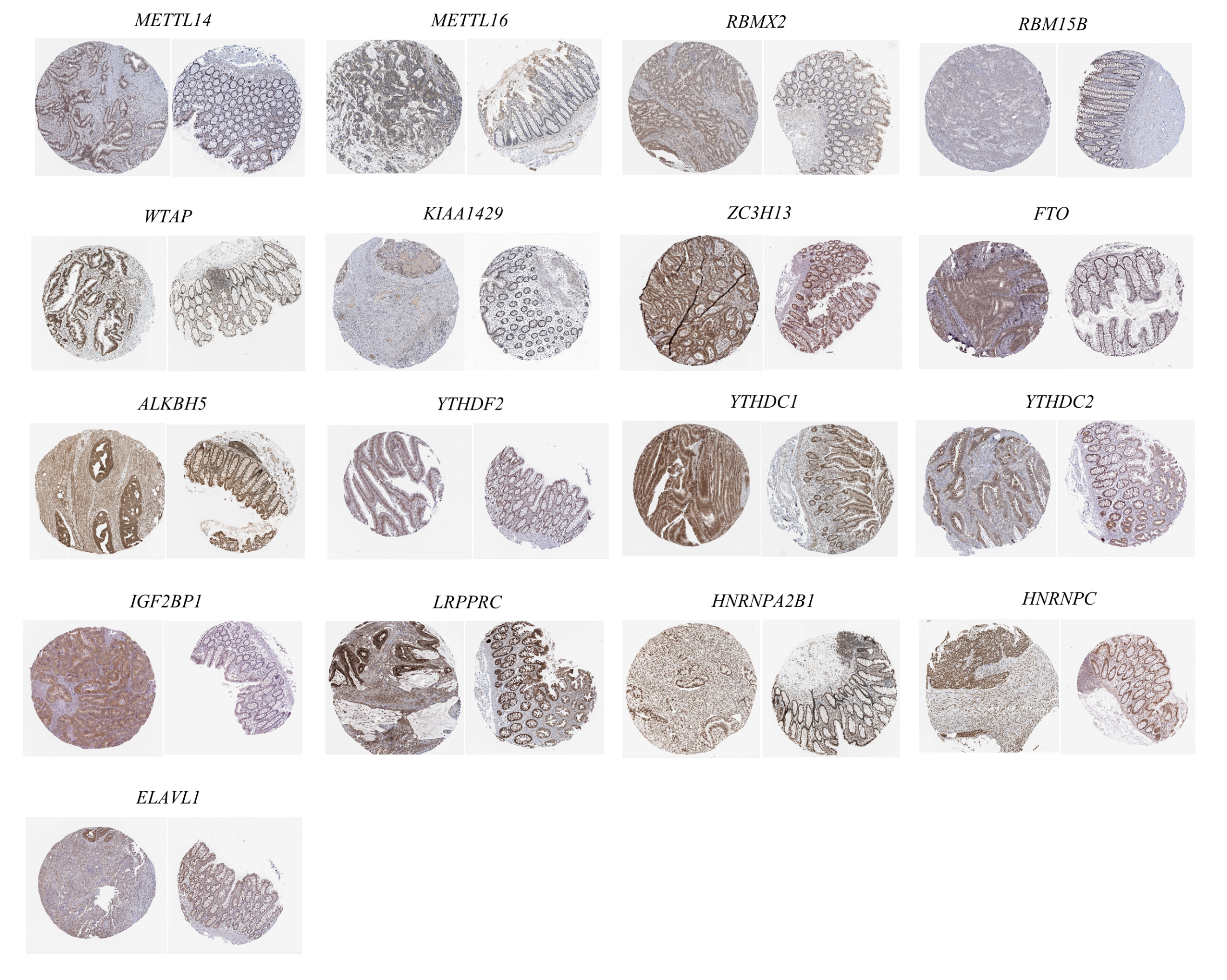


**
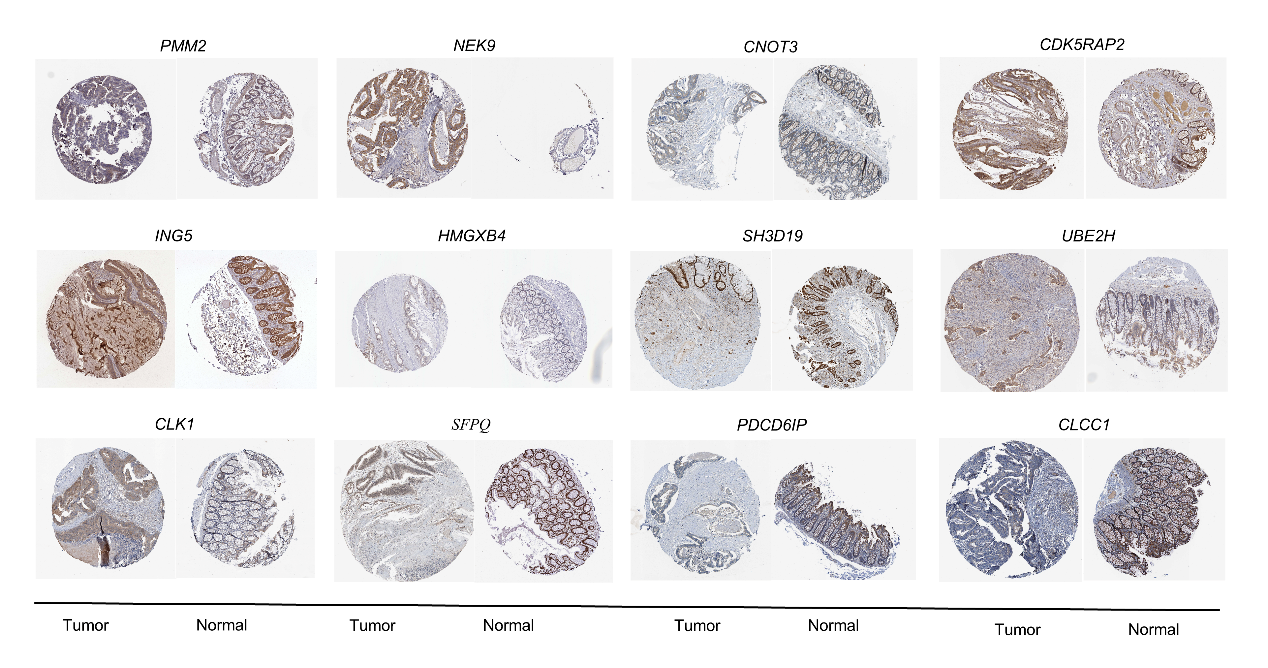
**

A

B

**Figure S4.** **Protein expression levels of m6A regulators (A) and m6A-related gene signatures (B) (HPA database).**

**Figure S5**

**
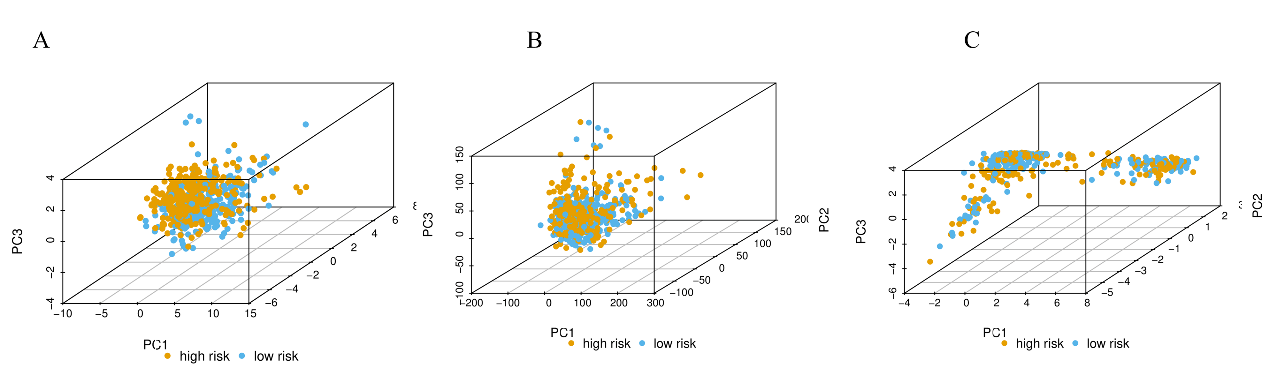
**

**Figure S5. Principal component analysis between the high- and low-risk subgroups.** (**A**) entire gene expression profiles, (**B**) 21 m6A regulators and (**C**)the expression profile of the 18 m6A-related gene expression signatures.

**Figure S
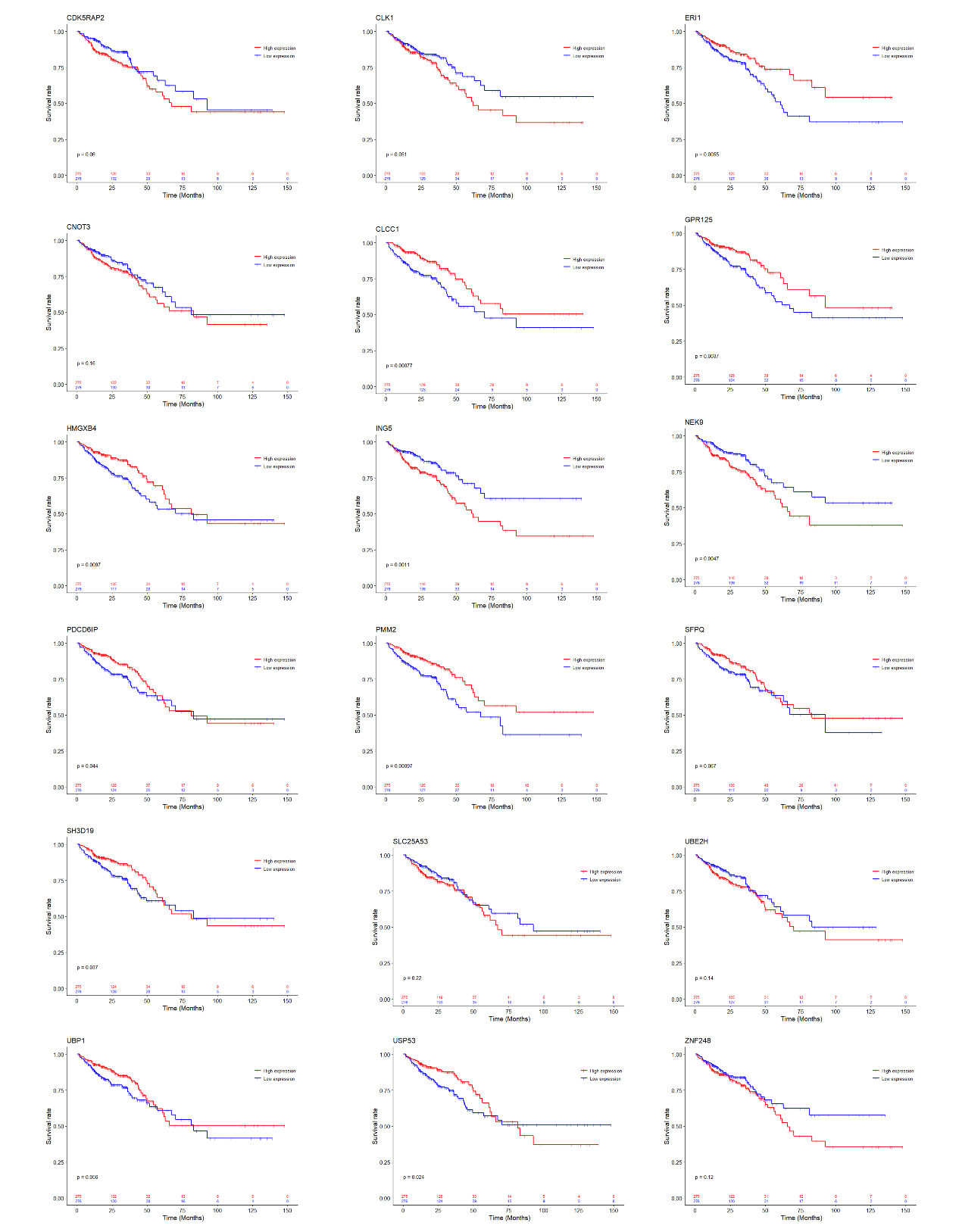
6**

**Figure S6. Kaplan-Meier plot of 18 m6A-related gene expression signatures in CRC patients from TCGA database in different expression levels.**

**Figure S7**


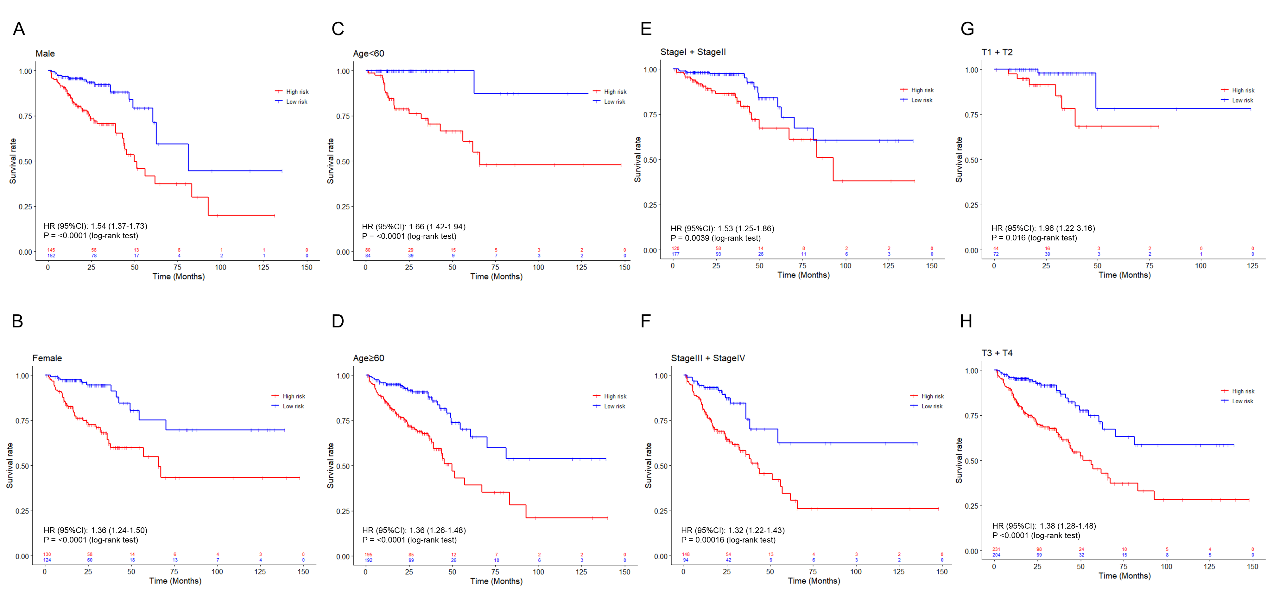


**Figure S7. Kaplan-Meier curves of OS differences stratified by sex** (**A and B**)**, age (C and D), tumor grade (E and F), or T stage (G and H) between the high- and low-risk groups.**

**Figure S8**

**
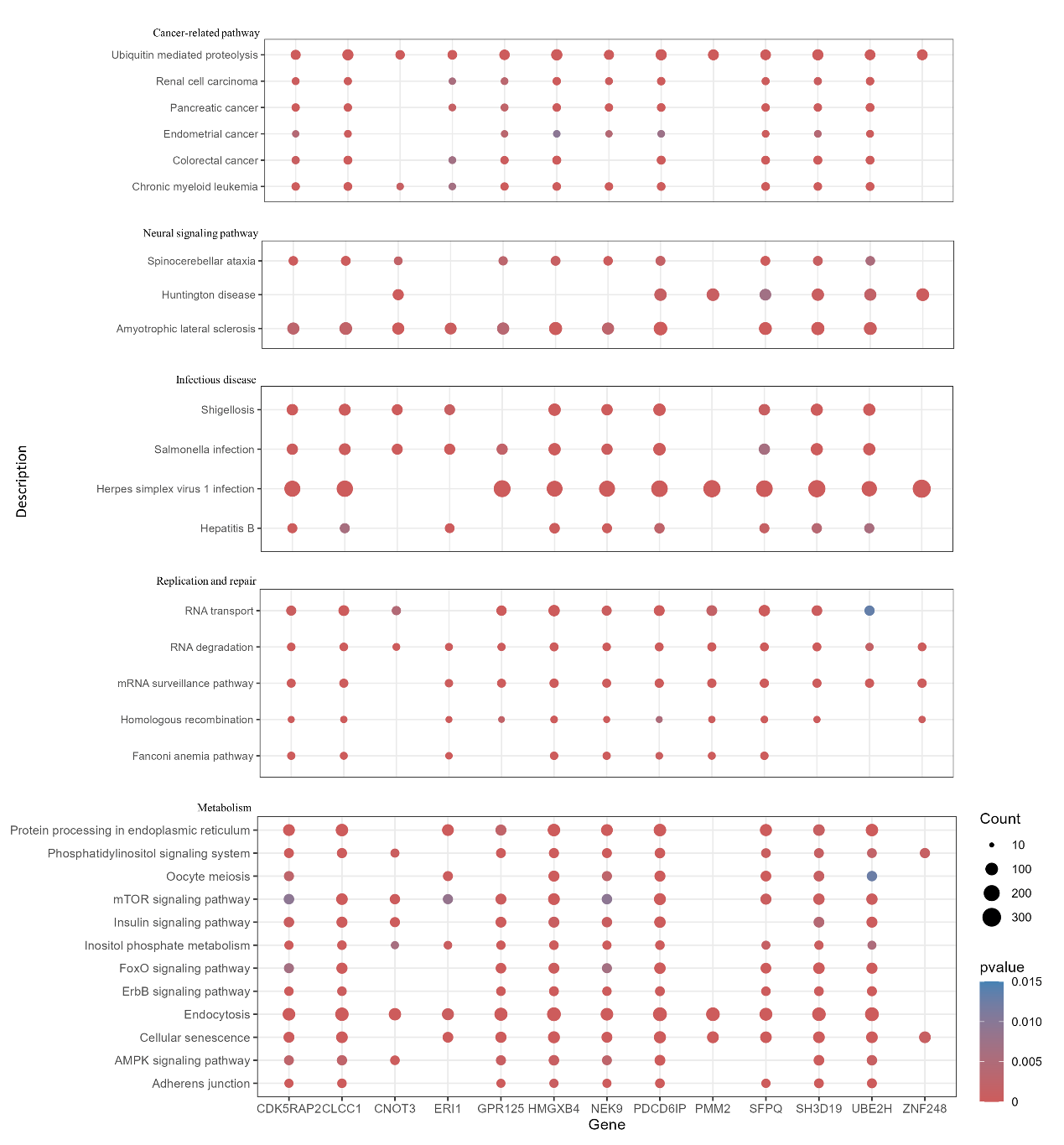
**

**Figure S8. KEGG enrichment analysis identified 30 significantly pathways enriched by the m6A-related gene expression signatures and their co-expression genes.**

**Figure S9**

**
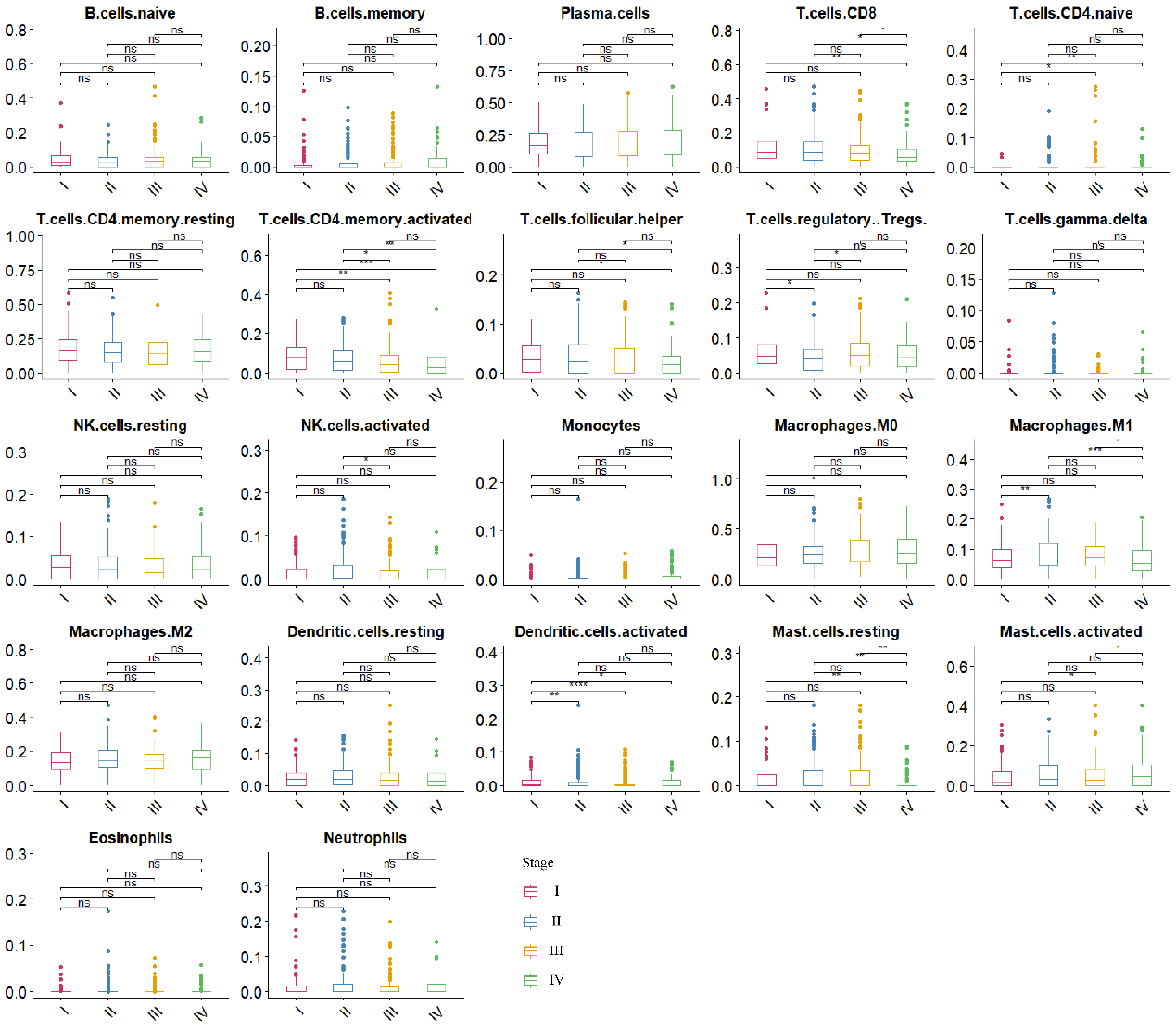
 Figure S9 The proportions of TME cells in different stage subgroups. Significant statistical differences between the two subgroups were assessed using the Wilcoxon test, the asterisks represented the statistical p value (ns, not significant; *P < 0.05; **P < 0.01; ***P < 0.001; ****P < 0.0001).**

**Figure S10**

**
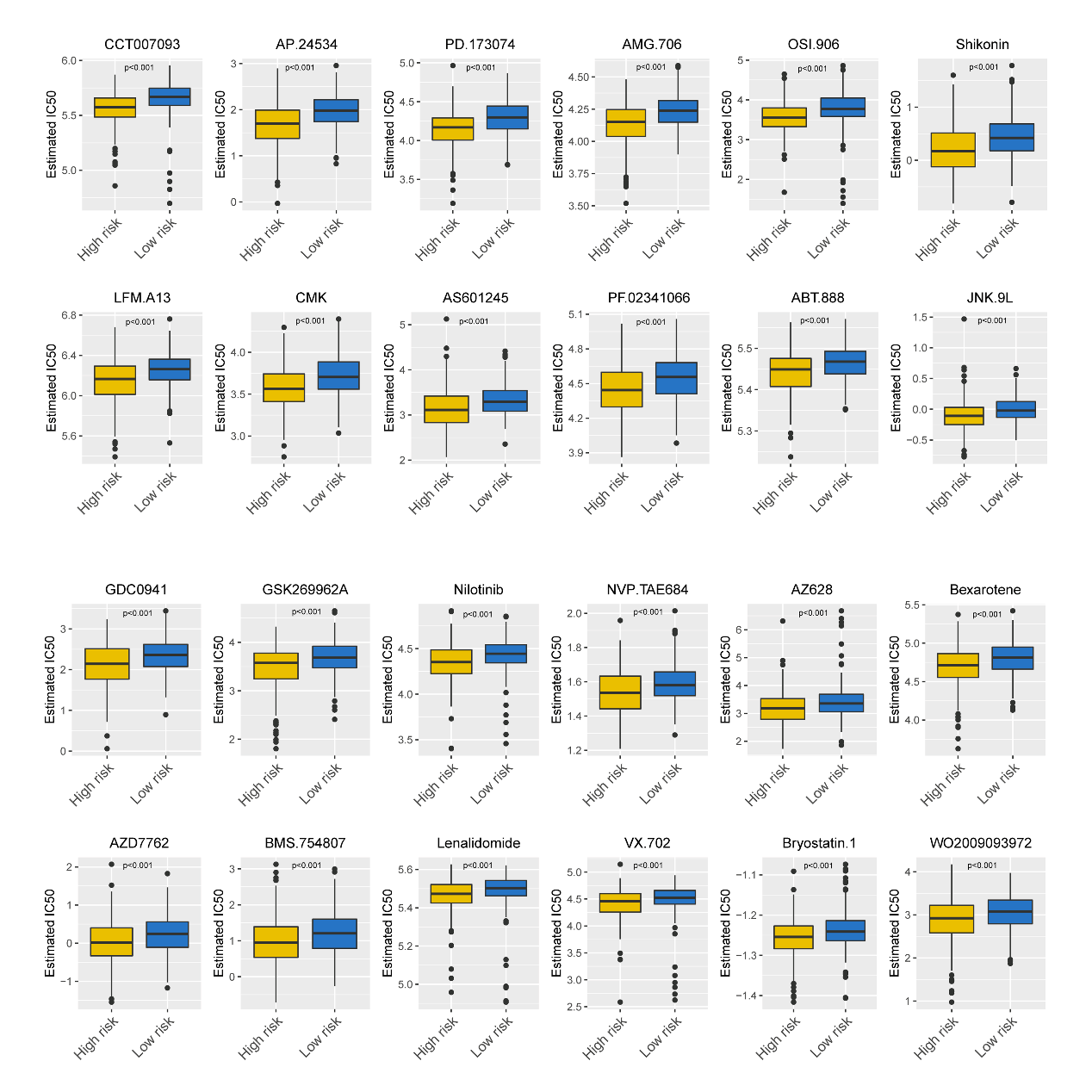
**

**Figure S10. Chemotherapeutic responses in high- and low-risk patients with CRC.**

Differential chemotherapeutic responses in high- and low-risk patients.

**Figure S11**

**
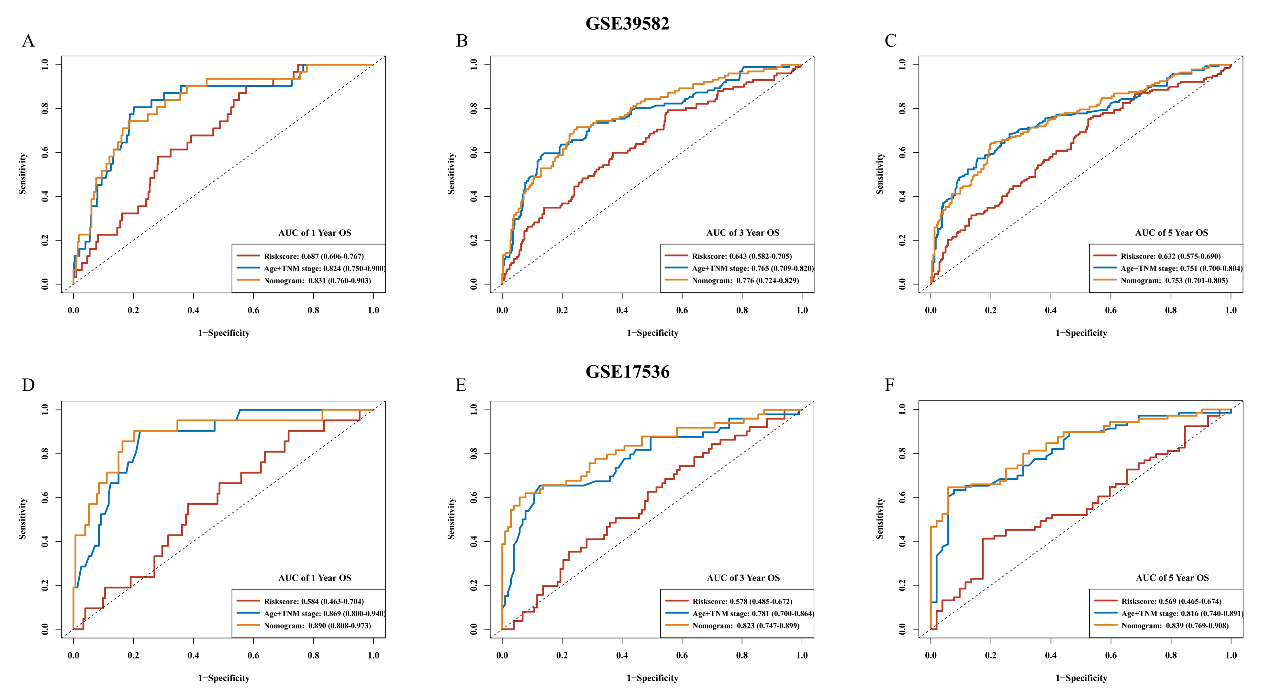
**

**Figure S11. ROC curves of m6A-related based risk score and clinical characteristics for predicting** 1**- (A), 3- (B), and 5-year (C) OS in the validation GSE39582 dataset, and** 1**- (D), 3- (E), and 5-year (F) OS in the validation GSE17536 dataset.**
