# Supplementary material for: N6-methyladenosine related gene expression signatures for predicting the overall survival and immune responses of patients with colorectal cancer
Source: Front Genet. 2023 Mar 3;14:885930. doi: 10.3389/fgene.2023.885930 (PMC10020527; doi:10.3389/fgene.2023.885930)
Supplement: Supplementary file 2 [file Table2.docx]

**N6-methylandenosine-related gene expression signatures for predicting the overall survival and immune responses of patients with colorectal cancer**

Supplementary tables

**Supplementary Table S1: Univariate Cox proportional hazard regression results for significant m6A-related genes (FDR<0.05).**

**Supplementary Table S2: KEGG pathway enrichment results of co-expressed genes with identified m6A-related genes in COAD/READ tissue.**

| **Supplementary Table S1. Univariate Cox proportional hazard regression results for significant m6A-related genes (FDR<0.05).** | | | | |
| --- | --- | --- | --- | --- |
| **Gene** | **Coef** | **HR (95% CI for HR)** | ***P* value** | **FDR** |
| *PMM2* | -0.553 | 0.58 (0.42-0.79) | 5.27E-04 | 3.11E-03 |
| *GSPT1* | -0.535 | 0.59 (0.43-0.80) | 7.95E-04 | 4.04E-03 |
| *ERI1* | -0.318 | 0.73 (0.57-0.93) | 1.21E-02 | 1.93E-02 |
| *SLC12A2* | -0.219 | 0.80 (0.67-0.96) | 1.91E-02 | 2.17E-02 |
| *NEK9* | 0.416 | 1.52 (1.07-2.14) | 1.90E-02 | 2.35E-02 |
| *USP53* | -0.275 | 0.76 (0.60-0.96) | 1.89E-02 | 2.64E-02 |
| *TAOK2* | 0.560 | 1.75 (1.13-2.72) | 1.26E-02 | 2.99E-02 |
| *CNOT3* | 0.472 | 1.60 (1.05-2.46) | 3.00E-02 | 3.00E-02 |
| *MECP2* | 0.397 | 1.49 (1.04-2.13) | 2.97E-02 | 3.08E-02 |
| *CDK5RAP2* | 0.412 | 1.51 (1.04-2.19) | 3.00E-02 | 3.09E-02 |
| *NCAPG* | -0.269 | 0.76 (0.60-0.97) | 2.90E-02 | 3.10E-02 |
| *GOLGA2* | 0.405 | 1.50 (1.04-2.16) | 2.97E-02 | 3.10E-02 |
| *PPP1R35* | 0.281 | 1.32 (1.03-1.71) | 3.09E-02 | 3.11E-02 |
| *ING5* | 0.419 | 1.52 (1.04-2.22) | 3.08E-02 | 3.13E-02 |
| *AP3B1* | -0.335 | 0.72 (0.54-0.95) | 1.86E-02 | 3.15E-02 |
| *AP1G2* | 0.337 | 1.40 (1.03-1.90) | 3.13E-02 | 3.16E-02 |
| *HMGXB4* | -0.404 | 0.67 (0.46-0.96) | 2.95E-02 | 3.18E-02 |
| *SPA17* | -0.317 | 0.73 (0.55-0.97) | 3.06E-02 | 3.19E-02 |
| *SH3D19* | -0.261 | 0.77 (0.61-0.97) | 2.93E-02 | 3.19E-02 |
| *SENP8* | -0.484 | 0.62 (0.40-0.95) | 2.89E-02 | 3.22E-02 |
| *MYB* | -0.221 | 0.80 (0.65-0.98) | 3.16E-02 | 3.23E-02 |
| *UBE2H* | 0.441 | 1.55 (1.08-2.23) | 1.69E-02 | 3.33E-02 |
| *ETF1* | -0.406 | 0.67 (0.47-0.94) | 1.89E-02 | 3.38E-02 |
| *SRSF3* | -0.382 | 0.68 (0.48-0.97) | 3.21E-02 | 3.40E-02 |
| *CLK1* | 0.294 | 1.34 (1.03-1.76) | 3.23E-02 | 3.40E-02 |
| *ZNF142* | 0.451 | 1.57 (1.05-2.35) | 2.89E-02 | 3.47E-02 |
| *CHEK1* | -0.301 | 0.74 (0.56-0.98) | 3.37E-02 | 3.59E-02 |
| *SFPQ* | -0.557 | 0.57 (0.37-0.89) | 1.21E-02 | 3.63E-02 |
| *MRPS27* | -0.369 | 0.69 (0.49-0.97) | 3.37E-02 | 3.70E-02 |
| *FKBP15* | 0.479 | 1.61 (1.04-2.51) | 3.35E-02 | 3.71E-02 |
| *NUDCD2* | -0.400 | 0.67 (0.46-0.97) | 3.31E-02 | 3.74E-02 |
| *FAM135A* | -0.251 | 0.78 (0.62-0.98) | 3.35E-02 | 3.85E-02 |
| *CLIP3* | 0.206 | 1.23 (1.02-1.48) | 2.88E-02 | 3.85E-02 |
| *RBM7* | -0.325 | 0.72 (0.54-0.97) | 2.80E-02 | 3.98E-02 |
| *CLINT1* | -0.362 | 0.70 (0.51-0.94) | 2.00E-02 | 4.03E-02 |
| *PHF21A* | 0.464 | 1.59 (1.11-2.28) | 1.19E-02 | 4.10E-02 |
| *PI4K2B* | -0.293 | 0.75 (0.59-0.94) | 1.35E-02 | 4.20E-02 |
| *NUP54* | -0.293 | 0.75 (0.57-0.98) | 3.55E-02 | 4.30E-02 |
| *SFSWAP* | 0.506 | 1.66 (1.08-2.55) | 2.06E-02 | 4.33E-02 |
| *SLC25A24* | -0.261 | 0.77 (0.62-0.95) | 1.68E-02 | 4.46E-02 |
| *DCTN1* | 0.507 | 1.66 (1.08-2.55) | 2.04E-02 | 4.50E-02 |
| *UBP1* | -0.347 | 0.71 (0.51-0.98) | 3.96E-02 | 4.52E-02 |
| *C5orf30* | -0.325 | 0.72 (0.56-0.94) | 1.39E-02 | 4.53E-02 |
| *PDCD6IP* | -0.384 | 0.68 (0.50-0.94) | 1.85E-02 | 4.57E-02 |
| *ZNF248* | 0.354 | 1.42 (1.03-1.98) | 3.49E-02 | 4.57E-02 |
| *SLC25A53* | 0.554 | 1.74 (1.03-2.94) | 3.88E-02 | 4.59E-02 |
| *SLC30A9* | -0.282 | 0.75 (0.58-0.98) | 3.59E-02 | 4.60E-02 |
| *KCTD7* | 0.356 | 1.43 (1.07-1.91) | 1.63E-02 | 4.67E-02 |
| *CLCC1* | -0.390 | 0.68 (0.48-0.96) | 2.69E-02 | 4.69E-02 |
| *RP11-295P9.3* | 0.264 | 1.30 (1.01-1.68) | 4.07E-02 | 4.78E-02 |
| *VPS39* | 0.471 | 1.60 (1.02-2.51) | 3.94E-02 | 4.81E-02 |
| *STRN4* | 0.453 | 1.57 (1.03-2.40) | 3.55E-02 | 4.81E-02 |
| *PIGF* | -0.418 | 0.66 (0.45-0.96) | 2.80E-02 | 4.83E-02 |
| *MCCC2* | -0.293 | 0.75 (0.56-0.99) | 4.05E-02 | 4.84E-02 |
| *PSMD12* | -0.439 | 0.64 (0.46-0.91) | 1.34E-02 | 4.90E-02 |
| *TAF6* | 0.449 | 1.57 (1.02-2.40) | 3.93E-02 | 4.92E-02 |
| *GPR125* | -0.349 | 0.71 (0.52-0.96) | 2.88E-02 | 4.93E-02 |

| **Supplementary Table S2. KEGG pathway enrichment results of co-expressed genes with identified m6A-related genes in COAD/READ tissue.** | | | | | | | |
| --- | --- | --- | --- | --- | --- | --- | --- |
| **Gene** | **Category** | **ID** | **Description** | **GeneRatio** | **BgRatio** | **pvalue** | **padjust** |
| *PMM2* | Cancer-related pathway | hsa04120 | Ubiquitin mediated proteolysis | 91/3059 | 140/8108 | 4.06E-11 | 6.68E-09 |
|  | Metabolism | hsa04110 | Cell cycle | 71/3059 | 124/8108 | 6.89E-06 | 5.67E-04 |
|  |  | hsa04144 | Endocytosis | 126/3059 | 252/8108 | 3.73E-05 | 2.04E-03 |
|  |  | hsa04218 | Cellular senescence | 83/3059 | 156/8108 | 5.22E-05 | 2.15E-03 |
|  | Infectious diseases | hsa05168 | Herpes simplex virus 1 infection | 306/3059 | 498/8108 | 1.69E-28 | 5.54E-26 |
|  | Neural signaling pathway | hsa05016 | Huntington disease | 142/3059 | 306/8108 | 9.67E-04 | 2.65E-02 |
|  | Other | hsa04140 | Autophagy - animal | 77/3059 | 141/8108 | 2.97E-05 | 1.95E-03 |
|  |  | hsa04740 | Olfactory transduction | 203/3059 | 443/8108 | 2.08E-04 | 6.83E-03 |
|  |  | hsa03015 | mRNA surveillance pathway | 56/3059 | 98/8108 | 6.81E-05 | 2.49E-03 |
|  |  | hsa03013 | RNA transport | 90/3059 | 186/8108 | 1.74E-03 | 4.40E-02 |
|  |  | hsa03440 | Homologous recombination | 27/3059 | 41/8108 | 2.35E-04 | 7.04E-03 |
|  |  | hsa03018 | RNA degradation | 52/3059 | 79/8108 | 3.47E-07 | 3.80E-05 |
|  |  | hsa03460 | Fanconi anemia pathway | 35/3059 | 54/8108 | 4.73E-05 | 2.15E-03 |
| *ERI1* | Cancer-related pathway | hsa03460 | Fanconi anemia pathway | 29/2011 | 54/8108 | 4.71E-06 | 3.11E-04 |
|  |  | hsa04115 | p53 signaling pathway | 34/2011 | 73/8108 | 4.09E-05 | 1.50E-03 |
|  |  | hsa04668 | TNF signaling pathway | 43/2011 | 112/8108 | 9.32E-04 | 1.14E-02 |
|  |  | hsa05212 | Pancreatic cancer | 31/2011 | 76/8108 | 1.50E-03 | 1.46E-02 |
|  |  | hsa05203 | Viral carcinogenesis | 69/2011 | 204/8108 | 2.15E-03 | 1.78E-02 |
|  |  | hsa05211 | Renal cell carcinoma | 27/2011 | 69/8108 | 5.81E-03 | 3.62E-02 |
|  |  | hsa05220 | Chronic myeloid leukemia | 29/2011 | 76/8108 | 6.58E-03 | 4.02E-02 |
|  |  | hsa05210 | Colorectal cancer | 32/2011 | 86/8108 | 6.88E-03 | 4.13E-02 |
|  |  | hsa04150 | mTOR signaling pathway | 52/2011 | 155/8108 | 8.47E-03 | 4.66E-02 |
|  | Neural signaling pathway | hsa04071 | Sphingolipid signaling pathway | 48/2011 | 119/8108 | 1.23E-04 | 3.13E-03 |
|  |  | hsa05014 | Amyotrophic lateral sclerosis | 118/2011 | 365/8108 | 5.53E-04 | 7.61E-03 |
|  |  | hsa05010 | Alzheimer disease | 114/2011 | 369/8108 | 3.92E-03 | 2.64E-02 |
|  |  | hsa05022 | Pathways of neurodegeneration - multiple diseases | 143/2011 | 476/8108 | 4.28E-03 | 2.77E-02 |
|  | Immune signaling pathway | hsa04660 | T cell receptor signaling pathway | 43/2011 | 104/8108 | 1.41E-04 | 3.24E-03 |
|  |  | hsa04625 | C-type lectin receptor signaling pathway | 40/2011 | 104/8108 | 1.33E-03 | 1.37E-02 |
|  |  | hsa05235 | PD-L1 expression and PD-1 checkpoint pathway in cancer | 34/2011 | 89/8108 | 3.36E-03 | 2.36E-02 |
|  |  | hsa04622 | RIG-I-like receptor signaling pathway | 28/2011 | 70/8108 | 3.48E-03 | 2.40E-02 |
|  |  | hsa04621 | NOD-like receptor signaling pathway | 62/2011 | 185/8108 | 4.44E-03 | 2.82E-02 |
|  |  | hsa04620 | Toll-like receptor signaling pathway | 37/2011 | 104/8108 | 8.87E-03 | 4.77E-02 |
|  | Metabolism | hsa04141 | Protein processing in endoplasmic reticulum | 81/2011 | 171/8108 | 8.82E-11 | 2.91E-08 |
|  |  | hsa04120 | Ubiquitin mediated proteolysis | 63/2011 | 140/8108 | 1.23E-07 | 2.03E-05 |
|  |  | hsa04110 | Cell cycle | 55/2011 | 124/8108 | 1.35E-06 | 1.49E-04 |
|  |  | hsa03440 | Homologous recombination | 23/2011 | 41/8108 | 1.77E-05 | 9.72E-04 |
|  |  | hsa04218 | Cellular senescence | 62/2011 | 156/8108 | 2.32E-05 | 1.09E-03 |
|  |  | hsa03018 | RNA degradation | 35/2011 | 79/8108 | 1.13E-04 | 3.12E-03 |
|  |  | hsa03015 | mRNA surveillance pathway | 41/2011 | 98/8108 | 1.47E-04 | 3.24E-03 |
|  |  | hsa03040 | Spliceosome | 56/2011 | 147/8108 | 2.17E-04 | 4.44E-03 |
|  |  | hsa04114 | Oocyte meiosis | 50/2011 | 129/8108 | 2.86E-04 | 5.25E-03 |
|  |  | hsa01240 | Biosynthesis of cofactors | 58/2011 | 156/8108 | 3.56E-04 | 5.87E-03 |
|  |  | hsa04142 | Lysosome | 49/2011 | 128/8108 | 4.60E-04 | 7.13E-03 |
|  |  | hsa04144 | Endocytosis | 86/2011 | 252/8108 | 4.75E-04 | 7.13E-03 |
|  |  | hsa00562 | Inositol phosphate metabolism | 31/2011 | 73/8108 | 6.74E-04 | 8.89E-03 |
|  |  | hsa03420 | Nucleotide excision repair | 22/2011 | 47/8108 | 8.39E-04 | 1.06E-02 |
|  |  | hsa04210 | Apoptosis | 50/2011 | 136/8108 | 1.18E-03 | 1.27E-02 |
|  |  | hsa00280 | Valine, leucine and isoleucine degradation | 22/2011 | 48/8108 | 1.19E-03 | 1.27E-02 |
|  |  | hsa04216 | Ferroptosis | 19/2011 | 41/8108 | 2.16E-03 | 1.78E-02 |
|  |  | hsa00020 | Citrate cycle (TCA cycle) | 15/2011 | 30/8108 | 2.48E-03 | 1.95E-02 |
|  |  | hsa00630 | Glyoxylate and dicarboxylate metabolism | 15/2011 | 30/8108 | 2.48E-03 | 1.95E-02 |
|  |  | hsa04130 | SNARE interactions in vesicular transport | 16/2011 | 33/8108 | 2.69E-03 | 2.02E-02 |
|  |  | hsa01212 | Fatty acid metabolism | 24/2011 | 57/8108 | 3.01E-03 | 2.21E-02 |
|  |  | hsa01200 | Carbon metabolism | 42/2011 | 115/8108 | 3.23E-03 | 2.32E-02 |
|  |  | hsa03050 | Proteasome | 20/2011 | 46/8108 | 4.21E-03 | 2.77E-02 |
|  |  | hsa00520 | Amino sugar and nucleotide sugar metabolism | 20/2011 | 48/8108 | 7.46E-03 | 4.40E-02 |
|  |  | hsa03410 | Base excision repair | 15/2011 | 33/8108 | 7.70E-03 | 4.43E-02 |
|  |  | hsa03030 | DNA replication | 16/2011 | 36/8108 | 7.79E-03 | 4.43E-02 |
|  |  | hsa00531 | Glycosaminoglycan degradation | 10/2011 | 19/8108 | 8.32E-03 | 4.66E-02 |
|  |  | hsa04217 | Necroptosis | 53/2011 | 159/8108 | 9.10E-03 | 4.77E-02 |
|  |  | hsa00900 | Terpenoid backbone biosynthesis | 11/2011 | 22/8108 | 9.29E-03 | 4.79E-02 |
|  | Infectious diseases | hsa05132 | Salmonella infection | 90/2011 | 249/8108 | 3.40E-05 | 1.40E-03 |
|  |  | hsa05161 | Hepatitis B | 62/2011 | 162/8108 | 8.73E-05 | 2.88E-03 |
|  |  | hsa05135 | Yersinia infection | 54/2011 | 137/8108 | 9.94E-05 | 2.98E-03 |
|  |  | hsa05160 | Hepatitis C | 59/2011 | 157/8108 | 2.29E-04 | 4.44E-03 |
|  |  | hsa05166 | Human T-cell leukemia virus 1 infection | 77/2011 | 219/8108 | 3.35E-04 | 5.82E-03 |
|  |  | hsa05170 | Human immunodeficiency virus 1 infection | 74/2011 | 212/8108 | 5.53E-04 | 7.61E-03 |
|  |  | hsa05131 | Shigellosis | 83/2011 | 247/8108 | 1.01E-03 | 1.19E-02 |
|  |  | hsa05164 | Influenza A | 60/2011 | 172/8108 | 1.80E-03 | 1.61E-02 |
|  |  | hsa05130 | Pathogenic Escherichia coli infection | 67/2011 | 197/8108 | 2.11E-03 | 1.78E-02 |
|  |  | hsa05169 | Epstein-Barr virus infection | 68/2011 | 202/8108 | 2.63E-03 | 2.02E-02 |
|  |  | hsa05167 | Kaposi sarcoma-associated herpesvirus infection | 63/2011 | 194/8108 | 9.03E-03 | 4.77E-02 |
|  | Other | hsa04140 | Autophagy - animal | 60/2011 | 141/8108 | 2.45E-06 | 2.02E-04 |
|  |  | hsa04137 | Mitophagy - animal | 30/2011 | 72/8108 | 1.19E-03 | 1.27E-02 |
|  |  | hsa05417 | Lipid and atherosclerosis | 73/2011 | 215/8108 | 1.44E-03 | 1.44E-02 |
|  |  | hsa01524 | Platinum drug resistance | 30/2011 | 73/8108 | 1.54E-03 | 1.46E-02 |
|  |  | hsa04136 | Autophagy - other | 16/2011 | 32/8108 | 1.80E-03 | 1.61E-02 |
| *NEK9* | Cancer-related pathway | hsa04120 | Ubiquitin mediated proteolysis | 71/2262 | 140/8108 | 7.24E-09 | 5.88E-07 |
|  |  | hsa05220 | Chronic myeloid leukemia | 41/2262 | 76/8108 | 1.40E-06 | 5.69E-05 |
|  |  | hsa05212 | Pancreatic cancer | 39/2262 | 76/8108 | 1.24E-05 | 3.04E-04 |
|  |  | hsa05222 | Small cell lung cancer | 43/2262 | 92/8108 | 8.39E-05 | 1.44E-03 |
|  |  | hsa05210 | Colorectal cancer | 38/2262 | 86/8108 | 8.43E-04 | 8.06E-03 |
|  |  | hsa05215 | Prostate cancer | 42/2262 | 97/8108 | 7.68E-04 | 7.80E-03 |
|  |  | hsa04150 | mTOR signaling pathway | 57/2262 | 155/8108 | 9.48E-03 | 4.73E-02 |
|  |  | hsa05211 | Renal cell carcinoma | 33/2262 | 69/8108 | 3.22E-04 | 4.03E-03 |
|  |  | hsa05231 | Choline metabolism in cancer | 40/2262 | 98/8108 | 3.78E-03 | 2.36E-02 |
|  |  | hsa05213 | Endometrial cancer | 26/2262 | 58/8108 | 4.19E-03 | 2.52E-02 |
|  |  | hsa05221 | Acute myeloid leukemia | 29/2262 | 67/8108 | 4.79E-03 | 2.78E-02 |
|  |  | hsa05223 | Non-small cell lung cancer | 34/2262 | 72/8108 | 3.55E-04 | 4.27E-03 |
|  |  | hsa05211 | Renal cell carcinoma | 33/2262 | 69/8108 | 3.22E-04 | 4.03E-03 |
|  |  | hsa01521 | EGFR tyrosine kinase inhibitor resistance | 35/2262 | 79/8108 | 1.26E-03 | 1.07E-02 |
|  |  | hsa04014 | Ras signaling pathway | 85/2262 | 232/8108 | 2.05E-03 | 1.66E-02 |
|  |  | hsa05203 | Viral carcinogenesis | 73/2262 | 204/8108 | 7.84E-03 | 4.14E-02 |
|  |  | hsa03030 | DNA replication | 18/2262 | 36/8108 | 3.97E-03 | 2.44E-02 |
|  | Neural signaling pathway | hsa05017 | Spinocerebellar ataxia | 61/2262 | 143/8108 | 9.42E-05 | 1.47E-03 |
|  |  | hsa05014 | Amyotrophic lateral sclerosis | 126/2262 | 365/8108 | 2.74E-03 | 1.93E-02 |
|  |  | hsa04722 | Neurotrophin signaling pathway | 49/2262 | 119/8108 | 1.15E-03 | 1.01E-02 |
|  | Infectious diseases | hsa05168 | Herpes simplex virus 1 infection | 255/2262 | 498/8108 | 5.95E-30 | 1.93E-27 |
|  |  | hsa05131 | Shigellosis | 98/2262 | 247/8108 | 3.27E-05 | 6.64E-04 |
|  |  | hsa05161 | Hepatitis B | 66/2262 | 162/8108 | 2.54E-04 | 3.44E-03 |
|  |  | hsa05160 | Hepatitis C | 58/2262 | 157/8108 | 8.02E-03 | 4.14E-02 |
|  |  | hsa05163 | Human cytomegalovirus infection | 82/2262 | 225/8108 | 2.88E-03 | 1.95E-02 |
|  |  | hsa05166 | Human T-cell leukemia virus 1 infection | 79/2262 | 219/8108 | 4.62E-03 | 2.73E-02 |
|  |  | hsa05132 | Salmonella infection | 94/2262 | 249/8108 | 3.90E-04 | 4.53E-03 |
|  |  | hsa05170 | Human immunodeficiency virus 1 infection | 81/2262 | 212/8108 | 6.29E-04 | 6.82E-03 |
|  |  | hsa05135 | Yersinia infection | 54/2262 | 137/8108 | 2.17E-03 | 1.72E-02 |
|  |  | hsa05100 | Bacterial invasion of epithelial cells | 32/2262 | 77/8108 | 6.59E-03 | 3.57E-02 |
|  | Metabolism | hsa03040 | Spliceosome | 78/2262 | 147/8108 | 7.85E-11 | 8.50E-09 |
|  |  | hsa04141 | Protein processing in endoplasmic reticulum | 82/2262 | 171/8108 | 1.45E-08 | 9.43E-07 |
|  |  | hsa04114 | Oocyte meiosis | 51/2262 | 129/8108 | 2.64E-03 | 1.90E-02 |
|  |  | hsa04070 | Phosphatidylinositol signaling system | 48/2262 | 97/8108 | 4.78E-06 | 1.41E-04 |
|  |  | hsa00562 | Inositol phosphate metabolism | 38/2262 | 73/8108 | 1.04E-05 | 2.81E-04 |
|  |  | hsa04520 | Adherens junction | 37/2262 | 71/8108 | 1.31E-05 | 3.04E-04 |
|  |  | hsa04144 | Endocytosis | 100/2262 | 252/8108 | 2.70E-05 | 5.86E-04 |
|  |  | hsa04012 | ErbB signaling pathway | 41/2262 | 85/8108 | 4.97E-05 | 8.98E-04 |
|  |  | hsa00310 | Lysine degradation | 32/2262 | 63/8108 | 9.51E-05 | 1.47E-03 |
|  |  | hsa04218 | Cellular senescence | 65/2262 | 156/8108 | 1.31E-04 | 1.93E-03 |
|  |  | hsa03420 | Nucleotide excision repair | 25/2262 | 47/8108 | 2.15E-04 | 3.04E-03 |
|  |  | hsa04530 | Tight junction | 64/2262 | 169/8108 | 2.86E-03 | 1.95E-02 |
|  |  | hsa04210 | Apoptosis | 53/2262 | 136/8108 | 3.16E-03 | 2.05E-02 |
|  |  | hsa04392 | Hippo signaling pathway - multiple species | 15/2262 | 29/8108 | 5.58E-03 | 3.12E-02 |
|  |  | hsa04666 | Fc gamma R-mediated phagocytosis | 40/2262 | 97/8108 | 3.04E-03 | 2.02E-02 |
|  |  | hsa04914 | Progesterone-mediated oocyte maturation | 43/2262 | 100/8108 | 7.95E-04 | 7.83E-03 |
|  |  | hsa04910 | Insulin signaling pathway | 56/2262 | 137/8108 | 6.58E-04 | 6.90E-03 |
|  |  | hsa04330 | Notch signaling pathway | 28/2262 | 59/8108 | 1.04E-03 | 9.41E-03 |
|  |  | hsa04917 | Prolactin signaling pathway | 31/2262 | 70/8108 | 2.35E-03 | 1.82E-02 |
|  |  | hsa04114 | Oocyte meiosis | 51/2262 | 129/8108 | 2.64E-03 | 1.90E-02 |
|  |  | hsa04919 | Thyroid hormone signaling pathway | 46/2262 | 121/8108 | 9.61E-03 | 4.73E-02 |
|  |  | hsa04152 | AMPK signaling pathway | 48/2262 | 120/8108 | 2.63E-03 | 1.90E-02 |
|  | Other | hsa04140 | Autophagy - animal | 65/2262 | 141/8108 | 2.59E-06 | 8.40E-05 |
|  |  | hsa04068 | FoxO signaling pathway | 50/2262 | 131/8108 | 6.55E-03 | 3.57E-02 |
|  |  | hsa03440 | Homologous recombination | 24/2262 | 41/8108 | 3.69E-05 | 7.05E-04 |
|  |  | hsa04931 | Insulin resistance | 42/2262 | 108/8108 | 8.36E-03 | 4.25E-02 |
|  |  | hsa04340 | Hedgehog signaling pathway | 25/2262 | 56/8108 | 5.27E-03 | 3.00E-02 |
|  |  | hsa01522 | Endocrine resistance | 41/2262 | 98/8108 | 1.97E-03 | 1.64E-02 |
|  |  | hsa01524 | Platinum drug resistance | 32/2262 | 73/8108 | 2.46E-03 | 1.86E-02 |
|  |  | hsa03460 | Fanconi anemia pathway | 39/2262 | 54/8108 | 1.50E-11 | 2.44E-09 |
|  |  | hsa03015 | mRNA surveillance pathway | 51/2262 | 98/8108 | 3.36E-07 | 1.82E-05 |
|  |  | hsa03410 | Base excision repair | 17/2262 | 33/8108 | 3.41E-03 | 2.18E-02 |
|  |  | hsa04510 | Focal adhesion | 72/2262 | 201/8108 | 8.01E-03 | 4.14E-02 |
|  |  | hsa03430 | Mismatch repair | 14/2262 | 23/8108 | 9.41E-04 | 8.74E-03 |
|  |  | hsa03013 | RNA transport | 73/2262 | 186/8108 | 4.68E-04 | 5.24E-03 |
|  |  | hsa03018 | RNA degradation | 43/2262 | 79/8108 | 5.61E-07 | 2.60E-05 |
| *USP53* | Cancer-related pathway | hsa04120 | Ubiquitin mediated proteolysis | 89/3311 | 140/8108 | 3.76E-08 | 4.13E-06 |
|  | Infectious diseases | hsa05168 | Herpes simplex virus 1 infection | 324/3311 | 498/8108 | 2.05E-29 | 6.77E-27 |
|  |  | hsa05161 | Hepatitis B | 86/3311 | 162/8108 | 9.80E-04 | 2.69E-02 |
|  | Immune signaling pathway | hsa04622 | RIG-I-like receptor signaling pathway | 42/3311 | 70/8108 | 8.98E-04 | 2.69E-02 |
|  | Neural signaling pathway | hsa05016 | Huntington disease | 156/3311 | 306/8108 | 1.64E-04 | 1.08E-02 |
|  |  | hsa05014 | Amyotrophic lateral sclerosis | 179/3311 | 365/8108 | 7.18E-04 | 2.63E-02 |
|  | Metabolism | hsa04141 | Protein processing in endoplasmic reticulum | 91/3311 | 171/8108 | 6.38E-04 | 2.63E-02 |
|  |  | hsa04144 | Endocytosis | 130/3311 | 252/8108 | 2.98E-04 | 1.64E-02 |
|  |  | hsa04068 | FoxO signaling pathway | 72/3311 | 131/8108 | 6.97E-04 | 2.63E-02 |
|  | Other | hsa04740 | Olfactory transduction | 249/3311 | 443/8108 | 1.45E-11 | 2.39E-09 |
|  |  | hsa04140 | Autophagy - animal | 80/3311 | 141/8108 | 8.82E-05 | 7.28E-03 |
|  |  | hsa04932 | Non-alcoholic fatty liver disease | 83/3311 | 155/8108 | 8.44E-04 | 2.69E-02 |
| *CNOT3* | Cancer-related pathway | hsa04144 | Endocytosis | 95/1804 | 252/8108 | 1.23E-08 | 2.24E-06 |
|  |  | hsa04120 | Ubiquitin mediated proteolysis | 59/1804 | 140/8108 | 8.88E-08 | 7.33E-06 |
|  |  | hsa05220 | Chronic myeloid leukemia | 29/1804 | 76/8108 | 1.19E-03 | 1.51E-02 |
|  |  | hsa05203 | Viral carcinogenesis | 61/1804 | 204/8108 | 6.13E-03 | 4.53E-02 |
|  |  | hsa03030 | DNA replication | 16/1804 | 36/8108 | 2.48E-03 | 2.73E-02 |
|  | Neural signaling pathway | hsa05014 | Amyotrophic lateral sclerosis | 126/1804 | 365/8108 | 2.73E-08 | 3.00E-06 |
|  |  | hsa05022 | Pathways of neurodegeneration - multiple diseases | 149/1804 | 476/8108 | 1.54E-06 | 8.47E-05 |
|  |  | hsa05016 | Huntington disease | 98/1804 | 306/8108 | 3.68E-05 | 1.21E-03 |
|  |  | hsa05012 | Parkinson disease | 82/1804 | 249/8108 | 5.37E-05 | 1.27E-03 |
|  |  | hsa05010 | Alzheimer disease | 112/1804 | 369/8108 | 1.33E-04 | 2.19E-03 |
|  |  | hsa05017 | Spinocerebellar ataxia | 47/1804 | 143/8108 | 2.09E-03 | 2.47E-02 |
|  |  | hsa04722 | Neurotrophin signaling pathway | 39/1804 | 119/8108 | 5.07E-03 | 4.08E-02 |
|  | Metabolism | hsa04330 | Notch signaling pathway | 28/1804 | 59/8108 | 1.58E-05 | 5.81E-04 |
|  |  | hsa04910 | Insulin signaling pathway | 51/1804 | 137/8108 | 4.38E-05 | 1.23E-03 |
|  |  | hsa00190 | Oxidative phosphorylation | 50/1804 | 134/8108 | 4.83E-05 | 1.23E-03 |
|  |  | hsa03022 | Basal transcription factors | 22/1804 | 45/8108 | 7.27E-05 | 1.55E-03 |
|  |  | hsa04150 | mTOR signaling pathway | 55/1804 | 155/8108 | 1.03E-04 | 1.88E-03 |
|  |  | hsa04152 | AMPK signaling pathway | 44/1804 | 120/8108 | 2.13E-04 | 3.20E-03 |
|  |  | hsa04920 | Adipocytokine signaling pathway | 28/1804 | 69/8108 | 4.56E-04 | 6.28E-03 |
|  |  | hsa04070 | Phosphatidylinositol signaling system | 36/1804 | 97/8108 | 5.97E-04 | 7.88E-03 |
|  |  | hsa04142 | Lysosome | 42/1804 | 128/8108 | 3.63E-03 | 3.75E-02 |
|  |  | hsa03040 | Spliceosome | 47/1804 | 147/8108 | 3.86E-03 | 3.77E-02 |
|  |  | hsa04210 | Apoptosis | 44/1804 | 136/8108 | 3.98E-03 | 3.77E-02 |
|  |  | hsa04912 | GnRH signaling pathway | 32/1804 | 93/8108 | 4.70E-03 | 3.97E-02 |
|  |  | hsa04371 | Apelin signaling pathway | 44/1804 | 138/8108 | 5.37E-03 | 4.22E-02 |
|  |  | hsa04530 | Tight junction | 52/1804 | 169/8108 | 5.90E-03 | 4.53E-02 |
|  |  | hsa00562 | Inositol phosphate metabolism | 26/1804 | 73/8108 | 6.18E-03 | 4.53E-02 |
|  |  | hsa04714 | Thermogenesis | 89/1804 | 232/8108 | 1.36E-08 | 2.24E-06 |
|  | Infectious diseases | hsa05132 | Salmonella infection | 87/1804 | 249/8108 | 2.31E-06 | 1.09E-04 |
|  |  | hsa05131 | Shigellosis | 86/1804 | 247/8108 | 3.11E-06 | 1.28E-04 |
|  |  | hsa05130 | Pathogenic Escherichia coli infection | 68/1804 | 197/8108 | 4.52E-05 | 1.23E-03 |
|  |  | hsa05166 | Human T-cell leukemia virus 1 infection | 67/1804 | 219/8108 | 2.29E-03 | 2.60E-02 |
|  |  | hsa05110 | Vibrio cholerae infection | 20/1804 | 50/8108 | 3.50E-03 | 3.73E-02 |
|  |  | hsa05170 | Human immunodeficiency virus 1 infection | 64/1804 | 212/8108 | 4.00E-03 | 3.77E-02 |
|  |  | hsa05167 | Kaposi sarcoma-associated herpesvirus infection | 59/1804 | 194/8108 | 4.67E-03 | 3.97E-02 |
|  |  | hsa05163 | Human cytomegalovirus infection | 67/1804 | 225/8108 | 4.69E-03 | 3.97E-02 |
|  | Other | hsa04140 | Autophagy - animal | 57/1804 | 141/8108 | 7.76E-07 | 5.12E-05 |
|  |  | hsa04136 | Autophagy - other | 17/1804 | 32/8108 | 1.31E-04 | 2.19E-03 |
|  |  | hsa01522 | Endocrine resistance | 38/1804 | 98/8108 | 1.50E-04 | 2.36E-03 |
|  |  | hsa04211 | Longevity regulating pathway | 34/1804 | 89/8108 | 4.55E-04 | 6.28E-03 |
|  |  | hsa05415 | Diabetic cardiomyopathy | 64/1804 | 203/8108 | 1.25E-03 | 1.53E-02 |
|  |  | hsa03018 | RNA degradation | 33/1804 | 79/8108 | 7.49E-05 | 1.55E-03 |
|  |  | hsa03013 | RNA transport | 57/1804 | 186/8108 | 4.49E-03 | 3.97E-02 |
|  |  | hsa04137 | Mitophagy - animal | 26/1804 | 72/8108 | 5.02E-03 | 4.08E-02 |
|  |  | hsa04932 | Non-alcoholic fatty liver disease | 55/1804 | 155/8108 | 1.03E-04 | 1.88E-03 |
| *CDK5RAP2* | Cancer-related pathway | hsa04120 | Ubiquitin mediated proteolysis | 71/2262 | 140/8108 | 7.24E-09 | 5.88E-07 |
|  |  | hsa05220 | Chronic myeloid leukemia | 41/2262 | 76/8108 | 1.40E-06 | 5.69E-05 |
|  |  | hsa05212 | Pancreatic cancer | 39/2262 | 76/8108 | 1.24E-05 | 3.04E-04 |
|  |  | hsa05222 | Small cell lung cancer | 43/2262 | 92/8108 | 8.39E-05 | 1.44E-03 |
|  |  | hsa05211 | Renal cell carcinoma | 33/2262 | 69/8108 | 3.22E-04 | 4.03E-03 |
|  |  | hsa05223 | Non-small cell lung cancer | 34/2262 | 72/8108 | 3.55E-04 | 4.27E-03 |
|  |  | hsa05215 | Prostate cancer | 42/2262 | 97/8108 | 7.68E-04 | 7.80E-03 |
|  |  | hsa05210 | Colorectal cancer | 38/2262 | 86/8108 | 8.43E-04 | 8.06E-03 |
|  |  | hsa01521 | EGFR tyrosine kinase inhibitor resistance | 35/2262 | 79/8108 | 1.26E-03 | 1.07E-02 |
|  |  | hsa04014 | Ras signaling pathway | 85/2262 | 232/8108 | 2.05E-03 | 1.66E-02 |
|  |  | hsa05231 | Choline metabolism in cancer | 40/2262 | 98/8108 | 3.78E-03 | 2.36E-02 |
|  |  | hsa05213 | Endometrial cancer | 26/2262 | 58/8108 | 4.19E-03 | 2.52E-02 |
|  |  | hsa05221 | Acute myeloid leukemia | 29/2262 | 67/8108 | 4.79E-03 | 2.78E-02 |
|  |  | hsa05203 | Viral carcinogenesis | 73/2262 | 204/8108 | 7.84E-03 | 4.14E-02 |
|  |  | hsa03030 | DNA replication | 18/2262 | 36/8108 | 3.97E-03 | 2.44E-02 |
|  | Neural signaling pathway | hsa05017 | Spinocerebellar ataxia | 61/2262 | 143/8108 | 9.42E-05 | 1.47E-03 |
|  |  | hsa04722 | Neurotrophin signaling pathway | 49/2262 | 119/8108 | 1.15E-03 | 1.01E-02 |
|  |  | hsa05014 | Amyotrophic lateral sclerosis | 126/2262 | 365/8108 | 2.74E-03 | 1.93E-02 |
|  | Metabolism | hsa03040 | Spliceosome | 78/2262 | 147/8108 | 7.85E-11 | 8.50E-09 |
|  |  | hsa04141 | Protein processing in endoplasmic reticulum | 82/2262 | 171/8108 | 1.45E-08 | 9.43E-07 |
|  |  | hsa04110 | Cell cycle | 59/2262 | 124/8108 | 2.11E-06 | 7.62E-05 |
|  |  | hsa04070 | Phosphatidylinositol signaling system | 48/2262 | 97/8108 | 4.78E-06 | 1.41E-04 |
|  |  | hsa00562 | Inositol phosphate metabolism | 38/2262 | 73/8108 | 1.04E-05 | 2.81E-04 |
|  |  | hsa04520 | Adherens junction | 37/2262 | 71/8108 | 1.31E-05 | 3.04E-04 |
|  |  | hsa04144 | Endocytosis | 100/2262 | 252/8108 | 2.70E-05 | 5.86E-04 |
|  |  | hsa03440 | Homologous recombination | 24/2262 | 41/8108 | 3.69E-05 | 7.05E-04 |
|  |  | hsa04012 | ErbB signaling pathway | 41/2262 | 85/8108 | 4.97E-05 | 8.98E-04 |
|  |  | hsa00310 | Lysine degradation | 32/2262 | 63/8108 | 9.51E-05 | 1.47E-03 |
|  |  | hsa04218 | Cellular senescence | 65/2262 | 156/8108 | 1.31E-04 | 1.93E-03 |
|  |  | hsa03420 | Nucleotide excision repair | 25/2262 | 47/8108 | 2.15E-04 | 3.04E-03 |
|  |  | hsa04935 | Growth hormone synthesis, secretion and action | 51/2262 | 119/8108 | 2.99E-04 | 3.89E-03 |
|  |  | hsa04910 | Insulin signaling pathway | 56/2262 | 137/8108 | 6.58E-04 | 6.90E-03 |
|  |  | hsa04914 | Progesterone-mediated oocyte maturation | 43/2262 | 100/8108 | 7.95E-04 | 7.83E-03 |
|  |  | hsa04330 | Notch signaling pathway | 28/2262 | 59/8108 | 1.04E-03 | 9.41E-03 |
|  |  | hsa01522 | Endocrine resistance | 41/2262 | 98/8108 | 1.97E-03 | 1.64E-02 |
|  |  | hsa04917 | Prolactin signaling pathway | 31/2262 | 70/8108 | 2.35E-03 | 1.82E-02 |
|  |  | hsa04152 | AMPK signaling pathway | 48/2262 | 120/8108 | 2.63E-03 | 1.90E-02 |
|  |  | hsa04114 | Oocyte meiosis | 51/2262 | 129/8108 | 2.64E-03 | 1.90E-02 |
|  |  | hsa04530 | Tight junction | 64/2262 | 169/8108 | 2.86E-03 | 1.95E-02 |
|  |  | hsa04666 | Fc gamma R-mediated phagocytosis | 40/2262 | 97/8108 | 3.04E-03 | 2.02E-02 |
|  |  | hsa04210 | Apoptosis | 53/2262 | 136/8108 | 3.16E-03 | 2.05E-02 |
|  |  | hsa04340 | Hedgehog signaling pathway | 25/2262 | 56/8108 | 5.27E-03 | 3.00E-02 |
|  |  | hsa04392 | Hippo signaling pathway - multiple species | 15/2262 | 29/8108 | 5.58E-03 | 3.12E-02 |
|  |  | hsa04068 | FoxO signaling pathway | 50/2262 | 131/8108 | 6.55E-03 | 3.57E-02 |
|  |  | hsa04150 | mTOR signaling pathway | 57/2262 | 155/8108 | 9.48E-03 | 4.73E-02 |
|  |  | hsa04919 | Thyroid hormone signaling pathway | 46/2262 | 121/8108 | 9.61E-03 | 4.73E-02 |
|  | Infectious diseases | hsa05168 | Herpes simplex virus 1 infection | 255/2262 | 498/8108 | 5.95E-30 | 1.93E-27 |
|  |  | hsa05131 | Shigellosis | 98/2262 | 247/8108 | 3.27E-05 | 6.64E-04 |
|  |  | hsa05161 | Hepatitis B | 66/2262 | 162/8108 | 2.54E-04 | 3.44E-03 |
|  |  | hsa05132 | Salmonella infection | 94/2262 | 249/8108 | 3.90E-04 | 4.53E-03 |
|  |  | hsa05170 | Human immunodeficiency virus 1 infection | 81/2262 | 212/8108 | 6.29E-04 | 6.82E-03 |
|  |  | hsa05135 | Yersinia infection | 54/2262 | 137/8108 | 2.17E-03 | 1.72E-02 |
|  |  | hsa05163 | Human cytomegalovirus infection | 82/2262 | 225/8108 | 2.88E-03 | 1.95E-02 |
|  |  | hsa05166 | Human T-cell leukemia virus 1 infection | 79/2262 | 219/8108 | 4.62E-03 | 2.73E-02 |
|  |  | hsa05100 | Bacterial invasion of epithelial cells | 32/2262 | 77/8108 | 6.59E-03 | 3.57E-02 |
|  |  | hsa05160 | Hepatitis C | 58/2262 | 157/8108 | 8.02E-03 | 4.14E-02 |
|  | Other | hsa04140 | Autophagy - animal | 65/2262 | 141/8108 | 2.59E-06 | 8.40E-05 |
|  |  | hsa01524 | Platinum drug resistance | 32/2262 | 73/8108 | 2.46E-03 | 1.86E-02 |
|  |  | hsa04510 | Focal adhesion | 72/2262 | 201/8108 | 8.01E-03 | 4.14E-02 |
|  |  | hsa04931 | Insulin resistance | 42/2262 | 108/8108 | 8.36E-03 | 4.25E-02 |
|  |  | hsa03015 | mRNA surveillance pathway | 51/2262 | 98/8108 | 3.36E-07 | 1.82E-05 |
|  |  | hsa03018 | RNA degradation | 43/2262 | 79/8108 | 5.61E-07 | 2.60E-05 |
|  |  | hsa03013 | RNA transport | 73/2262 | 186/8108 | 4.68E-04 | 5.24E-03 |
|  |  | hsa03430 | Mismatch repair | 14/2262 | 23/8108 | 9.41E-04 | 8.74E-03 |
|  |  | hsa03410 | Base excision repair | 17/2262 | 33/8108 | 3.41E-03 | 2.18E-02 |
|  |  | hsa03460 | Fanconi anemia pathway | 39/2262 | 54/8108 | 1.50E-11 | 2.44E-09 |
| *ING5* | Metabolism | hsa03040 | Spliceosome | 63/1536 | 147/8108 | 1.50E-11 | 2.42E-09 |
|  |  | hsa04110 | Cell cycle | 44/1536 | 124/8108 | 9.18E-06 | 4.92E-04 |
|  |  | hsa00310 | Lysine degradation | 25/1536 | 63/8108 | 1.01E-04 | 4.65E-03 |
|  |  | hsa03008 | Ribosome biogenesis in eukaryotes | 36/1536 | 110/8108 | 3.73E-04 | 1.38E-02 |
|  | Infectious diseases | hsa05168 | Herpes simplex virus 1 infection | 159/1536 | 498/8108 | 5.58E-13 | 1.80E-10 |
|  | Other | hsa03013 | RNA transport | 70/1536 | 186/8108 | 1.28E-09 | 1.38E-07 |
|  |  | hsa03015 | mRNA surveillance pathway | 42/1536 | 98/8108 | 3.80E-08 | 3.06E-06 |
|  |  | hsa03018 | RNA degradation | 28/1536 | 79/8108 | 3.85E-04 | 1.38E-02 |
|  |  | hsa03022 | Basal transcription factors | 18/1536 | 45/8108 | 8.29E-04 | 2.67E-02 |
|  |  | hsa03460 | Fanconi anemia pathway | 25/1536 | 54/8108 | 4.06E-06 | 2.61E-04 |
| *HMGXB4* | Cancer-related pathway | hsa04120 | Ubiquitin mediated proteolysis | 100/2780 | 140/8108 | 1.74E-19 | 5.74E-17 |
|  |  | hsa04150 | mTOR signaling pathway | 82/2780 | 155/8108 | 1.20E-06 | 2.34E-05 |
|  |  | hsa05210 | Colorectal cancer | 50/2780 | 86/8108 | 4.63E-06 | 8.49E-05 |
|  |  | hsa05211 | Renal cell carcinoma | 40/2780 | 69/8108 | 4.50E-05 | 6.45E-04 |
|  |  | hsa05220 | Chronic myeloid leukemia | 43/2780 | 76/8108 | 5.27E-05 | 7.25E-04 |
|  |  | hsa05212 | Pancreatic cancer | 42/2780 | 76/8108 | 1.33E-04 | 1.51E-03 |
|  |  | hsa05215 | Prostate cancer | 48/2780 | 97/8108 | 1.36E-03 | 9.92E-03 |
|  |  | hsa04115 | p53 signaling pathway | 37/2780 | 73/8108 | 2.74E-03 | 1.70E-02 |
|  |  | hsa05213 | Endometrial cancer | 29/2780 | 58/8108 | 9.57E-03 | 4.86E-02 |
|  |  | hsa03030 | DNA replication | 24/2780 | 36/8108 | 7.19E-05 | 9.13E-04 |
|  | Neural signaling pathway | hsa04071 | Sphingolipid signaling pathway | 61/2780 | 119/8108 | 9.25E-05 | 1.13E-03 |
|  |  | hsa05014 | Amyotrophic lateral sclerosis | 158/2780 | 365/8108 | 1.62E-04 | 1.69E-03 |
|  |  | hsa05017 | Spinocerebellar ataxia | 67/2780 | 143/8108 | 1.16E-03 | 8.70E-03 |
|  |  | hsa04722 | Neurotrophin signaling pathway | 57/2780 | 119/8108 | 1.38E-03 | 9.92E-03 |
|  |  | hsa04720 | Long-term potentiation | 33/2780 | 67/8108 | 7.90E-03 | 4.21E-02 |
|  | Metabolism | hsa04110 | Cell cycle | 82/2780 | 124/8108 | 3.31E-13 | 3.64E-11 |
|  |  | hsa04141 | Protein processing in endoplasmic reticulum | 98/2780 | 171/8108 | 4.16E-10 | 1.78E-08 |
|  |  | hsa03040 | Spliceosome | 87/2780 | 147/8108 | 4.31E-10 | 1.78E-08 |
|  |  | hsa04144 | Endocytosis | 130/2780 | 252/8108 | 7.49E-09 | 2.74E-07 |
|  |  | hsa04218 | Cellular senescence | 86/2780 | 156/8108 | 5.89E-08 | 1.87E-06 |
|  |  | hsa03440 | Homologous recombination | 31/2780 | 41/8108 | 7.20E-08 | 1.98E-06 |
|  |  | hsa03015 | mRNA surveillance pathway | 57/2780 | 98/8108 | 9.93E-07 | 2.05E-05 |
|  |  | hsa00562 | Inositol phosphate metabolism | 43/2780 | 73/8108 | 1.35E-05 | 2.35E-04 |
|  |  | hsa04012 | ErbB signaling pathway | 48/2780 | 85/8108 | 2.11E-05 | 3.48E-04 |
|  |  | hsa04114 | Oocyte meiosis | 67/2780 | 129/8108 | 2.47E-05 | 3.88E-04 |
|  |  | hsa03420 | Nucleotide excision repair | 30/2780 | 47/8108 | 3.24E-05 | 4.86E-04 |
|  |  | hsa04070 | Phosphatidylinositol signaling system | 52/2780 | 97/8108 | 6.63E-05 | 8.76E-04 |
|  |  | hsa04210 | Apoptosis | 68/2780 | 136/8108 | 1.02E-04 | 1.20E-03 |
|  |  | hsa04130 | SNARE interactions in vesicular transport | 22/2780 | 33/8108 | 1.44E-04 | 1.59E-03 |
|  |  | hsa04068 | FoxO signaling pathway | 64/2780 | 131/8108 | 3.71E-04 | 3.71E-03 |
|  |  | hsa00310 | Lysine degradation | 35/2780 | 63/8108 | 4.14E-04 | 4.02E-03 |
|  |  | hsa04520 | Adherens junction | 38/2780 | 71/8108 | 6.43E-04 | 5.44E-03 |
|  |  | hsa04910 | Insulin signaling pathway | 65/2780 | 137/8108 | 9.09E-04 | 7.37E-03 |
|  |  | hsa04152 | AMPK signaling pathway | 58/2780 | 120/8108 | 9.57E-04 | 7.52E-03 |
|  |  | hsa03020 | RNA polymerase | 19/2780 | 31/8108 | 1.88E-03 | 1.29E-02 |
|  |  | hsa03450 | Non-homologous end-joining | 10/2780 | 13/8108 | 2.09E-03 | 1.37E-02 |
|  |  | hsa03060 | Protein export | 15/2780 | 23/8108 | 2.38E-03 | 1.51E-02 |
|  |  | hsa03008 | Ribosome biogenesis in eukaryotes | 52/2780 | 110/8108 | 3.12E-03 | 1.87E-02 |
|  |  | hsa00563 | Glycosylphosphatidylinositol (GPI)-anchor biosynthesis | 16/2780 | 26/8108 | 4.04E-03 | 2.38E-02 |
|  |  | hsa04146 | Peroxisome | 40/2780 | 82/8108 | 4.55E-03 | 2.63E-02 |
|  |  | hsa00900 | Terpenoid backbone biosynthesis | 14/2780 | 22/8108 | 4.63E-03 | 2.63E-02 |
|  |  | hsa04914 | Progesterone-mediated oocyte maturation | 47/2780 | 100/8108 | 5.51E-03 | 3.04E-02 |
|  |  | hsa04810 | Regulation of actin cytoskeleton | 92/2780 | 218/8108 | 8.33E-03 | 4.37E-02 |
|  |  | hsa04215 | Apoptosis - multiple species | 18/2780 | 32/8108 | 8.75E-03 | 4.51E-02 |
|  |  | hsa04714 | Thermogenesis | 100/2780 | 232/8108 | 2.88E-03 | 1.76E-02 |
|  | Infectious diseases | hsa05168 | Herpes simplex virus 1 infection | 247/2780 | 498/8108 | 2.79E-13 | 3.64E-11 |
|  |  | hsa05131 | Shigellosis | 125/2780 | 247/8108 | 6.23E-08 | 1.87E-06 |
|  |  | hsa05132 | Salmonella infection | 122/2780 | 249/8108 | 8.45E-07 | 1.86E-05 |
|  |  | hsa05161 | Hepatitis B | 78/2780 | 162/8108 | 1.63E-04 | 1.69E-03 |
|  |  | hsa05100 | Bacterial invasion of epithelial cells | 41/2780 | 77/8108 | 4.60E-04 | 4.33E-03 |
|  |  | hsa05135 | Yersinia infection | 66/2780 | 137/8108 | 4.96E-04 | 4.55E-03 |
|  |  | hsa05160 | Hepatitis C | 74/2780 | 157/8108 | 5.31E-04 | 4.73E-03 |
|  |  | hsa05130 | Pathogenic Escherichia coli infection | 88/2780 | 197/8108 | 1.43E-03 | 1.00E-02 |
|  |  | hsa05170 | Human immunodeficiency virus 1 infection | 93/2780 | 212/8108 | 2.11E-03 | 1.37E-02 |
|  |  | hsa05110 | Vibrio cholerae infection | 26/2780 | 50/8108 | 7.31E-03 | 3.95E-02 |
|  | Other | hsa04140 | Autophagy - animal | 87/2780 | 141/8108 | 1.93E-11 | 1.59E-09 |
|  |  | hsa04137 | Mitophagy - animal | 38/2780 | 72/8108 | 9.16E-04 | 7.37E-03 |
|  |  | hsa04136 | Autophagy - other | 20/2780 | 32/8108 | 1.01E-03 | 7.75E-03 |
|  |  | hsa01521 | EGFR tyrosine kinase inhibitor resistance | 40/2780 | 79/8108 | 1.93E-03 | 1.30E-02 |
|  |  | hsa01524 | Platinum drug resistance | 36/2780 | 73/8108 | 5.53E-03 | 3.04E-02 |
|  |  | hsa03460 | Fanconi anemia pathway | 42/2780 | 54/8108 | 6.68E-11 | 4.41E-09 |
|  |  | hsa03013 | RNA transport | 105/2780 | 186/8108 | 3.28E-10 | 1.78E-08 |
|  |  | hsa03018 | RNA degradation | 50/2780 | 79/8108 | 1.19E-07 | 3.03E-06 |
|  |  | hsa03430 | Mismatch repair | 16/2780 | 23/8108 | 5.85E-04 | 5.08E-03 |
|  |  | hsa03022 | Basal transcription factors | 32/2780 | 45/8108 | 4.81E-07 | 1.13E-05 |
| *SH3D19* | Cancer-related pathway | hsa04120 | Ubiquitin mediated proteolysis | 96/2767 | 140/8108 | 5.53E-17 | 9.13E-15 |
|  |  | hsa05210 | Colorectal cancer | 51/2767 | 86/8108 | 1.40E-06 | 5.14E-05 |
|  |  | hsa05220 | Chronic myeloid leukemia | 43/2767 | 76/8108 | 4.64E-05 | 1.02E-03 |
|  |  | hsa05212 | Pancreatic cancer | 41/2767 | 76/8108 | 2.86E-04 | 4.49E-03 |
|  |  | hsa04150 | mTOR signaling pathway | 73/2767 | 155/8108 | 5.09E-04 | 6.82E-03 |
|  |  | hsa05211 | Renal cell carcinoma | 37/2767 | 69/8108 | 6.49E-04 | 8.24E-03 |
|  |  | hsa05231 | Choline metabolism in cancer | 49/2767 | 98/8108 | 8.08E-04 | 9.11E-03 |
|  |  | hsa05223 | Non-small cell lung cancer | 36/2767 | 72/8108 | 3.81E-03 | 2.73E-02 |
|  |  | hsa05213 | Endometrial cancer | 30/2767 | 58/8108 | 4.21E-03 | 2.73E-02 |
|  |  | hsa04932 | Non-alcoholic fatty liver disease | 69/2767 | 155/8108 | 4.32E-03 | 2.73E-02 |
|  |  | hsa04350 | TGF-beta signaling pathway | 44/2767 | 94/8108 | 7.07E-03 | 4.24E-02 |
|  | Neural signaling pathway | hsa05014 | Amyotrophic lateral sclerosis | 161/2767 | 365/8108 | 3.28E-05 | 7.73E-04 |
|  |  | hsa04071 | Sphingolipid signaling pathway | 61/2767 | 119/8108 | 7.94E-05 | 1.64E-03 |
|  |  | hsa05016 | Huntington disease | 131/2767 | 306/8108 | 7.93E-04 | 9.11E-03 |
|  |  | hsa05017 | Spinocerebellar ataxia | 67/2767 | 143/8108 | 1.01E-03 | 1.01E-02 |
|  |  | hsa04722 | Neurotrophin signaling pathway | 57/2767 | 119/8108 | 1.22E-03 | 1.12E-02 |
|  |  | hsa05010 | Alzheimer disease | 150/2767 | 369/8108 | 4.39E-03 | 2.73E-02 |
|  | Immune signaling pathway | hsa05235 | PD-L1 expression and PD-1 checkpoint pathway in cancer | 44/2767 | 89/8108 | 1.95E-03 | 1.69E-02 |
|  | Metabolism | hsa04068 | FoxO signaling pathway | 76/2767 | 131/8108 | 1.48E-08 | 1.22E-06 |
|  |  | hsa03018 | RNA degradation | 50/2767 | 79/8108 | 1.01E-07 | 6.44E-06 |
|  |  | hsa04520 | Adherens junction | 46/2767 | 71/8108 | 1.17E-07 | 6.44E-06 |
|  |  | hsa04144 | Endocytosis | 124/2767 | 252/8108 | 3.85E-07 | 1.82E-05 |
|  |  | hsa04110 | Cell cycle | 69/2767 | 124/8108 | 6.14E-07 | 2.53E-05 |
|  |  | hsa04218 | Cellular senescence | 80/2767 | 156/8108 | 6.39E-06 | 2.11E-04 |
|  |  | hsa03015 | mRNA surveillance pathway | 54/2767 | 98/8108 | 1.48E-05 | 3.95E-04 |
|  |  | hsa03440 | Homologous recombination | 26/2767 | 41/8108 | 1.17E-04 | 2.27E-03 |
|  |  | hsa04152 | AMPK signaling pathway | 60/2767 | 120/8108 | 2.21E-04 | 4.06E-03 |
|  |  | hsa03040 | Spliceosome | 71/2767 | 147/8108 | 2.40E-04 | 4.16E-03 |
|  |  | hsa04012 | ErbB signaling pathway | 45/2767 | 85/8108 | 2.60E-04 | 4.30E-03 |
|  |  | hsa03013 | RNA transport | 86/2767 | 186/8108 | 3.63E-04 | 5.45E-03 |
|  |  | hsa04130 | SNARE interactions in vesicular transport | 21/2767 | 33/8108 | 4.96E-04 | 6.82E-03 |
|  |  | hsa00562 | Inositol phosphate metabolism | 39/2767 | 73/8108 | 5.17E-04 | 6.82E-03 |
|  |  | hsa03022 | Basal transcription factors | 26/2767 | 45/8108 | 9.47E-04 | 9.76E-03 |
|  |  | hsa04141 | Protein processing in endoplasmic reticulum | 78/2767 | 171/8108 | 1.09E-03 | 1.06E-02 |
|  |  | hsa04070 | Phosphatidylinositol signaling system | 48/2767 | 97/8108 | 1.21E-03 | 1.12E-02 |
|  |  | hsa04114 | Oocyte meiosis | 61/2767 | 129/8108 | 1.25E-03 | 1.12E-02 |
|  |  | hsa04917 | Prolactin signaling pathway | 36/2767 | 70/8108 | 2.05E-03 | 1.74E-02 |
|  |  | hsa04919 | Thyroid hormone signaling pathway | 56/2767 | 121/8108 | 3.52E-03 | 2.73E-02 |
|  |  | hsa03050 | Proteasome | 25/2767 | 46/8108 | 3.73E-03 | 2.73E-02 |
|  |  | hsa04211 | Longevity regulating pathway | 43/2767 | 89/8108 | 3.78E-03 | 2.73E-02 |
|  |  | hsa00563 | Glycosylphosphatidylinositol (GPI)-anchor biosynthesis | 16/2767 | 26/8108 | 3.83E-03 | 2.73E-02 |
|  |  | hsa04146 | Peroxisome | 40/2767 | 82/8108 | 4.14E-03 | 2.73E-02 |
|  |  | hsa04910 | Insulin signaling pathway | 62/2767 | 137/8108 | 4.21E-03 | 2.73E-02 |
|  |  | hsa04931 | Insulin resistance | 50/2767 | 108/8108 | 5.59E-03 | 3.42E-02 |
|  | Infectious diseases | hsa05168 | Herpes simplex virus 1 infection | 306/2767 | 498/8108 | 7.64E-38 | 2.52E-35 |
|  |  | hsa05132 | Salmonella infection | 118/2767 | 249/8108 | 7.74E-06 | 2.32E-04 |
|  |  | hsa05131 | Shigellosis | 116/2767 | 247/8108 | 1.55E-05 | 3.95E-04 |
|  |  | hsa05135 | Yersinia infection | 65/2767 | 137/8108 | 7.90E-04 | 9.11E-03 |
|  |  | hsa05161 | Hepatitis B | 72/2767 | 162/8108 | 3.78E-03 | 2.73E-02 |
|  |  | hsa05163 | Human cytomegalovirus infection | 96/2767 | 225/8108 | 4.24E-03 | 2.73E-02 |
|  |  | hsa05120 | Epithelial cell signaling in Helicobacter pylori infection | 35/2767 | 70/8108 | 4.29E-03 | 2.73E-02 |
|  |  | hsa05100 | Bacterial invasion of epithelial cells | 37/2767 | 77/8108 | 7.73E-03 | 4.56E-02 |
|  | Other | hsa04140 | Autophagy - animal | 83/2767 | 141/8108 | 1.21E-09 | 1.34E-07 |
|  |  | hsa04137 | Mitophagy - animal | 38/2767 | 72/8108 | 8.28E-04 | 9.11E-03 |
|  |  | hsa04136 | Autophagy - other | 20/2767 | 32/8108 | 9.44E-04 | 9.76E-03 |
|  |  | hsa04714 | Thermogenesis | 100/2767 | 232/8108 | 2.44E-03 | 2.02E-02 |
| *UBE2H* | Cancer-related pathway | hsa04120 | Ubiquitin mediated proteolysis | 85/2670 | 140/8108 | 1.01E-11 | 1.66E-09 |
|  |  | hsa01521 | EGFR tyrosine kinase inhibitor resistance | 50/2670 | 79/8108 | 2.76E-08 | 1.00E-06 |
|  |  | hsa04151 | PI3K-Akt signaling pathway | 165/2670 | 354/8108 | 3.19E-08 | 1.04E-06 |
|  |  | hsa05215 | Prostate cancer | 58/2670 | 97/8108 | 4.38E-08 | 1.31E-06 |
|  |  | hsa05212 | Pancreatic cancer | 48/2670 | 76/8108 | 5.76E-08 | 1.46E-06 |
|  |  | hsa05205 | Proteoglycans in cancer | 104/2670 | 205/8108 | 7.26E-08 | 1.70E-06 |
|  |  | hsa05220 | Chronic myeloid leukemia | 47/2670 | 76/8108 | 2.01E-07 | 4.39E-06 |
|  |  | hsa05211 | Renal cell carcinoma | 42/2670 | 69/8108 | 1.64E-06 | 2.34E-05 |
|  |  | hsa04010 | MAPK signaling pathway | 135/2670 | 294/8108 | 1.63E-06 | 2.34E-05 |
|  |  | hsa04340 | Hedgehog signaling pathway | 36/2670 | 56/8108 | 1.42E-06 | 2.22E-05 |
|  |  | hsa05222 | Small cell lung cancer | 52/2670 | 92/8108 | 2.46E-06 | 2.98E-05 |
|  |  | hsa04014 | Ras signaling pathway | 109/2670 | 232/8108 | 4.54E-06 | 5.13E-05 |
|  |  | hsa05210 | Colorectal cancer | 48/2670 | 86/8108 | 9.52E-06 | 1.01E-04 |
|  |  | hsa05231 | Choline metabolism in cancer | 53/2670 | 98/8108 | 1.15E-05 | 1.18E-04 |
|  |  | hsa05223 | Non-small cell lung cancer | 40/2670 | 72/8108 | 5.99E-05 | 5.46E-04 |
|  |  | hsa05221 | Acute myeloid leukemia | 37/2670 | 67/8108 | 1.33E-04 | 1.14E-03 |
|  |  | hsa05213 | Endometrial cancer | 33/2670 | 58/8108 | 1.41E-04 | 1.15E-03 |
|  |  | hsa05218 | Melanoma | 39/2670 | 72/8108 | 1.54E-04 | 1.23E-03 |
|  |  | hsa04150 | mTOR signaling pathway | 72/2670 | 155/8108 | 2.82E-04 | 1.99E-03 |
|  |  | hsa05214 | Glioma | 40/2670 | 75/8108 | 1.99E-04 | 1.48E-03 |
|  |  | hsa05225 | Hepatocellular carcinoma | 75/2670 | 168/8108 | 9.15E-04 | 5.18E-03 |
|  |  | hsa04932 | Non-alcoholic fatty liver disease | 67/2670 | 155/8108 | 4.39E-03 | 2.08E-02 |
|  |  | hsa04350 | TGF-beta signaling pathway | 42/2670 | 94/8108 | 1.11E-02 | 4.46E-02 |
|  |  | hsa05224 | Breast cancer | 62/2670 | 147/8108 | 1.12E-02 | 4.46E-02 |
|  | Neural signaling pathway | hsa05010 | Alzheimer disease | 154/2670 | 369/8108 | 1.80E-04 | 1.38E-03 |
|  |  | hsa04071 | Sphingolipid signaling pathway | 58/2670 | 119/8108 | 2.29E-04 | 1.67E-03 |
|  |  | hsa04722 | Neurotrophin signaling pathway | 57/2670 | 119/8108 | 4.54E-04 | 2.92E-03 |
|  |  | hsa05014 | Amyotrophic lateral sclerosis | 150/2670 | 365/8108 | 5.02E-04 | 3.17E-03 |
|  |  | hsa05022 | Pathways of neurodegeneration - multiple diseases | 188/2670 | 476/8108 | 1.13E-03 | 6.18E-03 |
|  |  | hsa05016 | Huntington disease | 125/2670 | 306/8108 | 1.86E-03 | 9.40E-03 |
|  |  | hsa05017 | Spinocerebellar ataxia | 62/2670 | 143/8108 | 5.52E-03 | 2.51E-02 |
|  | Metabolism | hsa04144 | Endocytosis | 131/2670 | 252/8108 | 1.80E-10 | 1.47E-08 |
|  |  | hsa04141 | Protein processing in endoplasmic reticulum | 96/2670 | 171/8108 | 2.46E-10 | 1.61E-08 |
|  |  | hsa04810 | Regulation of actin cytoskeleton | 114/2670 | 218/8108 | 1.78E-09 | 8.32E-08 |
|  |  | hsa04520 | Adherens junction | 48/2670 | 71/8108 | 2.07E-09 | 8.50E-08 |
|  |  | hsa04360 | Axon guidance | 92/2670 | 182/8108 | 5.07E-07 | 9.23E-06 |
|  |  | hsa03015 | mRNA surveillance pathway | 55/2670 | 98/8108 | 1.72E-06 | 2.35E-05 |
|  |  | hsa04530 | Tight junction | 85/2670 | 169/8108 | 1.79E-06 | 2.35E-05 |
|  |  | hsa04012 | ErbB signaling pathway | 49/2670 | 85/8108 | 2.20E-06 | 2.78E-05 |
|  |  | hsa04218 | Cellular senescence | 79/2670 | 156/8108 | 2.93E-06 | 3.44E-05 |
|  |  | hsa04068 | FoxO signaling pathway | 68/2670 | 131/8108 | 4.77E-06 | 5.21E-05 |
|  |  | hsa04710 | Circadian rhythm | 22/2670 | 31/8108 | 1.60E-05 | 1.59E-04 |
|  |  | hsa04512 | ECM-receptor interaction | 48/2670 | 88/8108 | 2.18E-05 | 2.11E-04 |
|  |  | hsa03040 | Spliceosome | 72/2670 | 147/8108 | 3.44E-05 | 3.23E-04 |
|  |  | hsa04910 | Insulin signaling pathway | 66/2670 | 137/8108 | 1.35E-04 | 1.14E-03 |
|  |  | hsa04072 | Phospholipase D signaling pathway | 70/2670 | 148/8108 | 1.75E-04 | 1.36E-03 |
|  |  | hsa04130 | SNARE interactions in vesicular transport | 21/2670 | 33/8108 | 2.85E-04 | 1.99E-03 |
|  |  | hsa04392 | Hippo signaling pathway - multiple species | 19/2670 | 29/8108 | 3.22E-04 | 2.20E-03 |
|  |  | hsa04931 | Insulin resistance | 53/2670 | 108/8108 | 3.38E-04 | 2.23E-03 |
|  |  | hsa04371 | Apelin signaling pathway | 65/2670 | 138/8108 | 3.39E-04 | 2.23E-03 |
|  |  | hsa04919 | Thyroid hormone signaling pathway | 57/2670 | 121/8108 | 7.64E-04 | 4.56E-03 |
|  |  | hsa04110 | Cell cycle | 58/2670 | 124/8108 | 8.59E-04 | 5.03E-03 |
|  |  | hsa04152 | AMPK signaling pathway | 56/2670 | 120/8108 | 1.12E-03 | 6.18E-03 |
|  |  | hsa04390 | Hippo signaling pathway | 70/2670 | 157/8108 | 1.39E-03 | 7.48E-03 |
|  |  | hsa04015 | Rap1 signaling pathway | 90/2670 | 210/8108 | 1.47E-03 | 7.79E-03 |
|  |  | hsa03018 | RNA degradation | 39/2670 | 79/8108 | 1.71E-03 | 8.78E-03 |
|  |  | hsa04070 | Phosphatidylinositol signaling system | 46/2670 | 97/8108 | 2.00E-03 | 9.95E-03 |
|  |  | hsa04714 | Thermogenesis | 97/2670 | 232/8108 | 2.52E-03 | 1.23E-02 |
|  |  | hsa04211 | Longevity regulating pathway | 42/2670 | 89/8108 | 3.44E-03 | 1.66E-02 |
|  |  | hsa00562 | Inositol phosphate metabolism | 35/2670 | 73/8108 | 5.28E-03 | 2.48E-02 |
|  |  | hsa05167 | Kaposi sarcoma-associated herpesvirus infection | 81/2670 | 194/8108 | 5.70E-03 | 2.56E-02 |
|  |  | hsa05161 | Hepatitis B | 69/2670 | 162/8108 | 5.92E-03 | 2.62E-02 |
|  |  | hsa05163 | Human cytomegalovirus infection | 92/2670 | 225/8108 | 6.76E-03 | 2.96E-02 |
|  |  | hsa04666 | Fc gamma R-mediated phagocytosis | 44/2670 | 97/8108 | 6.90E-03 | 2.98E-02 |
|  |  | hsa04926 | Relaxin signaling pathway | 56/2670 | 129/8108 | 7.83E-03 | 3.34E-02 |
|  |  | hsa04935 | Growth hormone synthesis, secretion and action | 52/2670 | 119/8108 | 8.72E-03 | 3.67E-02 |
|  |  | hsa04917 | Prolactin signaling pathway | 33/2670 | 70/8108 | 9.09E-03 | 3.73E-02 |
|  |  | hsa00532 | Glycosaminoglycan biosynthesis - chondroitin sulfate / dermatan sulfate | 12/2670 | 20/8108 | 1.16E-02 | 4.53E-02 |
|  |  | hsa04114 | Oocyte meiosis | 55/2670 | 129/8108 | 1.27E-02 | 4.91E-02 |
|  |  | hsa04914 | Progesterone-mediated oocyte maturation | 44/2670 | 100/8108 | 1.31E-02 | 4.98E-02 |
|  |  | hsa03013 | RNA transport | 76/2670 | 186/8108 | 1.32E-02 | 4.98E-02 |
|  | Infectious diseases | hsa05168 | Herpes simplex virus 1 infection | 227/2670 | 498/8108 | 9.11E-10 | 4.98E-08 |
|  |  | hsa05165 | Human papillomavirus infection | 155/2670 | 331/8108 | 5.79E-08 | 1.46E-06 |
|  |  | hsa05100 | Bacterial invasion of epithelial cells | 47/2670 | 77/8108 | 3.52E-07 | 6.79E-06 |
|  |  | hsa05132 | Salmonella infection | 119/2670 | 249/8108 | 5.56E-07 | 9.61E-06 |
|  |  | hsa05131 | Shigellosis | 118/2670 | 247/8108 | 6.36E-07 | 1.04E-05 |
|  |  | hsa05135 | Yersinia infection | 63/2670 | 137/8108 | 9.14E-04 | 5.18E-03 |
|  |  | hsa05130 | Pathogenic Escherichia coli infection | 85/2670 | 197/8108 | 1.55E-03 | 8.06E-03 |
|  |  | hsa05120 | Epithelial cell signaling in Helicobacter pylori infection | 33/2670 | 70/8108 | 9.09E-03 | 3.73E-02 |
|  | Other | hsa04510 | Focal adhesion | 117/2670 | 201/8108 | 8.54E-14 | 2.80E-11 |
|  |  | hsa04140 | Autophagy - animal | 85/2670 | 141/8108 | 1.74E-11 | 1.90E-09 |
|  |  | hsa04137 | Mitophagy - animal | 45/2670 | 72/8108 | 2.34E-07 | 4.80E-06 |
|  |  | hsa05412 | Arrhythmogenic right ventricular cardiomyopathy | 42/2670 | 77/8108 | 7.03E-05 | 6.23E-04 |
|  |  | hsa04933 | AGE-RAGE signaling pathway in diabetic complications | 49/2670 | 100/8108 | 5.83E-04 | 3.61E-03 |
|  |  | hsa05415 | Diabetic cardiomyopathy | 89/2670 | 203/8108 | 6.61E-04 | 4.01E-03 |
|  |  | hsa04136 | Autophagy - other | 18/2670 | 32/8108 | 5.45E-03 | 2.51E-02 |
|  |  | hsa05418 | Fluid shear stress and atherosclerosis | 59/2670 | 139/8108 | 1.13E-02 | 4.46E-02 |
| *CLK1* | Cancer-related pathway | hsa04120 | Ubiquitin mediated proteolysis | 85/2781 | 140/8108 | 1.12E-10 | 1.85E-08 |
|  |  | hsa05231 | Choline metabolism in cancer | 49/2781 | 98/8108 | 9.18E-04 | 2.34E-02 |
|  |  | hsa05211 | Renal cell carcinoma | 36/2781 | 69/8108 | 1.63E-03 | 3.37E-02 |
|  | Metabolism | hsa04150 | mTOR signaling pathway | 70/2781 | 155/8108 | 3.03E-03 | 4.76E-02 |
|  |  | hsa04144 | Endocytosis | 122/2781 | 252/8108 | 1.89E-06 | 1.56E-04 |
|  |  | hsa03040 | Spliceosome | 74/2781 | 147/8108 | 3.89E-05 | 2.14E-03 |
|  |  | hsa03015 | mRNA surveillance pathway | 52/2781 | 98/8108 | 9.58E-05 | 4.51E-03 |
|  |  | hsa04141 | Protein processing in endoplasmic reticulum | 81/2781 | 171/8108 | 2.46E-04 | 1.01E-02 |
|  |  | hsa04910 | Insulin signaling pathway | 65/2781 | 137/8108 | 9.19E-04 | 2.34E-02 |
|  |  | hsa04218 | Cellular senescence | 72/2781 | 156/8108 | 1.31E-03 | 3.01E-02 |
|  |  | hsa04070 | Phosphatidylinositol signaling system | 48/2781 | 97/8108 | 1.37E-03 | 3.01E-02 |
|  |  | hsa04068 | FoxO signaling pathway | 61/2781 | 131/8108 | 2.29E-03 | 4.44E-02 |
|  |  | hsa04114 | Oocyte meiosis | 60/2781 | 129/8108 | 2.56E-03 | 4.55E-02 |
|  |  | hsa04012 | ErbB signaling pathway | 42/2781 | 85/8108 | 2.75E-03 | 4.55E-02 |
|  |  | hsa00562 | Inositol phosphate metabolism | 37/2781 | 73/8108 | 2.76E-03 | 4.55E-02 |
|  | Infectious diseases | hsa05168 | Herpes simplex virus 1 infection | 338/2781 | 498/8108 | 4.58E-56 | 1.51E-53 |
|  | Other | hsa04140 | Autophagy - animal | 75/2781 | 141/8108 | 2.65E-06 | 1.75E-04 |
|  |  | hsa04137 | Mitophagy - animal | 38/2781 | 72/8108 | 9.23E-04 | 2.34E-02 |
|  |  | hsa03018 | RNA degradation | 48/2781 | 79/8108 | 1.25E-06 | 1.38E-04 |
|  |  | hsa03013 | RNA transport | 86/2781 | 186/8108 | 4.37E-04 | 1.44E-02 |
|  |  | hsa03022 | Basal transcription factors | 27/2781 | 45/8108 | 3.61E-04 | 1.32E-02 |
| *SFPQ* | Cancer-related pathway | hsa04120 | Ubiquitin mediated proteolysis | 85/2485 | 140/8108 | 1.24E-13 | 1.02E-11 |
|  |  | hsa05220 | Chronic myeloid leukemia | 45/2485 | 76/8108 | 2.16E-07 | 5.92E-06 |
|  |  | hsa05210 | Colorectal cancer | 47/2485 | 86/8108 | 2.87E-06 | 5.56E-05 |
|  |  | hsa05212 | Pancreatic cancer | 41/2485 | 76/8108 | 1.86E-05 | 2.92E-04 |
|  |  | hsa05211 | Renal cell carcinoma | 36/2485 | 69/8108 | 1.48E-04 | 1.74E-03 |
|  |  | hsa05215 | Prostate cancer | 47/2485 | 97/8108 | 1.66E-04 | 1.88E-03 |
|  |  | hsa05213 | Endometrial cancer | 31/2485 | 58/8108 | 2.39E-04 | 2.62E-03 |
|  |  | hsa04150 | mTOR signaling pathway | 67/2485 | 155/8108 | 5.68E-04 | 5.66E-03 |
|  |  | hsa04115 | p53 signaling pathway | 35/2485 | 73/8108 | 1.38E-03 | 1.16E-02 |
|  |  | hsa05225 | Hepatocellular carcinoma | 70/2485 | 168/8108 | 1.46E-03 | 1.20E-02 |
|  |  | hsa05223 | Non-small cell lung cancer | 33/2485 | 72/8108 | 4.61E-03 | 3.22E-02 |
|  |  | hsa01521 | EGFR tyrosine kinase inhibitor resistance | 35/2485 | 79/8108 | 6.97E-03 | 4.42E-02 |
|  |  | hsa03030 | DNA replication | 25/2485 | 36/8108 | 1.79E-06 | 4.22E-05 |
|  | Neural signaling pathway | hsa05014 | Amyotrophic lateral sclerosis | 150/2485 | 365/8108 | 9.71E-06 | 1.68E-04 |
|  |  | hsa05017 | Spinocerebellar ataxia | 66/2485 | 143/8108 | 6.10E-05 | 8.37E-04 |
|  |  | hsa05016 | Huntington disease | 114/2485 | 306/8108 | 6.99E-03 | 4.42E-02 |
|  | Metabolism | hsa03040 | Spliceosome | 92/2485 | 147/8108 | 7.86E-16 | 1.29E-13 |
|  |  | hsa04110 | Cell cycle | 80/2485 | 124/8108 | 5.15E-15 | 5.64E-13 |
|  |  | hsa00310 | Lysine degradation | 37/2485 | 63/8108 | 3.39E-06 | 6.20E-05 |
|  |  | hsa04520 | Adherens junction | 39/2485 | 71/8108 | 1.70E-05 | 2.79E-04 |
|  |  | hsa04068 | FoxO signaling pathway | 61/2485 | 131/8108 | 8.35E-05 | 1.06E-03 |
|  |  | hsa04141 | Protein processing in endoplasmic reticulum | 88/2485 | 171/8108 | 8.02E-09 | 3.30E-07 |
|  |  | hsa03440 | Homologous recombination | 30/2485 | 41/8108 | 2.39E-08 | 8.74E-07 |
|  |  | hsa03015 | mRNA surveillance pathway | 56/2485 | 98/8108 | 4.17E-08 | 1.37E-06 |
|  |  | hsa04218 | Cellular senescence | 78/2485 | 156/8108 | 2.71E-07 | 6.87E-06 |
|  |  | hsa03420 | Nucleotide excision repair | 30/2485 | 47/8108 | 2.60E-06 | 5.56E-05 |
|  |  | hsa03022 | Basal transcription factors | 29/2485 | 45/8108 | 2.87E-06 | 5.56E-05 |
|  |  | hsa04114 | Oocyte meiosis | 61/2485 | 129/8108 | 4.76E-05 | 6.81E-04 |
|  |  | hsa04144 | Endocytosis | 106/2485 | 252/8108 | 6.49E-05 | 8.54E-04 |
|  |  | hsa04012 | ErbB signaling pathway | 43/2485 | 85/8108 | 8.98E-05 | 1.09E-03 |
|  |  | hsa00970 | Aminoacyl-tRNA biosynthesis | 34/2485 | 66/8108 | 3.08E-04 | 3.27E-03 |
|  |  | hsa04914 | Progesterone-mediated oocyte maturation | 47/2485 | 100/8108 | 4.05E-04 | 4.16E-03 |
|  |  | hsa04070 | Phosphatidylinositol signaling system | 45/2485 | 97/8108 | 7.55E-04 | 7.31E-03 |
|  |  | hsa00562 | Inositol phosphate metabolism | 35/2485 | 73/8108 | 1.38E-03 | 1.16E-02 |
|  |  | hsa03008 | Ribosome biogenesis in eukaryotes | 48/2485 | 110/8108 | 2.58E-03 | 2.02E-02 |
|  |  | hsa03060 | Protein export | 14/2485 | 23/8108 | 2.58E-03 | 2.02E-02 |
|  |  | hsa04130 | SNARE interactions in vesicular transport | 18/2485 | 33/8108 | 3.58E-03 | 2.68E-02 |
|  |  | hsa00450 | Selenocompound metabolism | 11/2485 | 17/8108 | 3.87E-03 | 2.76E-02 |
|  |  | hsa03450 | Non-homologous end-joining | 9/2485 | 13/8108 | 4.70E-03 | 3.22E-02 |
|  |  | hsa00670 | One carbon pool by folate | 12/2485 | 20/8108 | 6.17E-03 | 4.09E-02 |
|  | Infectious diseases | hsa05168 | Herpes simplex virus 1 infection | 283/2485 | 498/8108 | 6.35E-36 | 2.09E-33 |
|  |  | hsa05131 | Shigellosis | 99/2485 | 247/8108 | 8.72E-04 | 8.20E-03 |
|  |  | hsa05161 | Hepatitis B | 68/2485 | 162/8108 | 1.35E-03 | 1.16E-02 |
|  |  | hsa05166 | Human T-cell leukemia virus 1 infection | 86/2485 | 219/8108 | 3.66E-03 | 2.68E-02 |
|  |  | hsa05132 | Salmonella infection | 95/2485 | 249/8108 | 6.22E-03 | 4.09E-02 |
|  | Other | hsa04140 | Autophagy - animal | 73/2485 | 141/8108 | 1.10E-07 | 3.28E-06 |
|  |  | hsa01524 | Platinum drug resistance | 35/2485 | 73/8108 | 1.38E-03 | 1.16E-02 |
|  |  | hsa03013 | RNA transport | 104/2485 | 186/8108 | 4.42E-13 | 2.91E-11 |
|  |  | hsa03460 | Fanconi anemia pathway | 42/2485 | 54/8108 | 1.08E-12 | 5.92E-11 |
|  |  | hsa03018 | RNA degradation | 49/2485 | 79/8108 | 7.30E-09 | 3.30E-07 |
|  |  | hsa03430 | Mismatch repair | 17/2485 | 23/8108 | 2.37E-05 | 3.54E-04 |
|  |  | hsa03410 | Base excision repair | 18/2485 | 33/8108 | 3.58E-03 | 2.68E-02 |
| *UBP1* | Cancer-related pathway | hsa03030 | DNA replication | 14/901 | 36/8108 | 1.43E-05 | 4.11E-04 |
|  | Neural signaling pathway | hsa05012 | Parkinson disease | 65/901 | 249/8108 | 1.60E-11 | 1.69E-09 |
|  |  | hsa05016 | Huntington disease | 70/901 | 306/8108 | 1.57E-09 | 9.94E-08 |
|  |  | hsa05014 | Amyotrophic lateral sclerosis | 80/901 | 365/8108 | 8.88E-10 | 7.01E-08 |
|  |  | hsa05022 | Pathways of neurodegeneration - multiple diseases | 94/901 | 476/8108 | 8.87E-09 | 4.01E-07 |
|  |  | hsa05010 | Alzheimer disease | 74/901 | 369/8108 | 1.99E-07 | 7.88E-06 |
|  |  | hsa05017 | Spinocerebellar ataxia | 35/901 | 143/8108 | 4.24E-06 | 1.49E-04 |
|  | Immune signaling pathway | hsa05320 | Autoimmune thyroid disease | 15/901 | 53/8108 | 4.62E-04 | 1.04E-02 |
|  |  | hsa04612 | Antigen processing and presentation | 19/901 | 78/8108 | 6.96E-04 | 1.47E-02 |
|  | Metabolism | hsa03050 | Proteasome | 25/901 | 46/8108 | 7.15E-13 | 2.26E-10 |
|  |  | hsa01200 | Carbon metabolism | 30/901 | 115/8108 | 5.20E-06 | 1.64E-04 |
|  |  | hsa00190 | Oxidative phosphorylation | 29/901 | 134/8108 | 2.90E-04 | 7.63E-03 |
|  |  | hsa01230 | Biosynthesis of amino acids | 19/901 | 75/8108 | 4.10E-04 | 9.98E-03 |
|  |  | hsa00510 | N-Glycan biosynthesis | 14/901 | 50/8108 | 7.98E-04 | 1.48E-02 |
|  |  | hsa00010 | Glycolysis / Gluconeogenesis | 17/901 | 67/8108 | 7.99E-04 | 1.48E-02 |
|  |  | hsa00020 | Citrate cycle (TCA cycle) | 10/901 | 30/8108 | 1.02E-03 | 1.79E-02 |
|  | Infectious diseases | hsa05168 | Herpes simplex virus 1 infection | 108/901 | 498/8108 | 1.47E-12 | 2.33E-10 |
|  |  | hsa05020 | Prion disease | 64/901 | 273/8108 | 2.90E-09 | 1.53E-07 |
|  | Other | hsa05415 | Diabetic cardiomyopathy | 37/901 | 203/8108 | 1.55E-03 | 2.54E-02 |
|  |  | hsa05330 | Allograft rejection | 11/901 | 38/8108 | 2.13E-03 | 3.21E-02 |
|  |  | hsa04932 | Non-alcoholic fatty liver disease | 30/901 | 155/8108 | 1.61E-03 | 2.54E-02 |
| *PDCD6IP* | Cancer-related pathway | hsa04120 | Ubiquitin mediated proteolysis | 96/2869 | 140/8108 | 8.24E-16 | 1.36E-13 |
|  |  | hsa05210 | Colorectal cancer | 50/2869 | 86/8108 | 1.27E-05 | 2.09E-04 |
|  |  | hsa05220 | Chronic myeloid leukemia | 45/2869 | 76/8108 | 1.80E-05 | 2.69E-04 |
|  |  | hsa05211 | Renal cell carcinoma | 41/2869 | 69/8108 | 3.74E-05 | 5.15E-04 |
|  |  | hsa05212 | Pancreatic cancer | 44/2869 | 76/8108 | 4.80E-05 | 6.09E-04 |
|  |  | hsa01521 | EGFR tyrosine kinase inhibitor resistance | 44/2869 | 79/8108 | 1.67E-04 | 1.62E-03 |
|  |  | hsa05215 | Prostate cancer | 50/2869 | 97/8108 | 7.49E-04 | 5.64E-03 |
|  |  | hsa05231 | Choline metabolism in cancer | 50/2869 | 98/8108 | 1.01E-03 | 7.25E-03 |
|  |  | hsa05223 | Non-small cell lung cancer | 37/2869 | 72/8108 | 3.73E-03 | 2.12E-02 |
|  |  | hsa05213 | Endometrial cancer | 30/2869 | 58/8108 | 7.61E-03 | 3.81E-02 |
|  |  | hsa05214 | Glioma | 38/2869 | 75/8108 | 4.54E-03 | 2.49E-02 |
|  | Neural signaling pathway | hsa05014 | Amyotrophic lateral sclerosis | 174/2869 | 365/8108 | 5.34E-07 | 1.60E-05 |
|  |  | hsa04071 | Sphingolipid signaling pathway | 68/2869 | 119/8108 | 8.85E-07 | 2.09E-05 |
|  |  | hsa04722 | Neurotrophin signaling pathway | 65/2869 | 119/8108 | 1.22E-05 | 2.09E-04 |
|  |  | hsa04720 | Long-term potentiation | 37/2869 | 67/8108 | 6.74E-04 | 5.29E-03 |
|  |  | hsa05010 | Alzheimer disease | 159/2869 | 369/8108 | 1.05E-03 | 7.36E-03 |
|  |  | hsa05016 | Huntington disease | 134/2869 | 306/8108 | 1.20E-03 | 8.25E-03 |
|  |  | hsa05022 | Pathways of neurodegeneration - multiple diseases | 199/2869 | 476/8108 | 1.63E-03 | 1.10E-02 |
|  |  | hsa05017 | Spinocerebellar ataxia | 68/2869 | 143/8108 | 1.68E-03 | 1.11E-02 |
|  | Metabolism | hsa04141 | Protein processing in endoplasmic reticulum | 98/2869 | 171/8108 | 2.88E-09 | 2.38E-07 |
|  |  | hsa04144 | Endocytosis | 132/2869 | 252/8108 | 1.54E-08 | 1.02E-06 |
|  |  | hsa04218 | Cellular senescence | 89/2869 | 156/8108 | 2.07E-08 | 1.14E-06 |
|  |  | hsa04068 | FoxO signaling pathway | 75/2869 | 131/8108 | 2.20E-07 | 9.08E-06 |
|  |  | hsa04150 | mTOR signaling pathway | 85/2869 | 155/8108 | 4.63E-07 | 1.53E-05 |
|  |  | hsa03040 | Spliceosome | 81/2869 | 147/8108 | 6.60E-07 | 1.82E-05 |
|  |  | hsa04070 | Phosphatidylinositol signaling system | 58/2869 | 97/8108 | 7.25E-07 | 1.84E-05 |
|  |  | hsa04520 | Adherens junction | 45/2869 | 71/8108 | 1.32E-06 | 2.73E-05 |
|  |  | hsa04910 | Insulin signaling pathway | 75/2869 | 137/8108 | 2.37E-06 | 4.60E-05 |
|  |  | hsa03015 | mRNA surveillance pathway | 56/2869 | 98/8108 | 8.05E-06 | 1.48E-04 |
|  |  | hsa04110 | Cell cycle | 67/2869 | 124/8108 | 1.45E-05 | 2.28E-04 |
|  |  | hsa00562 | Inositol phosphate metabolism | 43/2869 | 73/8108 | 3.28E-05 | 4.70E-04 |
|  |  | hsa04211 | Longevity regulating pathway | 50/2869 | 89/8108 | 4.52E-05 | 5.97E-04 |
|  |  | hsa04114 | Oocyte meiosis | 67/2869 | 129/8108 | 7.63E-05 | 9.32E-04 |
|  |  | hsa04152 | AMPK signaling pathway | 63/2869 | 120/8108 | 8.20E-05 | 9.65E-04 |
|  |  | hsa04714 | Thermogenesis | 110/2869 | 232/8108 | 8.77E-05 | 9.65E-04 |
|  |  | hsa04012 | ErbB signaling pathway | 47/2869 | 85/8108 | 1.28E-04 | 1.32E-03 |
|  |  | hsa03022 | Basal transcription factors | 28/2869 | 45/8108 | 2.13E-04 | 1.85E-03 |
|  |  | hsa04530 | Tight junction | 82/2869 | 169/8108 | 2.67E-04 | 2.26E-03 |
|  |  | hsa04931 | Insulin resistance | 55/2869 | 108/8108 | 6.14E-04 | 4.94E-03 |
|  |  | hsa04710 | Circadian rhythm | 20/2869 | 31/8108 | 8.86E-04 | 6.50E-03 |
|  |  | hsa03450 | Non-homologous end-joining | 10/2869 | 13/8108 | 2.74E-03 | 1.67E-02 |
|  |  | hsa04371 | Apelin signaling pathway | 65/2869 | 138/8108 | 2.81E-03 | 1.67E-02 |
|  |  | hsa04919 | Thyroid hormone signaling pathway | 58/2869 | 121/8108 | 2.84E-03 | 1.67E-02 |
|  |  | hsa04810 | Regulation of actin cytoskeleton | 97/2869 | 218/8108 | 3.04E-03 | 1.76E-02 |
|  |  | hsa03440 | Homologous recombination | 23/2869 | 41/8108 | 5.25E-03 | 2.80E-02 |
|  |  | hsa04213 | Longevity regulating pathway - multiple species | 32/2869 | 62/8108 | 6.20E-03 | 3.25E-02 |
|  |  | hsa04130 | SNARE interactions in vesicular transport | 19/2869 | 33/8108 | 7.48E-03 | 3.80E-02 |
|  |  | hsa00600 | Sphingolipid metabolism | 26/2869 | 49/8108 | 8.26E-03 | 4.07E-02 |
|  |  | hsa00310 | Lysine degradation | 32/2869 | 63/8108 | 8.40E-03 | 4.08E-02 |
|  |  | hsa04935 | Growth hormone synthesis, secretion and action | 55/2869 | 119/8108 | 9.15E-03 | 4.38E-02 |
|  |  | hsa04914 | Progesterone-mediated oocyte maturation | 47/2869 | 100/8108 | 1.06E-02 | 5.00E-02 |
|  | Infectious diseases | hsa05168 | Herpes simplex virus 1 infection | 280/2869 | 498/8108 | 9.91E-23 | 3.27E-20 |
|  |  | hsa05132 | Salmonella infection | 128/2869 | 249/8108 | 1.07E-07 | 5.03E-06 |
|  |  | hsa05131 | Shigellosis | 125/2869 | 247/8108 | 4.59E-07 | 1.53E-05 |
|  |  | hsa05100 | Bacterial invasion of epithelial cells | 43/2869 | 77/8108 | 1.82E-04 | 1.72E-03 |
|  |  | hsa05135 | Yersinia infection | 69/2869 | 137/8108 | 2.03E-04 | 1.85E-03 |
|  |  | hsa05130 | Pathogenic Escherichia coli infection | 94/2869 | 197/8108 | 2.11E-04 | 1.85E-03 |
|  |  | hsa05161 | Hepatitis B | 75/2869 | 162/8108 | 2.50E-03 | 1.61E-02 |
|  |  | hsa05170 | Human immunodeficiency virus 1 infection | 95/2869 | 212/8108 | 2.57E-03 | 1.63E-02 |
|  |  | hsa05163 | Human cytomegalovirus infection | 100/2869 | 225/8108 | 2.76E-03 | 1.67E-02 |
|  |  | hsa05120 | Epithelial cell signaling in Helicobacter pylori infection | 36/2869 | 70/8108 | 4.13E-03 | 2.31E-02 |
|  |  | hsa05110 | Vibrio cholerae infection | 27/2869 | 50/8108 | 5.26E-03 | 2.80E-02 |
|  |  | hsa05160 | Hepatitis C | 71/2869 | 157/8108 | 6.47E-03 | 3.34E-02 |
|  | Other | hsa04140 | Autophagy - animal | 96/2869 | 141/8108 | 1.69E-15 | 1.86E-13 |
|  |  | hsa04136 | Autophagy - other | 22/2869 | 32/8108 | 1.22E-04 | 1.30E-03 |
|  |  | hsa04932 | Non-alcoholic fatty liver disease | 77/2869 | 155/8108 | 1.59E-04 | 1.59E-03 |
|  |  | hsa04137 | Mitophagy - animal | 40/2869 | 72/8108 | 3.50E-04 | 2.89E-03 |
|  |  | hsa03018 | RNA degradation | 49/2869 | 79/8108 | 1.15E-06 | 2.54E-05 |
|  |  | hsa03013 | RNA transport | 91/2869 | 186/8108 | 8.60E-05 | 9.65E-04 |
|  |  | hsa03460 | Fanconi anemia pathway | 31/2869 | 54/8108 | 7.52E-04 | 5.64E-03 |
| *ZNF248* | Cancer-related pathway | hsa04120 | Ubiquitin mediated proteolysis | 88/3160 | 140/8108 | 7.24E-09 | 7.94E-07 |
|  | Neural signaling pathway | hsa04720 | Long-term potentiation | 42/3160 | 67/8108 | 6.90E-05 | 5.68E-03 |
|  |  | hsa05016 | Huntington disease | 148/3160 | 306/8108 | 4.16E-04 | 1.60E-02 |
|  | Metabolism | hsa03015 | mRNA surveillance pathway | 56/3160 | 98/8108 | 1.89E-04 | 1.04E-02 |
|  |  | hsa03440 | Homologous recombination | 27/3160 | 41/8108 | 4.37E-04 | 1.60E-02 |
|  |  | hsa04218 | Cellular senescence | 80/3160 | 156/8108 | 1.09E-03 | 3.57E-02 |
|  |  | hsa04070 | Phosphatidylinositol signaling system | 53/3160 | 97/8108 | 1.19E-03 | 3.57E-02 |
|  |  | hsa04922 | Glucagon signaling pathway | 57/3160 | 107/8108 | 1.77E-03 | 4.86E-02 |
|  | Infectious diseases | hsa05168 | Herpes simplex virus 1 infection | 350/3160 | 498/8108 | 2.64E-48 | 8.69E-46 |
|  | Other | hsa04740 | Olfactory transduction | 241/3160 | 443/8108 | 1.01E-11 | 1.66E-09 |
|  |  | hsa04140 | Autophagy - animal | 75/3160 | 141/8108 | 3.87E-04 | 1.60E-02 |
|  |  | hsa03018 | RNA degradation | 47/3160 | 79/8108 | 1.67E-04 | 1.04E-02 |
| *SLC25A53* | Metabolism | hsa04070 | Phosphatidylinositol signaling system | 45/2461 | 97/8108 | 6.00E-04 | 4.93E-02 |
|  | Infectious diseases | hsa05168 | Herpes simplex virus 1 infection | 247/2461 | 498/8108 | 1.35E-20 | 4.45E-18 |
|  |  | hsa04740 | Olfactory transduction | 213/2461 | 443/8108 | 6.42E-16 | 1.06E-13 |
|  |  | hsa04340 | Hedgehog signaling pathway | 31/2461 | 56/8108 | 8.24E-05 | 9.04E-03 |
| *CLCC1* | Cancer-related pathway | hsa04120 | Ubiquitin mediated proteolysis | 96/2626 | 140/8108 | 1.03E-18 | 1.70E-16 |
|  |  | hsa05220 | Chronic myeloid leukemia | 46/2626 | 76/8108 | 3.87E-07 | 1.82E-05 |
|  |  | hsa05210 | Colorectal cancer | 48/2626 | 86/8108 | 5.71E-06 | 1.11E-04 |
|  |  | hsa05215 | Prostate cancer | 49/2626 | 97/8108 | 1.49E-04 | 1.89E-03 |
|  |  | hsa05212 | Pancreatic cancer | 40/2626 | 76/8108 | 1.94E-04 | 2.30E-03 |
|  |  | hsa05211 | Renal cell carcinoma | 37/2626 | 69/8108 | 2.03E-04 | 2.30E-03 |
|  |  | hsa05213 | Endometrial cancer | 32/2626 | 58/8108 | 2.71E-04 | 2.98E-03 |
|  |  | hsa05223 | Non-small cell lung cancer | 35/2626 | 72/8108 | 2.94E-03 | 2.36E-02 |
|  | Neural signaling pathway | hsa05017 | Spinocerebellar ataxia | 67/2626 | 143/8108 | 1.96E-04 | 2.30E-03 |
|  |  | hsa05014 | Amyotrophic lateral sclerosis | 144/2626 | 365/8108 | 2.15E-03 | 1.91E-02 |
|  |  | hsa04720 | Long-term potentiation | 33/2626 | 67/8108 | 2.93E-03 | 2.36E-02 |
|  |  | hsa04722 | Neurotrophin signaling pathway | 53/2626 | 119/8108 | 3.50E-03 | 2.68E-02 |
|  | Metabolism | hsa04141 | Protein processing in endoplasmic reticulum | 97/2626 | 171/8108 | 3.13E-11 | 3.43E-09 |
|  |  | hsa04144 | Endocytosis | 120/2626 | 252/8108 | 2.35E-07 | 1.55E-05 |
|  |  | hsa04150 | mTOR signaling pathway | 78/2626 | 155/8108 | 2.26E-06 | 6.20E-05 |
|  |  | hsa03040 | Spliceosome | 77/2626 | 147/8108 | 3.38E-07 | 1.82E-05 |
|  |  | hsa04068 | FoxO signaling pathway | 69/2626 | 131/8108 | 1.05E-06 | 3.83E-05 |
|  |  | hsa00310 | Lysine degradation | 39/2626 | 63/8108 | 1.32E-06 | 4.24E-05 |
|  |  | hsa04218 | Cellular senescence | 79/2626 | 156/8108 | 1.42E-06 | 4.24E-05 |
|  |  | hsa00562 | Inositol phosphate metabolism | 43/2626 | 73/8108 | 2.59E-06 | 6.56E-05 |
|  |  | hsa04520 | Adherens junction | 42/2626 | 71/8108 | 2.90E-06 | 6.82E-05 |
|  |  | hsa04070 | Phosphatidylinositol signaling system | 52/2626 | 97/8108 | 1.14E-05 | 1.87E-04 |
|  |  | hsa04110 | Cell cycle | 62/2626 | 124/8108 | 3.13E-05 | 4.67E-04 |
|  |  | hsa03015 | mRNA surveillance pathway | 53/2626 | 98/8108 | 6.69E-06 | 1.22E-04 |
|  |  | hsa04910 | Insulin signaling pathway | 67/2626 | 137/8108 | 3.78E-05 | 5.41E-04 |
|  |  | hsa03022 | Basal transcription factors | 27/2626 | 45/8108 | 1.23E-04 | 1.69E-03 |
|  |  | hsa03060 | Protein export | 16/2626 | 23/8108 | 2.81E-04 | 2.98E-03 |
|  |  | hsa04012 | ErbB signaling pathway | 43/2626 | 85/8108 | 3.57E-04 | 3.67E-03 |
|  |  | hsa01521 | EGFR tyrosine kinase inhibitor resistance | 40/2626 | 79/8108 | 5.53E-04 | 5.51E-03 |
|  |  | hsa04130 | SNARE interactions in vesicular transport | 20/2626 | 33/8108 | 7.78E-04 | 7.53E-03 |
|  |  | hsa04071 | Sphingolipid signaling pathway | 55/2626 | 119/8108 | 1.06E-03 | 9.94E-03 |
|  |  | hsa04152 | AMPK signaling pathway | 54/2626 | 120/8108 | 2.45E-03 | 2.12E-02 |
|  |  | hsa04919 | Thyroid hormone signaling pathway | 54/2626 | 121/8108 | 3.06E-03 | 2.40E-02 |
|  |  | hsa00563 | Glycosylphosphatidylinositol (GPI)-anchor biosynthesis | 15/2626 | 26/8108 | 6.76E-03 | 4.73E-02 |
|  |  | hsa04917 | Prolactin signaling pathway | 33/2626 | 70/8108 | 6.90E-03 | 4.73E-02 |
|  |  | hsa03450 | Non-homologous end-joining | 9/2626 | 13/8108 | 7.10E-03 | 4.77E-02 |
|  |  | hsa00280 | Valine, leucine and isoleucine degradation | 25/2626 | 48/8108 | 3.58E-03 | 2.68E-02 |
|  | Infectious diseases | hsa05168 | Herpes simplex virus 1 infection | 269/2626 | 498/8108 | 6.91E-25 | 2.27E-22 |
|  |  | hsa05131 | Shigellosis | 111/2626 | 247/8108 | 1.95E-05 | 3.05E-04 |
|  |  | hsa05132 | Salmonella infection | 108/2626 | 249/8108 | 1.49E-04 | 1.89E-03 |
|  |  | hsa05161 | Hepatitis B | 68/2626 | 162/8108 | 6.10E-03 | 4.36E-02 |
|  | Other | hsa04140 | Autophagy - animal | 82/2626 | 141/8108 | 1.98E-10 | 1.63E-08 |
|  |  | hsa04136 | Autophagy - other | 19/2626 | 32/8108 | 1.49E-03 | 1.36E-02 |
|  |  | hsa04710 | Circadian rhythm | 18/2626 | 31/8108 | 2.79E-03 | 2.36E-02 |
|  |  | hsa04137 | Mitophagy - animal | 34/2626 | 72/8108 | 5.96E-03 | 4.36E-02 |
|  |  | hsa03013 | RNA transport | 90/2626 | 186/8108 | 3.30E-06 | 7.23E-05 |
|  |  | hsa03018 | RNA degradation | 45/2626 | 79/8108 | 5.36E-06 | 1.10E-04 |
|  |  | hsa03440 | Homologous recombination | 27/2626 | 41/8108 | 1.10E-05 | 1.87E-04 |
|  |  | hsa03460 | Fanconi anemia pathway | 35/2626 | 54/8108 | 9.63E-07 | 3.83E-05 |
| *GPR125* | Cancer-related pathway | hsa04120 | Ubiquitin mediated proteolysis | 81/2345 | 140/8108 | 5.86E-13 | 9.66E-11 |
|  |  | hsa05220 | Chronic myeloid leukemia | 39/2345 | 76/8108 | 3.09E-05 | 8.49E-04 |
|  |  | hsa05210 | Colorectal cancer | 40/2345 | 86/8108 | 3.84E-04 | 6.66E-03 |
|  |  | hsa05223 | Non-small cell lung cancer | 33/2345 | 72/8108 | 1.64E-03 | 2.17E-02 |
|  |  | hsa05231 | Choline metabolism in cancer | 42/2345 | 98/8108 | 2.13E-03 | 2.33E-02 |
|  |  | hsa05212 | Pancreatic cancer | 34/2345 | 76/8108 | 2.33E-03 | 2.33E-02 |
|  |  | hsa05213 | Endometrial cancer | 27/2345 | 58/8108 | 3.21E-03 | 2.86E-02 |
|  |  | hsa05211 | Renal cell carcinoma | 31/2345 | 69/8108 | 3.29E-03 | 2.86E-02 |
|  |  | hsa05221 | Acute myeloid leukemia | 30/2345 | 67/8108 | 4.04E-03 | 3.18E-02 |
|  | Neural signaling pathway | hsa04071 | Sphingolipid signaling pathway | 50/2345 | 119/8108 | 1.44E-03 | 2.00E-02 |
|  |  | hsa05017 | Spinocerebellar ataxia | 57/2345 | 143/8108 | 3.01E-03 | 2.76E-02 |
|  |  | hsa05014 | Amyotrophic lateral sclerosis | 129/2345 | 365/8108 | 3.82E-03 | 3.15E-02 |
|  | Metabolism | hsa04144 | Endocytosis | 112/2345 | 252/8108 | 7.54E-08 | 8.29E-06 |
|  |  | hsa03015 | mRNA surveillance pathway | 51/2345 | 98/8108 | 1.13E-06 | 9.30E-05 |
|  |  | hsa00562 | Inositol phosphate metabolism | 39/2345 | 73/8108 | 9.00E-06 | 4.95E-04 |
|  |  | hsa04070 | Phosphatidylinositol signaling system | 48/2345 | 97/8108 | 1.40E-05 | 5.35E-04 |
|  |  | hsa04218 | Cellular senescence | 70/2345 | 156/8108 | 1.41E-05 | 5.35E-04 |
|  |  | hsa04910 | Insulin signaling pathway | 63/2345 | 137/8108 | 1.46E-05 | 5.35E-04 |
|  |  | hsa04012 | ErbB signaling pathway | 43/2345 | 85/8108 | 1.94E-05 | 6.42E-04 |
|  |  | hsa00310 | Lysine degradation | 34/2345 | 63/8108 | 2.53E-05 | 7.59E-04 |
|  |  | hsa04068 | FoxO signaling pathway | 59/2345 | 131/8108 | 5.72E-05 | 1.35E-03 |
|  |  | hsa04520 | Adherens junction | 36/2345 | 71/8108 | 8.54E-05 | 1.88E-03 |
|  |  | hsa03040 | Spliceosome | 62/2345 | 147/8108 | 3.66E-04 | 6.66E-03 |
|  |  | hsa04150 | mTOR signaling pathway | 65/2345 | 155/8108 | 3.23E-04 | 6.28E-03 |
|  |  | hsa04130 | SNARE interactions in vesicular transport | 19/2345 | 33/8108 | 5.35E-04 | 8.41E-03 |
|  |  | hsa04152 | AMPK signaling pathway | 51/2345 | 120/8108 | 9.52E-04 | 1.43E-02 |
|  |  | hsa03440 | Homologous recombination | 21/2345 | 41/8108 | 2.13E-03 | 2.33E-02 |
|  |  | hsa04110 | Cell cycle | 51/2345 | 124/8108 | 2.25E-03 | 2.33E-02 |
|  |  | hsa04141 | Protein processing in endoplasmic reticulum | 67/2345 | 171/8108 | 2.29E-03 | 2.33E-02 |
|  |  | hsa01521 | EGFR tyrosine kinase inhibitor resistance | 35/2345 | 79/8108 | 2.48E-03 | 2.41E-02 |
|  |  | hsa04931 | Insulin resistance | 45/2345 | 108/8108 | 2.95E-03 | 2.76E-02 |
|  |  | hsa04211 | Longevity regulating pathway | 38/2345 | 89/8108 | 3.66E-03 | 3.10E-02 |
|  |  | hsa00280 | Valine, leucine and isoleucine degradation | 23/2345 | 48/8108 | 4.04E-03 | 3.18E-02 |
|  | Infectious diseases | hsa05168 | Herpes simplex virus 1 infection | 294/2345 | 498/8108 | 1.83E-47 | 6.03E-45 |
|  |  | hsa05166 | Human T-cell leukemia virus 1 infection | 84/2345 | 219/8108 | 1.46E-03 | 2.00E-02 |
|  |  | hsa04917 | Prolactin signaling pathway | 32/2345 | 70/8108 | 2.02E-03 | 2.33E-02 |
|  |  | hsa05132 | Salmonella infection | 93/2345 | 249/8108 | 2.19E-03 | 2.33E-02 |
|  |  | hsa05163 | Human cytomegalovirus infection | 85/2345 | 225/8108 | 2.29E-03 | 2.33E-02 |
|  | Other | hsa04140 | Autophagy - animal | 66/2345 | 141/8108 | 4.37E-06 | 2.89E-04 |
|  |  | hsa05416 | Viral myocarditis | 30/2345 | 60/8108 | 4.45E-04 | 7.34E-03 |
|  |  | hsa03013 | RNA transport | 79/2345 | 186/8108 | 4.58E-05 | 1.16E-03 |
|  |  | hsa03018 | RNA degradation | 39/2345 | 79/8108 | 9.37E-05 | 1.93E-03 |
